# Relationships of Ferroptosis and Pyroptosis-Related Genes with Clinical Prognosis and Tumor Immune Microenvironment in Head and Neck Squamous Cell Carcinoma

**DOI:** 10.1155/2022/3713929

**Published:** 2022-10-05

**Authors:** Jiangang Yu, Ying Chen, Xue Pan, Wen Wen

**Affiliations:** ^1^Department of Anesthesiology, The First Hospital of China Medical University, Shenyang, China; ^2^Department of Ultrasound, Xiaoshan Traditional Chinese Medical Hospital, Zhouhang, China; ^3^Department of Obstetrics and Gynecology, Shengjing Hospital of China Medical University, China; ^4^Department of Laboratory Medicine of Shengjing Hospital of China Medical University, Shenyang, China

## Abstract

Ferroptosis and pyroptosis are two new programmed cell death (PCD) modes discovered in recent years. However, the potential value of ferroptosis and pyroptosis-related genes (FPRGs) in prognosis prediction and the tumor immune microenvironment of head and neck squamous cell carcinoma (HNSCC) is still unclear. We obtained 21 significant FPRGs based on the training dataset (TCGA- HNSC) using the univariate Cox and differential expression analysis. The TCGA- HNSC (*n* = 502) dataset was clustered into two group (clusters A and B) based on the 21 significant FPRGs. 1467 differentially expressed genes (DEGs) between cluster A and B were put into univariate Cox and Least absolute shrinkage and selection operator (LASSO) analysis to build a risk model. The predictive capability of the risk model was successfully confirmed by internal validation, external validation, and clinical sample validation. To improve the clinical applicability, a nomogram model combined risk score and clinical information were constructed. Moreover, the patients with lower risk score were characterized by increased immune response and tumor mutation burden (TMB), while the patients with higher risk score were characterized by increased TP53 mutation rate. In conclusion, our comprehensive analysis of the FPRGs revealed their significant role in prognosis prediction and the tumor immune microenvironment. The risk model containing 9 FPRGs could be a potential prognostic markers and effective immunotherapy targets for HNSCC.

## 1. Background

Cell death is closely related to the basic processes of life and is an important way of growth and development, disease progression, and homeostasis of multicellular organisms [1]. In 2015, the Nomenclature Committee on Cell Death (NCCD) classified cell death into programmed cell death (PCD) and un-programmed cell death according to whether the process of death was regulated by procedures [2]. PCD is an active and orderly way of cell death to maintain the stability of internal environment. Specifically, it refers to the suicide protection measures initiated by gene regulation when cells are stimulated by internal and external environmental factors [3]. PCD contains apoptosis, necroptosis, autophagy, pyroptosis, ferroptosis, and other cell death modes, which play an important role in pathogen immunity and cancer cell clearance [4]. Among them, ferroptosis and pyroptosis are two new PCD modes discovered in recent years [5]. Cell pyroptosis is a novel pro-inflammatory programmed cell death mode, which depends on the activation of cysteinyl aspartate specific proteinase (caspase) and its mediated gasdermin D (GSDMD). Activated caspase mediates the hydrolysis of GSDMD into bioactive GSDMD-N, which embedded into the cytoplasmic membrane to form membrane perforation with a diameter of 10~15 nm. This causes increased cell permeability, imbalance of ion compensation, and water inflow into cells from the intercellular substance, resulting in cell swelling, and large release of lactate dehydrogenase and pro-inflammatory cytokines, such as IL-1*β* and IL-18 [6, 7]. Ferroptosis is an iron-dependent regulatory form of cell death, including activation of reactive oxygen species (ROS), iron aggregation, activation of the mitogen-activated protein kinase (MAPK) system, reduced cysteine uptake, and glutathione depletion [8]. Ferroptosis is characterized by excessive accumulation of iron-dependent lipid peroxidation (LPO) on cell membranes, leading to cell necrosis, which can be inhibited by glutathione peroxidase 4 (Gpx4). Morphology of ferroptosis showed a loss of membrane integrity, accompanied by nuclear swelling and mitochondrial shrinkage, increased membrane density and mitochondrial outer membrane rupture [9].

Head and neck squamous cell carcinoma (HNSCC) is an immunosuppressive disease characterized by molecular heterogeneity and tumor-host interaction. Its morbidity and mortality are increasing year by year, and it is now the sixth most common cancer and the eighth leading cause of cancer death worldwide [10]. The treatment of early HNSCC is mainly surgery and radiotherapy, but the 5-year survival rate is less than 40% because most of patients have locally advanced disease at first diagnosis [11]. Platinum-based chemotherapy for advanced HNSCC has a poor prognosis, with a median survival less than 1 year [12]. Therefore, the treatment of HNSCC is in urgent need of new drug breakthrough. Exogenous activation of ferroptosis and pyroptosis has recently been shown to trigger powerful antitumor effects [13]. Some chemotherapy drugs can switch caspase-3-mediated apoptosis to pyroptosis by cleaving GSDME into GSDME-N in GSDME-expressing tumor cells [14, 15]. GSDME, as a tumor suppressor, can improve the antitumor immunity by activation of pyroptosis, while inflammasome activation induced by pyroptosis further enhances the therapeutic efficacy of some immune checkpoint blockers [16, 17]. Ferroptosis can improve the cytotoxicity of cisplatin of resistant HNC cells [18] and the efficacy of radiotherapy [19]. Ferroptosis can be induced by sorafenib, a kinase inhibitor, which has been reported that it can increase the radiosensitivity and antiproliferative effect of cisplatin in HNSCC cells [13, 20]. Therefore, induction of ferroptosis and pyroptosis may provide an effective treatment strategy of HNSCC.

In this study, we systematically investigated the role of ferroptosis and pyroptosis- related genes in prognosis prediction and the tumor immune landscape of HNSCC. We first constructed and validated a risk model based on the ferroptosis and pyroptosis-related genes (FPRGs). The HNSCC patients were clustered into high- and low-risk group based on the median cut-off of risk score. Then, we assessed the clinical features, tumor mutation burden (TMB), cancer stem cell (CSC) characteristics, and immune infiltration in the two groups. This study paves a novel road for prognosis prediction and treatment strategy of HNSCC.

## 2. Materials and Methods

### 2.1. Data Acquisition

**Figure 1:**
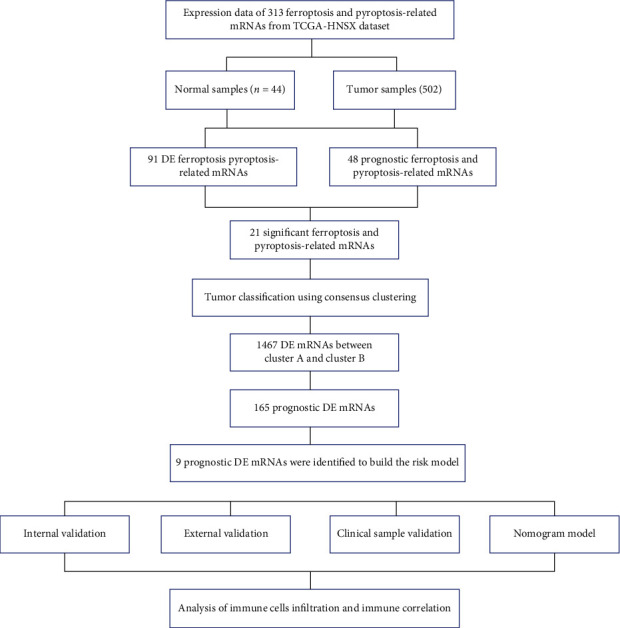
Workflow to construct the ferroptosis and pyroptosis-related risk model in HNSCC patients. TCGA: The Cancer Genome Atlas; HNSCC: head and neck squamous cell carcinoma; DE: differentially expressed.

The workflow of this study was shown in Figure 1. The mRNAs-seq data, somatic mutation data, copy number variation (CNV) data, and corresponding clinical information of TCGA-HNSC dataset including 44 normal samples and 502 HNSCC samples were downloaded from The Cancer Genome Atlas (TCGA) database. The mRNAs-seq data and clinical information of GSE65858 dataset (270 HNSCC samples) based on the platform GPL10558 (Illumina HumanHT-12 V4.0 expression beadchip, Illumina Inc., San Diego, CA, USA) were obtained from the Gene Expression Omnibus (GEO) database. It was generated from samples of peripheral blood mononuclear cells (PBMCs) of patients. The “limma” package was used to normalized the expression profiles data. The baseline information is shown in table 1.

### 2.2. Unsupervised Clustering for FPRGs

We downloaded 313 FPRGs from the predecessors' study [21] and extracted the mRNAs-seq data of the 313 genes from the TCGA-HNSC dataset. The differentially expressed (DE) FPRGs between normal and HNSCC samples were screened using the “limma” package based on the selection criteria of log|FC| ≥ 1 and *p* < 0.05 [5]. The FPRGs with prognostic value (*p* < 0.05) was selected by univariate Cox regression analysis. Then, the DE FPRGs with prognostic value were subjected to consensus clustering algorithm. The “ConsensuClusterPlus” package was performed with 1000 times repetitions to guarantee the stability of classification [22].

### 2.3. GSVA and GO Functional Enrichment Analysis

Gene set variation analysis (GSVA) was used to investigate the differentially activity of molecular pathways between different subtypes using the “GSVA” packages in R software [23]. The gene file of “c2.cp.kegg.v7.4.symbols.gmt” was downloaded from MSigDB database for GSVA analysis, and *p* < 0.05 was considered as statistically significance. The differentially expressed genes (DEGs) between different subtypes were screened using the “limma” package based on the selection criteria of log|FC| ≥ 1 and *p* < 0.05. Then, the DEGs were subjected to univariate Cox regression analysis. The DEGs meet the screening criteria *p* < 0.05 and were considered as significant DEGs for subsequent analysis. Gene Ontology (GO) functional enrichment analysis was performed using the “clusterProfiler” package to explore the potential molecular function of significant DEGs [24].

### 2.4. Construction and Validation of a Prognostic Risk Model

The significant DEGs were included in Least Absolute Shrinkage and Selection Operator (LASSO) regression analysis using the “glmnet” package in R software, and a 10-fold cross-validation/leave-one-out was performed to avoid model overfitting [25]. The significant DEGs with nonzero regression coefficients obtained by LASSO regression analysis were subjected to multivariate Cox regression analysis to further narrow down the genes and build a risk model. The risk score of each patient is calculated using the following formula: risk score = *Σ* (expression value of each gene × and its coefficient). The HNSCC patients were clustered into high- and low-risk group based on the median cut-off of risk score. The Kaplan–Meier (KM) curve was plotted to evaluate the prognosis of the risk model using the “survminer” R package. The receiver operating characteristic (ROC) curves at 1, 3, and 5 years were drawn to assess the prognostic predictive performance of the risk model using the “survival ROC” R package. Univariate and multivariate Cox regression analyses was used to identify whether the risk model is an independent prognostic factor for HNSCC. The TCGA-HNSC dataset was randomly divided into TCGA-training dataset (*n* = 250) and TCGA-testing dataset (*n* = 249) to confirm the performance of the risk model by internal validation. External validation was performed in GSE65858 dataset (*n* = 270). The prognostic predictive performance of the risk model was validated in internal and external validation using the same methods mentioned above.

### 2.5. Tissue Collection

Forty healthy samples and sixty-eight HNSCC samples were collected from Tissue specimen Bank of Shengjing Hospital between 2015 and 2021. None of the selected patients received any radiotherapy, chemotherapy, or other antitumor therapy within 3 months before surgery. The clinical information of the patient is complete (Table 1). This study was approved by the Ethics Committee of Shengjing Hospital of the China Medical University, and informed consent was obtained from all patients. In addition, all methods were performed in accordance with relevant guidelines and regulations.

### 2.6. Quantitative Real-Time RT-PCR

Total RNA from healthy samples and HNSCC samples was extracted by TRIzol (Invitrogen, USA) and reverse-transcribed to cDNA. Real time quantitative polymerase chain reaction (RT-qPCR) was performed based on SYBR Premix Exaq (Takara, Japan). GAPDH was used as an internal reference to calculate the relative expression levels of genes in healthy samples and HNSCC samples according to the 2-*ΔΔ*Ct method. Supplementary Table 1 presents the primer sequences of the genes. We then compared the differential expression level of genes between healthy samples and HNSCC samples. Finally, we validated the prognostic predictive performance of the risk model based on the 68 clinical specimens using the same methods mentioned above.

### 2.7. Construction of a Nomogram Model

To improve the clinical applicability, we constructed a nomogram model combined risk score and clinical information to predict the survival of HNSCC patients at 1, 3, and 5 years using the “rms” R package [26]. Calibration curve was used to assess the differential predicted OS probability between the actual and the nomogram model. Decision curve analysis (DCA) curve and ROC curve were used to compare the differential performance of the nomogram to risk score and clinical information.

### 2.8. Exploration of the Clinicopathological Features and Stemness Characteristics of the Prognostic Risk Model

The “compare” R package was used to compare the risk score in different cluster and clinicopathological features including age, sex, stage, grade, hpv16 status, and alcohol history. Gene mutation rate and tumor mutation burden (TMB) between high- and low-risk groups was compared by Wilcox test. The “maftools” R package was used to visualize the differential gene mutation in high- and low-risk groups [21]. The correlation between TMB and risk score was identified by Spearman correlation analysis. The statistical significance was set at *P* < 0.05.

### 2.9. Tumor Immune Characteristics Analysis

The abundance of tumor immune cell infiltration in HNSCC samples was calculated by single sample gene set enrichment analysis (ssGSEA) algorithm [21]. The “estimate” R package was used to calculate the stromalscore, immunescore, and ESTIMATEScore of the HNSCC samples. Wilcox test was used to compare the differential immune cell infiltration, immune checkpoint genes expression, stromalscore, immunescore, and ESTIMATEScore in different groups (high- vs low-risk group). Spearman correlation analysis was used to analyze the correlation between immune cell infiltration abundance and genes and risk score.

### 2.10. Statistical Analysis

One-way ANOVA and Kruskal-Wallis tests were used to compare differences between groups. Kaplan–Meier (K-M) curve was plotted for prognostic analysis in high- and low-risk groups. The “RCircos” R package was used to present the CNV of the DE FPRGs with prognostic value in chromosomes [27]. The “forestplot” R package was performed to calculate and visualize the hazard ratios (HR) of the DE FPRGs in TCGA-HNSC dataset [22]. All parametric analyses were based on two-tailed tests, the statistical significance of which was set at *P* < 0.05. All statistical analyses were performed using R 4.0.0.

## 3. Results

### 3.1. Landscape of 21 FPRGs in TCGA-HNSC Dataset

**Figure 2:**
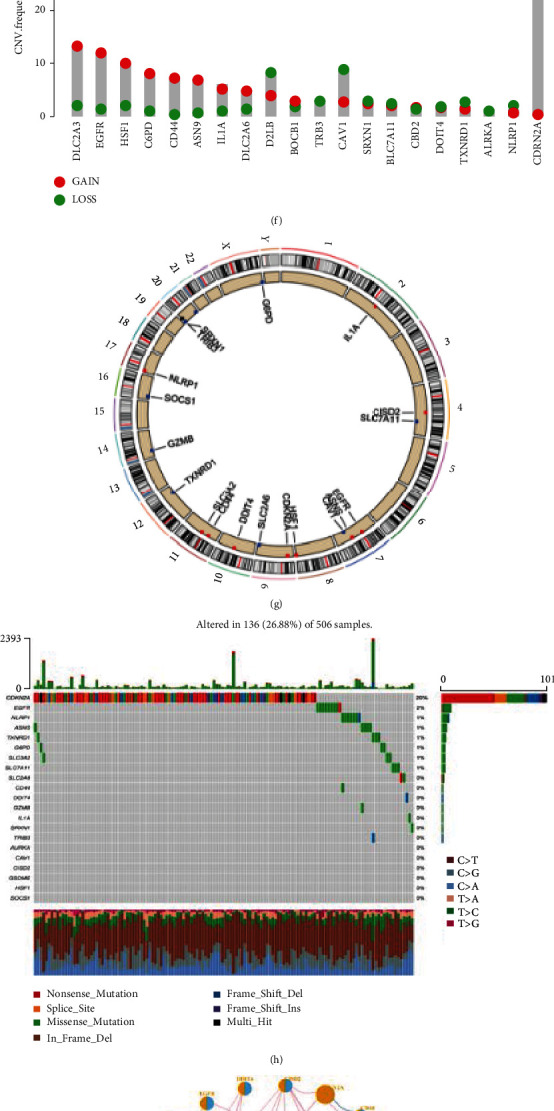
Genetic and transcriptional alterations of FPRGs in HNSCC. (a) Volcano plot of the DEFPRGs. (b) The Venn plot of intersection DEFPRGs and prognostic FPRGs. (c) PCA plot of the HNSCC and normal samples based on 21 FPRGs. (d) Expression heat map of the 21 FPRGs between the HNSCC and normal samples. (e) Differential expression histogram of the 21 FPRGs between HNSCC and normal samples. (f) Frequencies of CNV gain, loss, and non-CNV among the 21 FPRGs. (g) Locations of CNV alterations in the 21 FPRGs on 23 chromosomes. (h) Mutation frequencies of the 21 FPRGs. (i) The correlation network among the 21 FPRGs (the circle size indicates the *p* value of the log-rank test, and the lines linking the 21 FPRGs indicate their interactions). (j) The univariate Cox regression analysis–based forest plot in 21 FPRGs. DEFPRGs: differentially expressed ferroptosis and pyroptosis-related genes; TCGA: The Cancer Genome Atlas; HNSCC: head and neck squamous cell carcinoma; CNV: copy number variant; PCA: principal component analysis.

We obtained 91 DE FPRGs between normal and HNSCC samples through differential expression analysis (Figure 2(a), Supplementary table 2) and 48 prognostic related ferroptosis and pyroptosis by univariate Cox regression analysis (Supplementary table 3). Then, 21 integrated FPRGs were required and visualized by Venn diagram (Figure 2(b)). Principal component analysis (PCA) revealed that we can completely distinguished HNSCC samples from the normal samples based on the expression level of the 21 FPRGs (Figure 2(c)). The heat map and histogram showed that the 21 FPRGs were highly expressed in HNSCC samples compared with normal samples (Figures 2(d) and 2(e)). CNV alteration frequency showed that CNV alterations were common in the 21 FPRGs, with maximum frequency CNV amplification in SLC3A2 and maximum frequency CNV deletion in CDKN2A (Figure 2(f)).  Figure 2(g) presented the CNV alteration of the 21 FPRGs in chromosomes. Oncoplots of the 21 FPRGs indicated that the CDKN2A with mutation frequency 20% was highest, while the other 20 FPRGs have fewer mutation frequency (Figure 2(h)). The network depicted the interactions, regulator connection, and prognostic value of the 21 FPRGs in TCGA-HNSC dataset (Figure 2(i)). It was found that there was a general positive correlation between the 21 genes, among which G6PD had the strongest positive correlation with SRXN1. Forest map presented the prognostic values of 21 FPRGs in HNSCC patients (Figure 2(j)). Except to the genes SOCS1, NLRP1, GZMB, and CDKN2A, the other 17 FPRGs play a role as risk factors in the prognosis of HNSCC.

### 3.2. Unsupervised Clustering Based on 21 FPRGs

**Figure 3:**
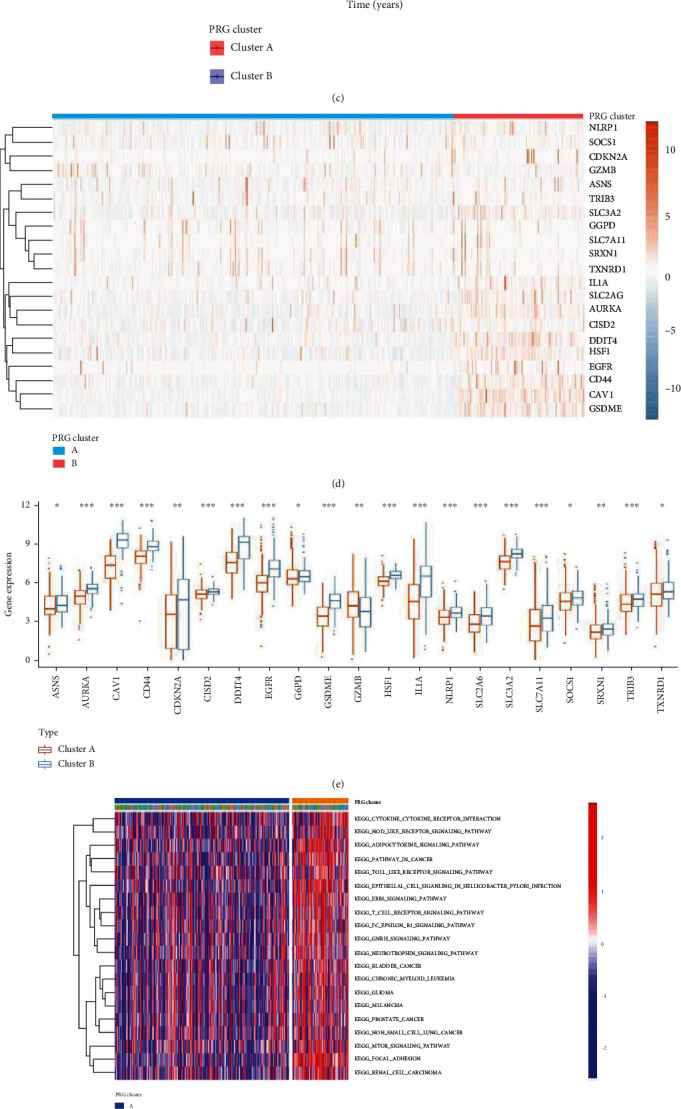
Unsupervised clustering based on 21 FPRGs. (a) The HNSCC patients were stratified into 2 clusters based on the 21 FPRGs using consensus clustering matrix (*k* = 2). (b) PCA plot of the cluster A and cluster B groups based on 21 FPRGs. (c) The K–M analysis of the overall survival in cluster A and cluster B groups. (d) Expression heat map of the 21 FPRGs between cluster A and cluster B groups. (e) Differential expression histogram of the 21 FPRGs between cluster A and cluster B groups. (f) GSVA enrichment analysis showing the activation states of biological pathways in cluster A and cluster B groups. The heat map was used to visualize these biological processes, and red represented activated pathways and green represented inhibited pathways. (g) GO functional enrichment analysis visualized with an enrichment circle diagram. FPRGs: ferroptosis and pyroptosis-related genes; HNSCC: Head and neck squamous cell carcinoma; PCA: principal component analysis; K-M: Kaplan–Meier.

The “ConsensusClusterPlus” R package was performed to cluster the HNSCC patients in TCGA-HNSC dataset into two different subtypes based on the expression of the 21 FPRGs (cluster A and cluster B, Figure 3(a)). PCA revealed that we can completely distinguished cluster A and cluster B based on the expression level of the 21 FPRGs (Figure 3(b)). K-M analysis for the two different subtypes revealed that the patients in cluster B group have poor outcome than the patients in cluster A group (Figure 3(c)). In addition to the gene GZMB, the other 20 FPRGs were higher expressed in cluster B group than in cluster A group (Figures 3(d) and 3(e)). To compare the different biological behaviors between the two subtypes, GSVA analysis was performed. As shown in Figure 3(f), we found that cluster B mainly enriched in pathways associated with malignant progression of cancer such as _MAPK_SIGNALING_PATHWAY, P53_SIGNALING_PATHWAY, and CHEMOKINE_SIGNALING_PATHWAY, which verifies the poor prognosis of cluster B patients (Figure 3(f)). To further explore the biological function of each subtype, we identified 165 prognostic DEGs through differential expression analysis (Supplementary Table 4) and univariate Cox regression analysis (Supplementary Table 5). GO enrichment analysis was performed based on the 165 prognostic DEGs using the “clusterProfiler” R package. The results indicated that the 165 prognostic DEGs mainly enriched in GO:0005198~structural molecule activity, GO:0005882~intermediate filament, GO:0005615~extracellular space, etc. (Figure 3(g), Supplementary table 6).

### 3.3. Construction and Validation of a Prognostic Risk Model

**Figure 4:**
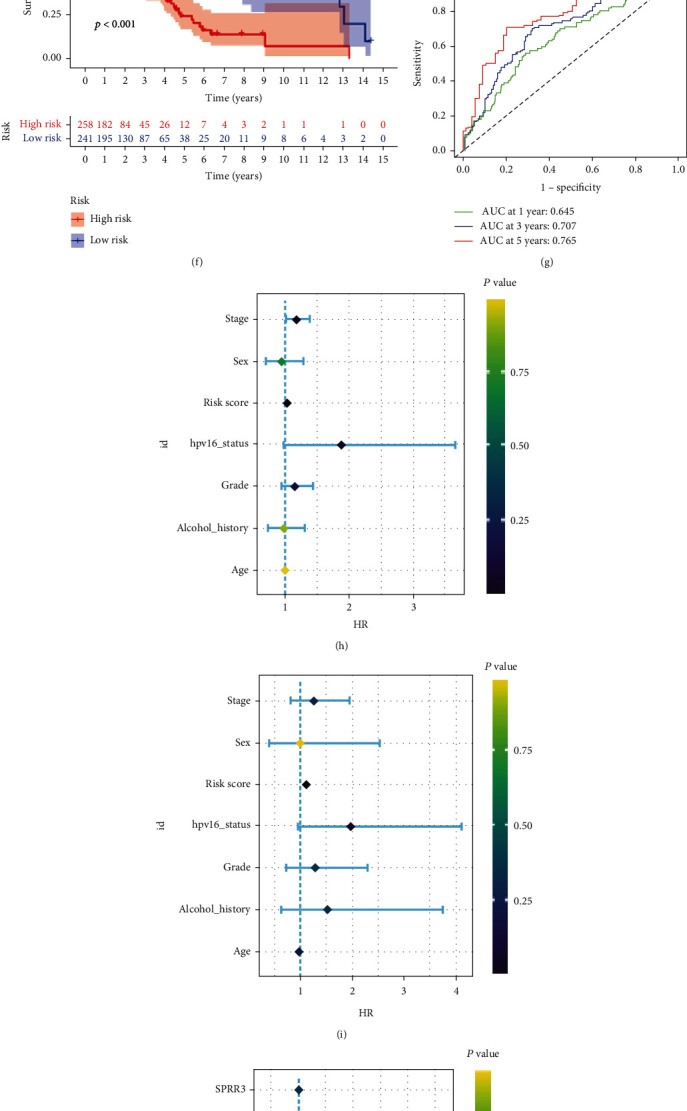
Establishing process of the prognostic model based on the TCGA-all dataset. (a) LASSO Cox regression analysis. (b) PCA plot of the high- and low-risk groups based on 9 FPRGs. (c–e) The heat map, risk score distribution, and survival status of patients. (f, g) The K-M survival curve and ROC curve for the risk score in predicting the OS of HNSCC patients. (h, i) Univariate and multivariate Cox analysis to assess the independence of the risk score. (j) The univariate Cox regression analysis based forest plot in 9 FPRGs. (k-r) The K-M survival analysis of the 9 FPRGs. LASSO: least absolute shrinkage and selection operator; PCA: principal component analysis; K-M: Kaplan–Meier; ROC: receiver operating characteristic.

**Figure 5:**
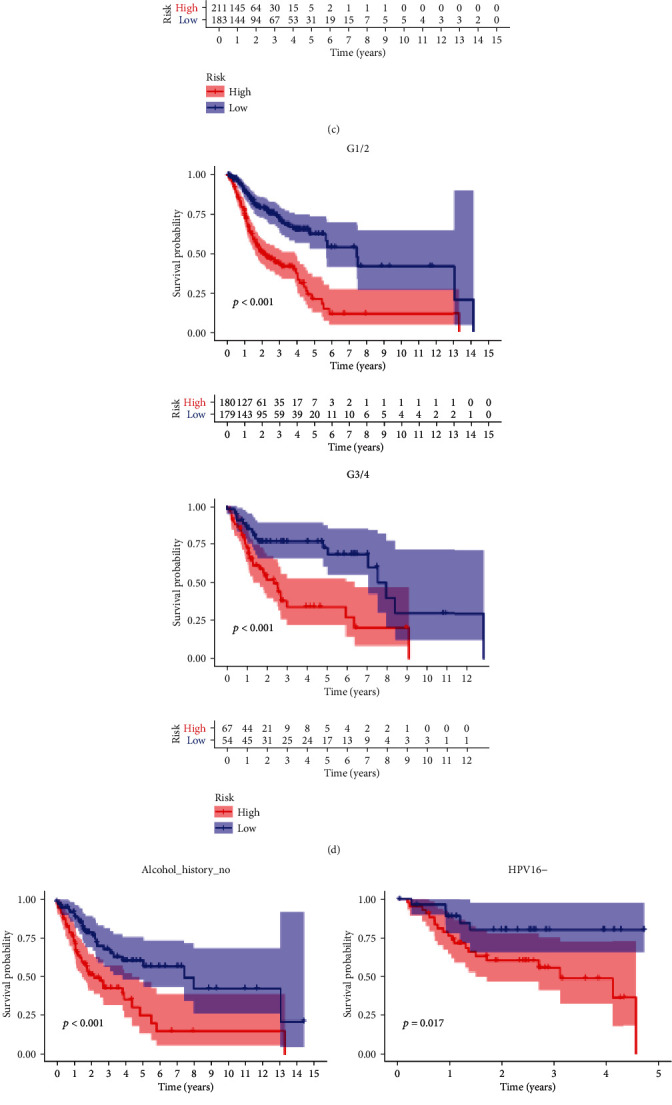
Stratified survival analysis in the TCGA-all dataset. (a) Age. (b) Gender. (C) TNM stage. (d) Histology grade. (e) Alcohol_history. (f) Hpv16_status. (g) New tumor event after initiative treatment. (h) Perineural_invasion.

The 165 prognostic DEGs was subjected to the LASSO regression analysis to avoid overfitting (Figure 4(a)), and a risk model with 9 prognostic DEGs was built by multivariate Cox regression analysis. Each patient in TCGA-HNSC dataset obtained a risk score according to the following formula: risk score =0.5145 × expAC006159.1 + 0.6966 × expAC117422.1 + 0.8599 × expAC128687.2 + 0.0032 × expAL161431.1 -0.2028 × expFCRL1 - 0.0106 × expLRATD1+0.1715 × exp PDCL2 - 0.0223 × exp PLA2G3+0.0002 × exp SPRR3. The HNSCC patients was clustered into high- and low-risk group based on the median cut-off of risk score (Figure 4(c)). PCA analysis revealed that the patients in high- and low-risk group can be separated completely based on the expression level of the 9 prognostic DEGs (Figure 4(b)). The patients in high-risk group had more deaths (Figure 4(d)). The heat maps which presented the expression level of AC117422.1, AC117422.1, AC128687.2, AL161431.1, PDCL2, and SPRR3 were increased with the increase of risk score, whereas the expression levels of FCRL1, LRATD1 and PLA2G3 were decreased (Figure 4(e)). K-M curve demonstrated that the patients in low-risk group have longer overall survival (OS) time than the patients in high-risk group (Figure 4(f)). ROC curve indicated that the prognostic predictive performance of the risk model was robustly and the AUC values at 1, 3, and 5 years were 0.645, 0.707, and 0.765, respectively (Figure 4(g)). The risk score was an independent prognostic predictor for OS according to the univariate and multivariate Cox regression analysis (Figures 4(h) and 4(i)). We also found that FCRL1, LRATD1, and PLA2G3 were prognostic protective factors for the OS of the HNSCC patients, whereas the AC117422.1, AC117422.1, AC128687.2, AL161431.1, PDCL2, and SPRR3 were prognostic risk factors according to the univariate Cox regression analysis and K-M analysis (Figures 4(j)–4(r)). Stratified prognostic analysis based on the clinical characteristics showed that patients in the high-risk group continued to have poor outcomes except for the HPV+ group (Figure 5).

**Figure 6:**
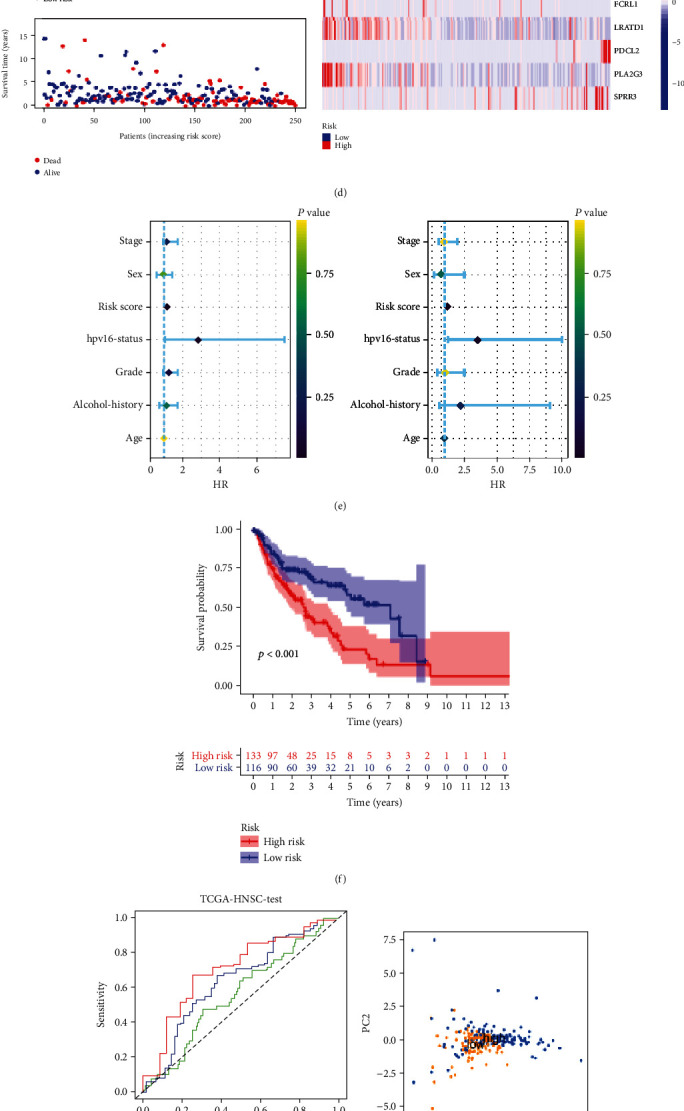
Internal validation of the prognostic model. (a, b) The K-M survival curve and ROC curve for the risk score in predicting the OS of HNSCC patients in the TCGA- training dataset. (c) PCA plot of the high-and low-risk groups based on 9 FPRGs in the TCGA- training dataset. (d) The heat map, risk score distribution, and survival status of patients in the TCGA-training dataset. (e) Univariate and multivariate Cox analysis to assess the independence of the risk score in the TCGA- training dataset. (f, g) The K-M survival curve and ROC curve for the risk score in predicting the OS of HNSCC patients in the TCGA-testing dataset. (h) PCA plot of the high-and low-risk groups based on 9 FPRGs in the TCGA- testing dataset. (i) The heat map, risk score distribution, and survival status of patients in the TCGA-testing dataset. (j) Univariate and multivariate Cox analysis to assess the independence of the risk score in the TCGA-testing dataset. HNSCC: head and neck squamous cell carcinoma; PCA: principal component analysis; K-M: Kaplan–Meier; ROC: receiver operating characteristic.

The HNSCC patients in TCGA-HNSC dataset were randomly divided into TCGA-training dataset and TCGA-testing dataset to confirm the performance of the risk model by internal validation. In the TCGA-training dataset, the patients in low-risk group have longer OS than in high-risk group (Figure 6(a)). The AUC values at 1-, 3-, and 5-year OS predicted by the risk model were 0.741, 0.768, and 0.811, respectively (Figure 6(b)). PCA analysis indicated that the expression level of the 9 prognostic DEGs can separate the patients in high- risk group from the low-risk group completely (Figure 6(c)). The risk score, survival state, and heat map of the 9 prognostic DEGs in the TCGA-training dataset are presented in Figure 6(d). Univariate and multivariate Cox regression analysis indicated that the risk score was an independent prognostic predictor for OS (Figure 6(e)). In the TCGA-testing dataset, the OS in the high-risk group was shorter than the low-risk group (Figure 6(f)). The AUC values at 1-, 3-, and 5-year OS predicted by the risk model were 0.562, 0.638, and 0.707, respectively (Figure 6(g)). The high-risk group can be separated from the low-risk group based on the 9 prognostic DEGs (Figure 6(h)). Figure 6(i) showed the risk score, survival state, and heat map of the 9 prognostic DEGs in the TCGA-testing dataset. The risk score was an independent prognostic predictor for OS, as revealed by univariate and multivariate Cox regression analysis (Figure 6(j)).

**Figure 7:**
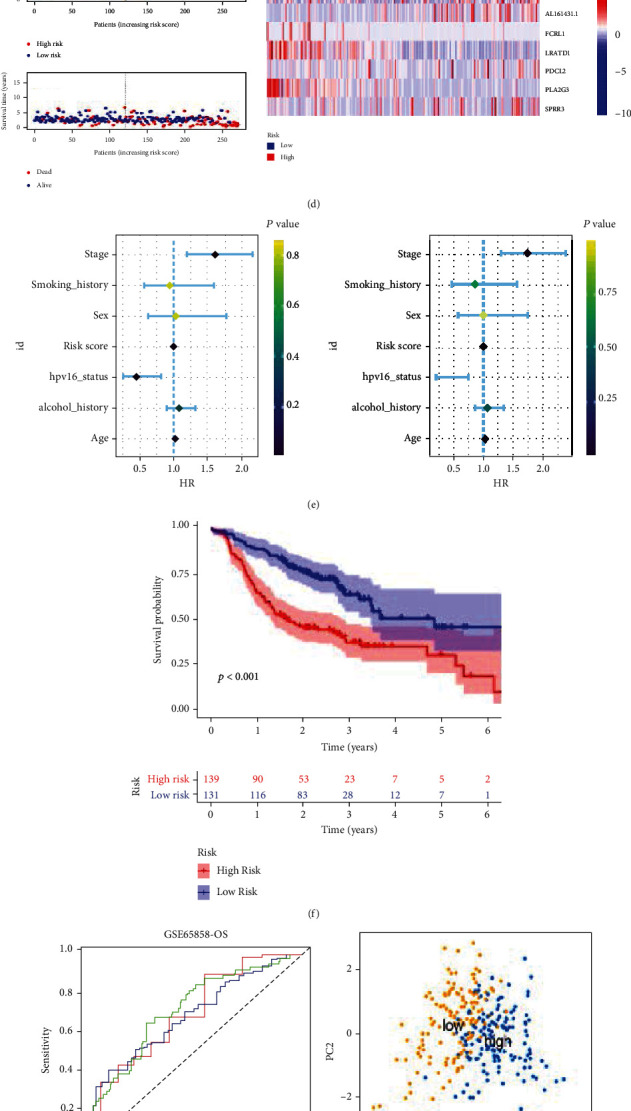
External validation of the prognostic model in the GSE65858 dataset. (a, b) The K-M survival curve and ROC curve for the risk score in predicting the OS of HNSCC patients in the GSE65858-OS dataset. (c) PCA plot of the high- and low-risk groups based on 9 FPRGs in the GSE65858-OS dataset. (d) The heat map, risk score distribution, and survival status of patients in the GSE65858-OS dataset. (e) Univariate and multivariate Cox analysis to assess the independence of the risk score in the GSE65858-OS dataset. (f, g) The K-M survival curve and ROC curve for the risk score in predicting the OS of HNSCC patients in the GSE65858-PFS dataset. (h) PCA plot of the high-and low-risk groups based on 9 FPRGs in in the GSE65858-PFS dataset. (i) The heat map, risk score distribution, and survival status of patients in the GSE65858-PFS dataset. (j) Univariate and multivariate Cox analysis to assess the independence of the risk score in the GSE65858-PFS dataset. HNSCC: head and neck squamous cell carcinoma; PCA: principal component analysis; K-M: Kaplan–Meier; ROC: receiver operating characteristic.

The HNSCC patients in GSE65858 dataset was used to confirm the performance of the risk model by external validation. The OS and relapse-free survival (RFS) in the low-risk group was longer than in the high-risk group (Figures 7(a) and 7(f)). The AUC values at 1-, 3-, and 5-year OS predicted by the risk model were 0.783, 0.725, and 0.675, respectively (Figure 7(b)). The AUC values at 1-, 3-, and 5-year RFS predicted by the risk model were 0.719, 0.687, and 0.695, respectively (Figure 7(g)). The patients in high-risk group can be distinguished from the low-risk group based on the 9 prognostic DEGs (Figures 7(c) and 7(h)). Figures 7(d) and 7(i) showed the risk score, survival state and heat map of the 9 prognostic DEGs. Figures 7(e) and 7(j) demonstrated that the risk score was an independent prognostic indicator for OS and RFS.

**Figure 8:**
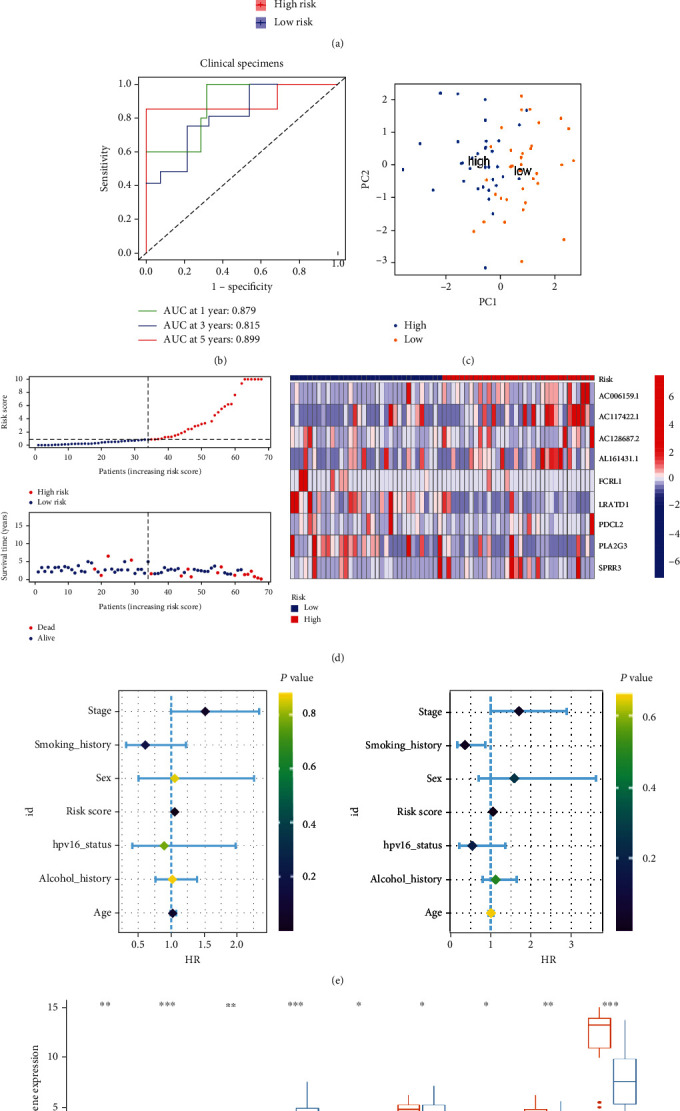
External validation of the prognostic model in the clinical specimens. (a, b) The K-M survival curve and ROC curve for the risk score in predicting the OS of HNSCC patients. (c) PCA plot of the high- and low-risk groups based on 9 FPRGs. (d) The heat map, risk score distribution, and survival status of patients. (e) Univariate and multivariate Cox analysis to assess the independence of the risk score. (f) Differential expression histogram of the 9 FPRGs between HNSCC and normal samples. HNSCC: head and neck squamous cell carcinoma; PCA: principal component analysis; K-M: Kaplan–Meier; ROC: receiver operating characteristic.

The qRT-PCR was operated to examine the expression of the 9 prognostic DEGs in 40 healthy samples and 68 HNSCC samples. We then built a risk model based on the 9 prognostic DEGs using the same methods mentioned in TCGA-HNSC dataset. We successfully verified the favorable prognostic predictive performance of the risk model according to the Figures 8(a)–8(e). We also compared the differential expression of the 9 prognostic DEGs between healthy samples and HNSCC samples. The results suggested that AC117422.1, AC117422.1, AC128687.2, AL161431.1, and FCRL1 were elevated and LRADT1, PDCL2, PLA2G3, and SPRR3 were declined in the HNSCC samples (Figure 8(f)). Finally, univariate and multivariate Cox analysis indicated that the risk score was the independent factor for the prognosis of the HNSCC patients (Table 2).

### 3.4. Construction of a Nomogram Model

**Figure 9:**
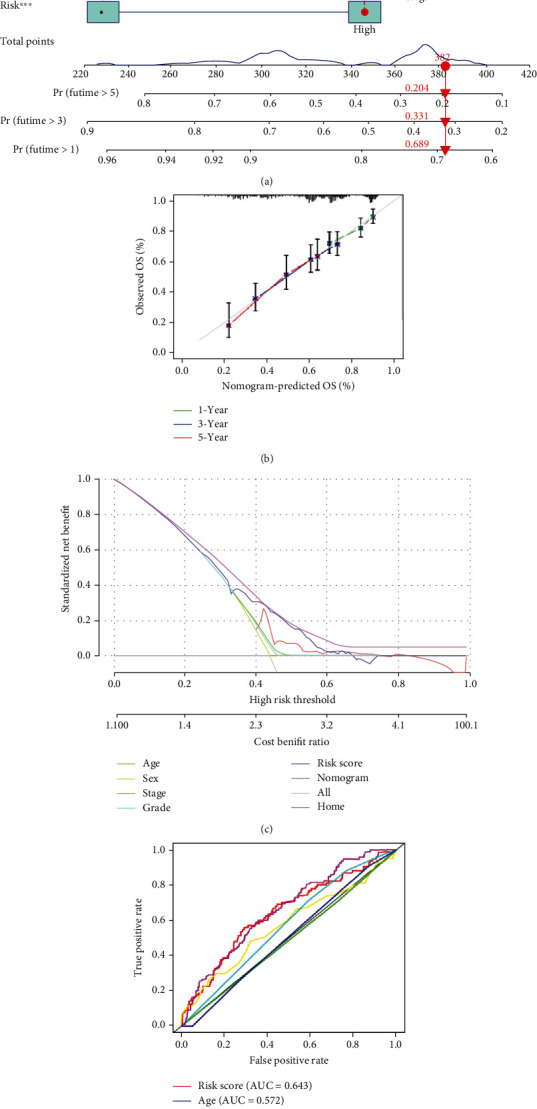
Establishing process of the nomogram model. (a) Nomogram for predicting the 1-, 3-, and 5-year overall survival of HNSCC patients in the TCGA-dataset. (b) Calibration curves of the nomogram model of 1, 3, and 5 years. (c) DCA curves for predicting the overall survival of different parameters. (d) ROC curves for predicting the overall survival of different parameters. HNSCC: head and neck squamous cell carcinoma; DCA: clinical decision curve; ROC: receiver operating characteristic.

To improve the clinical applicability, a nomogram model combined risk score and clinical information was constructed (Figure 9(a)). Calibration curve at 1-, 3-, and 5-year indicated that the predicted OS probability of the nomogram model was close to the actual (Figure 9(b)). DCA curve suggested that the nomogram model has the highest net benefit compared with the individual features (Figure 9(c)). ROC curve indicated that the nomogram model has the optimum sensitivity and specificity in prognostic prediction than the individual features (Figure 9(d)).

### 3.5. Exploration of the Clinicopathological Features and Stemness Characteristics of the Prognostic Risk Model

**Figure 10:**
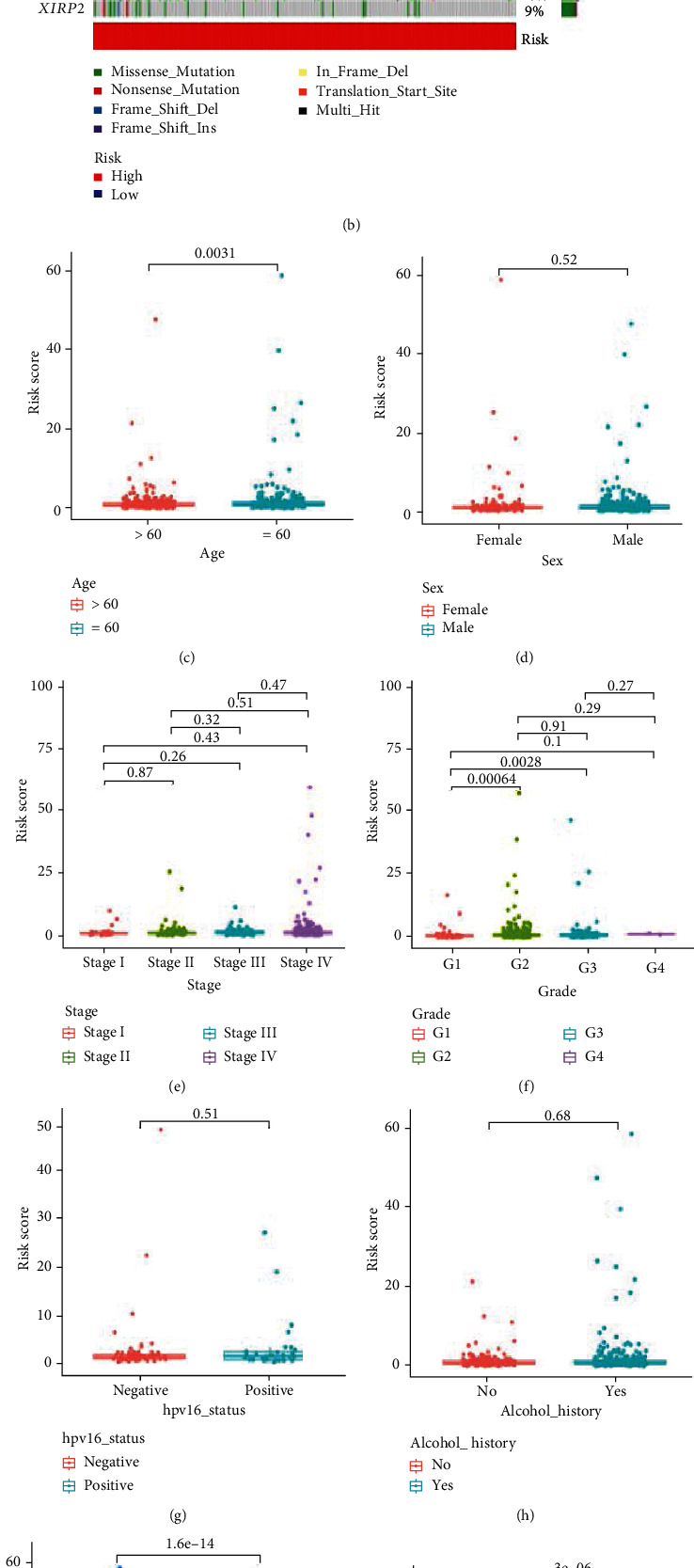
Comprehensive analysis of the risk model in HNSCC. (a, b) The waterfall plot of somatic mutation features established with high- and low-risk group. (c-j) Risk scores of the HNSCC patients are classified by age, gender, stage, histology grade, alcohol_history, Hpv16_status, cluster, and TMB. (k) Relationships between risk score and TMB. (l) Sankey diagram shows the relationships among cluster, risk score, and survival status. HNSCC: head and neck squamous cell carcinoma; TMB: tumor mutation burden.

The mutation frequency of the *TP53* associated with adverse outcome of cancer was found higher in high-risk group (67%, Figure 10(a)), compared with the low-risk group (58%, Figure 10(b)). To investigate the relationship between risk score and clinicopathological features, the “compare” R package was performed. We observed that the patients with age ≤60 had higher risk score than the patients with age>60, whereas the risk score has no statistic difference in other stratified clinicopathological features (Figures 10(c)–10(h)). The patients in cluster A group corresponds to higher risk score (Figure 10(i)) and the relationship of cluster, risk, and fustat was showed in Sankey diagram (Figure 10(l)). Increasing evidence revealed that the patients with higher TMB can more benefit from immunotherapy [28]. Figures 10(j) and 10(k) showed that the risk score has negative correlation with TMB, suggesting that the patients in low-risk group were more sensitive to immunotherapy.

### 3.6. Tumor Immune Characteristics Analysis

**Figure 11:**
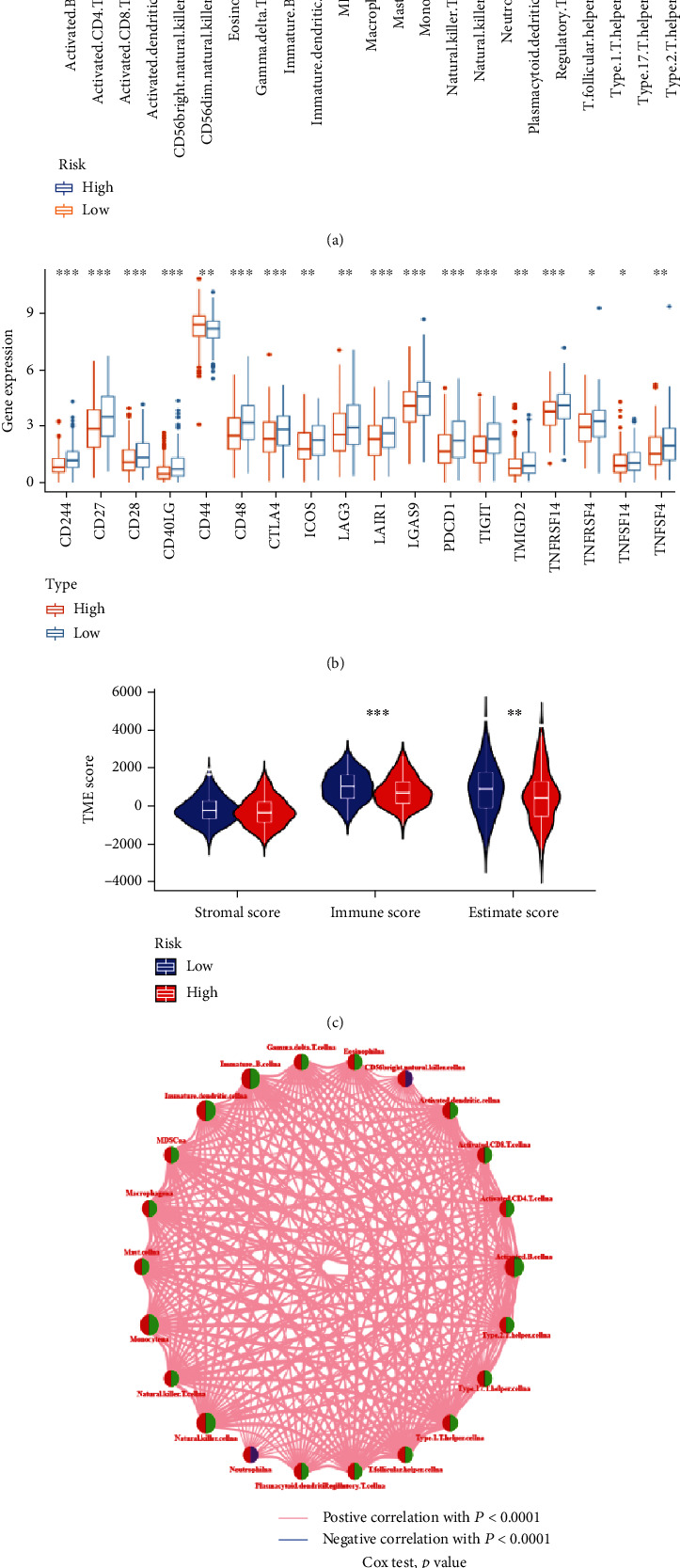
Comprehensive analysis of the TME and checkpoints between high- and low-risk groups. (a) Differential expression histogram of the 23 types of immune cells between high- and low-risk groups. (b) Differential expression histogram of the immune checkpoints between high- and low-risk groups. (c) Correlations between risk score and both immune and stromal scores. (d) The correlation network among the 23 types of immune cells.

**Figure 12:**
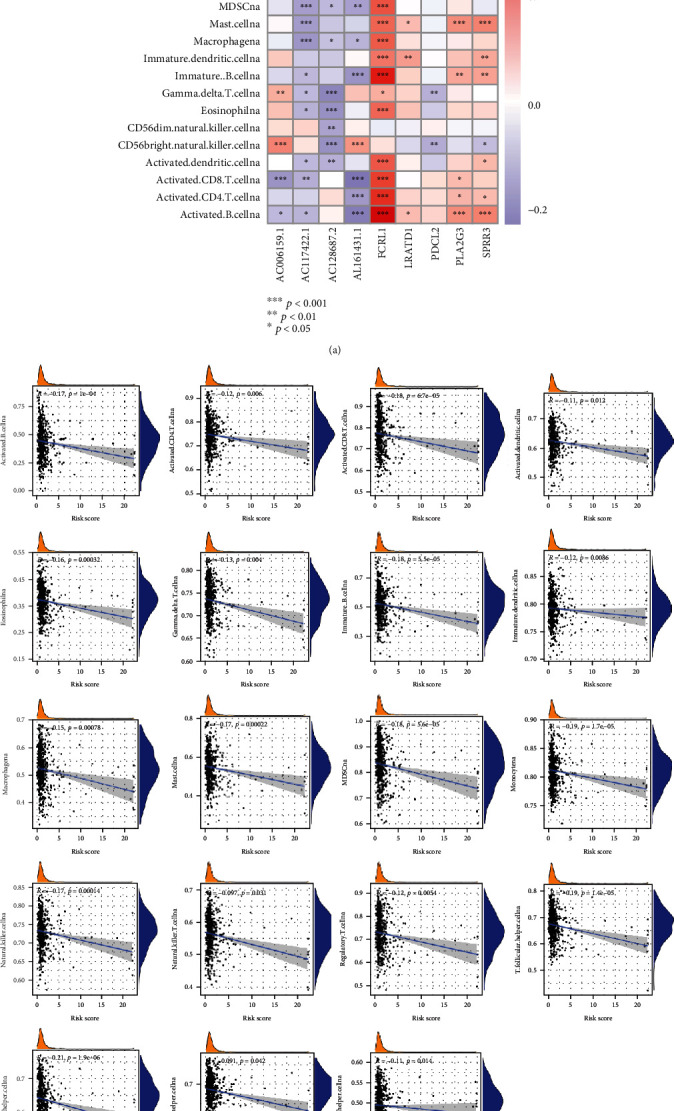
Evaluation of the TME between high- and low-risk groups. (a) Correlations between the 23 types of immune cells and the 9 FPRGs in the risk model. (b) Correlations between risk score and 23 types of immune cells. TME: tumor microenvironment.

The abundance of immune cells was calculated using the ssGSEA algorithm, and the different levels of immune cell infiltration between high- and low-risk group was compared, finding that the HNSCC samples in low-risk group has increased immune response (Figure 11(a)). We also investigated the differential expression levels of immune checkpoints, finding that there were 19 immune checkpoints overexpressed in the low-risk group than that in the high-risk group (Figure 11(b)). In addition, the HNSCC samples in low-risk group were related to higher immune score and ESTIMATEScore (Figure 11(c)). The network presented the interactions, regulator connection, and prognostic value of the 23 types of immune cells (Figure 11(d)). We found that there were strong positive correlation and mutual regulation between the 22 types of immune cells (except CD56dim natural killer cells, Supplementary table 7). Combined with the network graph and K-M curve (Supplement Figure 1), 17 types of immune cells were associated with the prognosis of the HNSCC patients. Among them, 16 types of immune cells were protective factors, and neutrophilia was a risk factor. Spearman correlation analysis was performed to evaluate the correlation between the 9 prognostic DEGs in risk model and the 23 types of immune cells. We observed that FCRL1 has the strongest positive correlation with the 23 types of immune cells, while the AC128687.2 has the strongest negative correlation with the 23 types of immune cells (Figure 12(a)).  Figure 12(b) revealed that the infiltration abundance of 17 immune cells was reduced as the risk score.

## 4. Discussion

Head and neck tumors commonly occur in the oral cavity, nasopharynx, oropharynx, hypopharynx, and larynx [29]. As the most common pathologic type, HNSCC ranks 6th in the incidence rate of malignant tumors worldwide, and more than 800,000 new cases are diagnosed every year [30]. At present, surgery is the main treatment, supplemented by radiotherapy and chemotherapy, but the 5-year survival rate is still not ideal [31]. Especially, local recurrence and distal organ metastasis often occur in advanced HNSCC after treatment, with higher mortality [32]. Targeted therapy and immunotherapy are promising for patients with advanced HNSCC. Currently, cetuximab is the only molecular-targeted drug approved for clinical treatment of HNSCC. Combined with platinum-based chemotherapy, cetuximab is the standard treatment for patients with advanced HNSCC [33, 34]. Nivolumab and pembrolizumab are two immunocheckpoint inhibitors permitted for immunotherapy in HNSCC patients [33, 35]. However, drug resistance and immune escape are major problems faced by targeted and immunotherapy. Induction of apoptosis to halt tumor growth is the aim of many HNSCC treatment strategies [13]. Ferroptosis and pyroptosis are two new PCD modes discovered in recent years and play important roles in the malignant processes and immune microenvironment of HNSCC. Therefore, target ferroptosis and pyroptosis- related genes may be improved the prognosis of HNSCC.

In this study, we successfully constructed and verified a risk model based on the FPRGs. Firstly, we obtained 21 significant FPRGs differential expression analysis and univariate Cox regression analysis. The patients in TCGA-HNSC dataset were divided into two different subtypes based on the expression of the 21 FPRGs using the “ConsensusClusterPlus” R package. Further, we acquired the 165 prognostic DEGs between the two subtypes to explore the molecular differences of the two subtypes. GO enrichment analysis indicated that the 165 prognostic DEGs mainly enriched in GO:0005198~structural molecule activity, GO:0005882~intermediate filament, GO:0005615~extracellular space, etc. These extracellular components were an important part of the immune microenvironment [36]. Ferroptosis and pyroptosis have been widely reported to have extremely complicated crosstalk with tumor immune microenvironment [5]. The 165 DEGs with prognostic significance was subjected into the LASSO-multivariate Cox regression analysis to a built a risk model with 9 prognostic DEGs. K-M curve revealed that the patients with higher risk score have poor outcome compared the patients with lower risk score. ROC curve indicated that the sensitivity and specificity of the risk score for prognostic prediction in HNSCC patients was favorable. More importantly, the risk model was successfully verified with a stable prognostic value through internal validation, external validation, and clinical sample validation. In addition, the risk score was found to be independent of other clinical information in predicting the prognosis of HNSCC. Regardless of the clinical characteristics, stratified prognostic analysis showed that the HNSCC patients in the high-risk group continued to have poor outcomes except for the HPV+ group. This result may be due to the small sample size of HPV+ group (*n* = 31). Finally, a nomogram model combined risk score and clinical information was constructed to improve the clinical applicability. Reviewing previous studies, some of these 9 prognostic DEGs have been found to be involved in the occurrence and progression of solid tumors. For example, Lu Yu et al. [37] reported that the expression level of SPRR3 was reduced as the malignant progression of oral squamous cell carcinoma (OSCC), which was consistent with our analysis results. AL161431.1 was found upregulated in endometrial carcinoma, lung cancer, and pancreatic cancer and associated with the immune microenvironment, proliferation, migration, epithelial-mesenchymal transformation, and poor prognosis [38, 39]. Randall S. Davis [40] identified that FCRL1 overexpressed in breast, melanoma, and lung cancer may be a potential biomarker and therapeutic target. LRATD1 also named FAM84A has been revealed to be related to the occurrence and development of papillary thyroid cancer, liver tumor, and colon cancer [41–43]. PLA2G3 was upregulated in ovarian cancer, melanoma, and colorectal cancer and improved the poor prognosis and malignant progression of cancer [44–46].

TP53 is one of the most frequently altered genes in human cancers, which is present in about 50% of invasive tumors [47]. Genomic data showed that TP53 was the most common mutant gene in HNSCC and associated with shorter survival outcome of HNSCC patients [48]. Our research found that the patients in high-risk group with poor survival outcome have higher TP53 mutation frequency (67%) than the patients in low-risk group (58%), which was consistent with the results of previous studies. TMB refers to the number of nonsynonymous mutations in somatic cells per mega base pair (Mb) in a specific genomic region, which can indirectly reflect the ability of tumor to produce neoantigens [49]. Patients with higher TMB are more likely to benefit from immunotherapy [49]. In this study, we found that the risk score has negative correlation with TMB, suggesting that the patients in low-risk group were more sensitive to immunotherapy.

To reveal the mechanism of risk model in tumor immune microenvironment, we firstly calculated the abundance of immune cells using the ssGSEA algorithm and compared the differential immune cell infiltration among the high- and low-risk group. The results identified that Activated.B.cellna, Activated.CD8.T.cellna, Eosinophilna, Immature.B.cellna, MDSCna, Macrophagena, Mast.cellna, Monocytena, Natural.killer.cellna, T.follicular.helper.cellna, Type.1.T.helper.cellna, and Type.17.T.helper.cellna had decreased infiltration as the risk score increased. K-M curve revealed that the above 12 type of immune cells except T.follicular.helper.cellna are the protective prognostic factor for HNSCC. B cells are the main effector cells of humoral immunity, which can directly kill tumor cells and inhibit tumor development by secreting immunoglobulin [50]. Xin Feng et al. showed that the B cells act a favorable role in the prognosis of HNSCC. The higher infiltration of B cell and their subtypes may improve the prognosis of HPV+ HNSCC patients [51]. Activated CD8 T cells as the most important antitumor effector cells can recognize tumor associated antigens by expressing T cell receptors and kill tumor cells [52]. Many studies have shown that the HNSCC patients can benefit from the increased infiltration of the activated CD8 T cells [53]. Eosinophils can kill tumors directly or indirectly by releasing cytotoxic proteins or chemoattractants, which may extend the prognosis of HNSCC patients [54]. As the first line of defense against tumor, natural killer cells have been reported to play an important role in antitumor immunity of HNSCC [54]. T helper cells, as the most important helper cells in tumor immunity, can promote the recruitment of natural killer cells to the tumor and activate death receptors on the surface of tumor cells and the CD8 T cells by releasing cytokines [21]. Contrary to our analysis, monocytes, macrophages, mast cells, and myeloid derived suppressor cells (MDSCs) were considered to relate to the malignant progression and poor prognosis of HNSCC [55–58].

## 5. Conclusions

Our research identified a favorable risk model containing 9 FPRGs, which could be potential prognostic markers and effective immunotherapy targets for HNSCC.

## Figures and Tables

**Table 1:** The clinical information of the patient in TCGA-HNSC dataset, GSE65858 dataset and clinical specimens.

Characteristic	TCGA-HNSC (502)	GSE65858 (270)	Clinical specimens (68)
Age(years)			
≤ 60	245	158	38
> 60	256	112	30
None	1	0	0
Sex			
Female	134	47	7
Male	368	223	61
None	0	0	0
OS_Event			
Alive	283	176	51
Dead	218	94	17
None	1	0	0
PFS_Event			
FALSE		137	
TRUE		133	
None		0	
Grade			
1	62		
2	300		
3	119		
4	2		
None	19		
Stage			
I	25	18	6
II	81	37	12
III	90	37	6
IV	306	178	44
None	0	0	0
Alcohol_history			
Yes	333	239	59
No	158	31	9
None	11	0	0
Hpv16_status			
Positive	31	60	11
Negative	72	209	57
None	399	1	0
Perineural_invasion			
Yes	165		
No	188		
None	149		
New tumor event after initiative treatment			
Tumor free	275		
With tumor	107		
None	120		
Smoking_history			
Yes		222	53
No		48	15
None		0	0

**Table 2:** Univariate and multivariate Cox analysis to assess the independence of the risk score.

Variables	Univariate analysis			Multivariate analysis		
	*P*	HR	95% CI	*P*	HR	95% CI
TCGA-HNSC-all						
Age	0.9966	1.0000	0.9878-1.0121	0.2351	0.9804	0.9488-1.0129
Sex	0.7151	0.9454	0.6991-1.2782	0.9880	0.9928	0.3894-2.5311
Stage	0.0312	1.1840	1.0153-1.3805	0.2991	1.2603	0.8143-1.9504
Grade	0.1654	1.1592	0.9408-1.4281	0.3882	1.2888	0.7242-2.29345
Alcohol_history	0.9108	0.9839	0.7399-1.3081	0.3565	1.5259	0.6214-3.7465
Hpv16_status	0.0613	1.8848	0.9704-3.6608	0.0675	1.9798	0.9519-4.1174
Risk score	0.0002	1.0310	1.01465-1.0755	0.0031	1.1110	1.04-1.185
TCGA-HNSC-train						
Age	0.9649	1.0004	0.9827-1.0183	0.3963	0.9706	0.9058-1.0398
Sex	0.7824	0.9400	0.6058-1.4583	0.5445	0.6641	0.1767-2.4956
Stage	0.2480	1.1429	0.9111-1.4334	0.9922	1.0032	0.5265-1.9112
Grade	0.1152	1.2773	0.9419-1.7320	0.9626	1.0222	0.4084-2.5582
Alcohol_history	0.5488	1.1364	0.7482-1.7257	0.2686	2.2157	0.5413-9.0695
Hpv16_status	0.0357	2.8341	1.0719-7.4930	0.0184	3.5167	1.2364-10.0017
Risk score	0.0000	1.1546	1.1139-1.1966	0.0107	1.1605	1.0351-1.3010
TCGA-HNSC-test						
Age	0.9848	0.9998	0.9832-1.0167	0.4098	0.9819	0.9401-1.0254
Sex	0.5866	0.8898	0.5842-1.3552	0.6305	1.4541	0.3162-6.6852
Stage	0.0589	1.2252	0.9924-1.5126	0.1725	1.7161	0.7899-3.7281
Grade	0.9604	1.0075	0.7514-1.3506	0.3151	1.5884	0.6439-3.9177
Alcohol_history	0.3601	0.8328	0.5628-1.2322	0.4273	1.6790	0.4670-6.0353
Hpv16_status	0.6570	1.2422	0.4769-3.2352	0.8114	0.8586	0.2454-3.0034
Risk score	0.0001	1.0076	1.0036-1.0115	0.0045	1.2171	1.0627-1.3938
GSE65858-OS						
Alcohol_history	0.4138	1.0850	0.8922-1.3192	0.5154	1.0748	0.8647-1.3359
Stage	0.0014	1.6154	1.2039-2.1675	0.0003	1.7431	1.2855-2.3633
Hpv16_status	0.0093	0.4567	0.2529-0.8246	0.0036	0.3920	0.2088-0.7358
Smoking_history	0.8211	0.9409	0.5549-1.5953	0.6200	0.8600	0.4736-1.5611
Sex	0.8683	1.0456	0.6174-1.7705	0.9977	1.0008	0.5738-1.7454
Age	0.0126	1.0266	1.0056-1.0479	0.0123	1.0296	1.0063-1.0533
Risk score	0.0000	1.0055	1.0036-1.0073	0.0000	1.0049	1.0029-1.0068
GSE65858-PFS						
Alcohol_history	0.0287	1.2055	1.0196-1.4250	0.0711	1.1893	0.9851-1.4356
Stage	0.0502	1.2255	0.9998-1.5021	0.0463	1.2350	1.0034-1.5199
Hpv16_status	0.0101	0.5460	0.3443-0.8657	0.0209	0.5629	0.3457-0.9164
Smoking_history	0.7890	1.0646	0.6731-1.6835	0.4950	0.8370	0.5023-1.3954
Sex	0.2860	1.2897	0.80816-2.0581	0.5836	1.1459	0.7041-1.8647
Age	0.2760	1.0095	0.9924-1.0269	0.1240	1.0145	0.9960-1.0333
Risk score	0.0000	1.1503	1.1025-1.2001	0.0000	1.1292	1.0808-1.17983
Clinical specimens						
Alcohol_history	0.8873	1.0219	0.7570-1.3794	0.4936	1.1385	0.7852-1.6506
Stage	0.0524	1.5243	0.9957-2.3334	0.0485	1.7069	1.00352.9031
Hpv16_status	0.7925	0.8999	0.4099-1.9751	0.1896	0.5390	0.2140-1.3572
Smoking_history	0.1699	0.6207	0.3141-1.2265	0.0221	0.3706	0.1583-0.8671
Sex	0.8696	1.0648	0.5031-2.2532	0.2728	1.5868	0.6952-3.6218
Age	0.1003	1.0279	0.9947-1.0620	0.6625	1.0086	0.9704-1.0482
Risk score	0.0000	1.0565	1.0373-1.0759	0.0000	1.0683	1.0453-1.0918

## Data Availability

All data generated or analyzed during this study are included in this published article and its supplementary information files.

## Supplementary Materials

Supplementary 1 Supplementary Figure 1. The K-M survival analysis of the 23 types of immune cells in the overall survival of HNSCC patients.

Supplementary 2 Supplementary Table 1. Primer sequences of the nine genes for reverse transcription- quantitative polymerase chain reaction.

Supplementary 3 Supplementary Table 2. Ninety-one DE ferroptosis and pyroptosis-related genes between normal and HNSCC samples were obtained through differential expression analysis in TCGA-HNSC dataset.

Supplementary 4 Supplementary Table 3. Forty-eight prognostic-related ferroptosis and pyroptosis were obtained by univariate Cox regression analysis in TCGA-HNSC dataset.

Supplementary 5 Supplementary Table 4. One thousand four hundred and sixty-seven DEGs between cluster A and cluster B were obtained through differential expression analysis in TCGA-HNSC dataset.

Supplementary 6 Supplementary Table 5. One hundred and sixty-five prognostic DEGs were obtained by univariate Cox regression analysis in TCGA-HNSC dataset.

Supplementary 7 Supplementary Table 6. GO enrichment analysis based on the 165 prognostic DEGs using the “clusterProfiler” R package.

Supplementary 8 Supplementary Table 7. Strong positive correlation and mutual regulation between the 22 types of immune cells (except CD56dim.natural.killer.cellna).
