# Supplementary material for: Relationships of Ferroptosis and Pyroptosis-Related Genes with Clinical Prognosis and Tumor Immune Microenvironment in Head and Neck Squamous Cell Carcinoma
Source: Oxid Med Cell Longev. 2022 Oct 5;2022:3713929. doi: 10.1155/2022/3713929 (PMC9557253; doi:10.1155/2022/3713929)
Supplement: Supplementary 1 — Supplementary Figure 1. The K-M survival analysis of the 23 types of immune cells in the overall survival of HNSCC patients. [file 3713929.f1.docx]

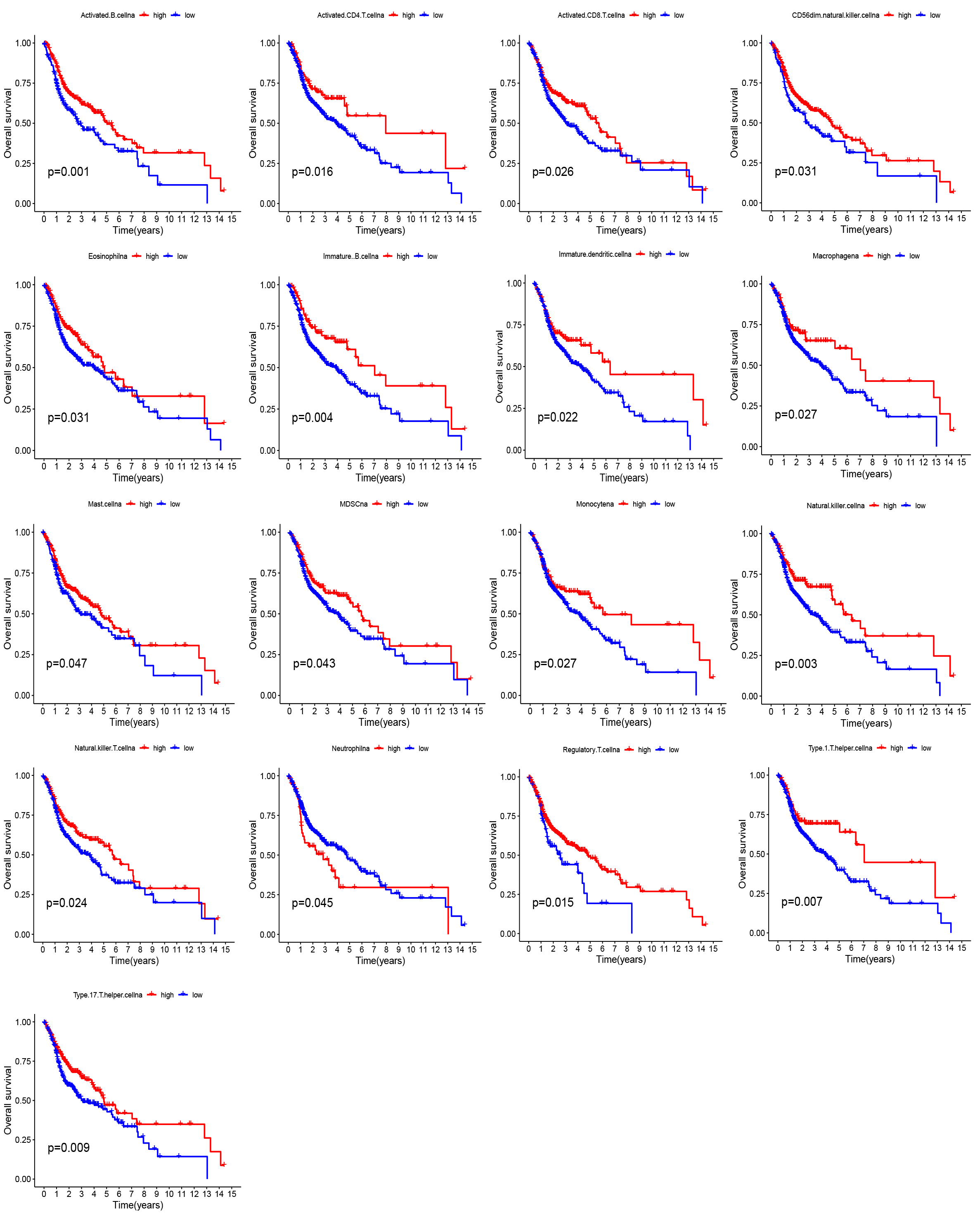


**Supplementary Figure 1.** The K-M survival analysis of the 23 types of immune cells in the overall survival of HNSCC patients.
