# Supplementary material for: Relationships of Ferroptosis and Pyroptosis-Related Genes with Clinical Prognosis and Tumor Immune Microenvironment in Head and Neck Squamous Cell Carcinoma
Source: Oxid Med Cell Longev. 2022 Oct 5;2022:3713929. doi: 10.1155/2022/3713929 (PMC9557253; doi:10.1155/2022/3713929)
Supplement: Supplementary 2 — Supplementary Table 1. Primer sequences of the nine genes for reverse transcription- quantitative polymerase chain reaction. [file 3713929.f2.docx]

Supplementary table 1: Primer sequences of the nine genes for reverse transcription- quantitative polymerase chain reaction.

| **Gene** | **Forward (5′-3′)** | Reverse (5′-3′) |
| --- | --- | --- |
| AC006159.1 | 5'-GTTGTGAGTTGCCCTGTGGAGAG-3' | 5'-CCCCTTTTCCCACCTGAGTTCATG-3' |
| AC117422.1 | 5'-CAAAGTGGAAGAGACGCTGTGGTAG-3'， | 5'-GTGAGAAGAACCGCCTGCTCTG-3'; |
| AC128687.2 | 5'-CGCCAGCCTTCATTCAGACTCC-3' | 5'-AGAAAGCCTGCGGTAGTGAAACG-3' |
| AL161431.1 | 5'-CGCTGGAAGGACTATGCTGAACC-3' | 5'-CGTGGGAACCTAGAGTGGGAGAG-3' |
| FCRL1 | 5'-GAGGACAGGTGATGGAGGGAGAC-3' | 5'-CCAGTACAGGATCGGAGGAGAGC-3' |
| LRATD1 | 5'-GGAAGGGGCTGAGGGGAGAAAG-3' | 5'-GGAGGCAAACACTGTCTGGCTTAG-3' |
| PDCL2 | 5'-CTCTTCTGGCGGTCACTCGAAAC-3' | 5'-AGCTTCCTTTAGCTGTGCAAGAGTC-3' |
| PLA2G3 | 5'-CTGTGGTGTGGAGTTGGAGATTCTG-3' | 5'-GATGGAGTCGTGCTGATTCTGTAGG-3' |
| SPRR3 | 5'-CCTTGAGAAGCCAACCACCAGATG-3' | 5'-GATGAACCCTGAGCAGCCGAAG-3' |
| GAPDH | 5'-TGACAACTTTGGTATCGTGGAAGG-3' | 5'-AGGCAGGGATGATGTTCTGGAGAG-3' |
