# Supplementary material for: Relationships of Ferroptosis and Pyroptosis-Related Genes with Clinical Prognosis and Tumor Immune Microenvironment in Head and Neck Squamous Cell Carcinoma
Source: Oxid Med Cell Longev. 2022 Oct 5;2022:3713929. doi: 10.1155/2022/3713929 (PMC9557253; doi:10.1155/2022/3713929)
Supplement: Supplementary 3 — Supplementary Table 2. Ninety-one DE ferroptosis and pyroptosis-related genes between normal and HNSCC samples were obtained through differential expression analysis in TCGA-HNSC dataset. [file 3713929.f3.docx]

Supplementary table 2. Ninety-one DE ferroptosis and pyroptosis related genes between normal and HNSCC samples was obtained through differential expression analysis in TCGA-HNSC dataset.

| gene | conMean | treatMean | logFC | pValue |
| --- | --- | --- | --- | --- |
| ABCC1 | 32.80205 | 85.29508 | 1.378677 | 3.30E-10 |
| AIM2 | 2.616135 | 36.27297 | 3.793386 | 2.44E-21 |
| ALOX12 | 49.78266 | 13.34445 | -1.8994 | 2.41E-06 |
| ALOX15 | 23.1253 | 4.921873 | -2.23219 | 0.000645 |
| ANGPTL7 | 4.830711 | 0.665979 | -2.85869 | 5.42E-12 |
| ARNTL | 3.514276 | 7.189005 | 1.032564 | 2.08E-11 |
| ASNS | 9.050751 | 26.7645 | 1.564211 | 7.91E-15 |
| AURKA | 8.158284 | 37.58948 | 2.203991 | 2.60E-24 |
| BAK1 | 41.44046 | 96.39363 | 1.217898 | 3.07E-16 |
| BAX | 34.16509 | 71.8878 | 1.073224 | 7.09E-20 |
| BID | 10.95663 | 29.38014 | 1.423037 | 6.19E-18 |
| CA9 | 1.037594 | 71.62293 | 6.109107 | 2.12E-24 |
| CASP5 | 0.408515 | 1.00739 | 1.30216 | 4.00E-10 |
| CASP8 | 6.004448 | 12.0481 | 1.004703 | 1.23E-13 |
| CAV1 | 66.71911 | 319.9462 | 2.261657 | 5.28E-14 |
| CBS | 0.046098 | 0.361616 | 2.971683 | 4.55E-08 |
| CD44 | 164.5806 | 339.6965 | 1.045451 | 1.51E-12 |
| CDKN2A | 2.843135 | 39.36316 | 3.791292 | 3.64E-05 |
| CDO1 | 3.877397 | 1.642735 | -1.23899 | 2.03E-09 |
| CISD2 | 18.07036 | 37.44677 | 1.051216 | 1.27E-22 |
| CYBB | 11.86999 | 27.94011 | 1.235018 | 1.12E-05 |
| DDIT3 | 15.8421 | 48.04777 | 1.600706 | 1.47E-14 |
| DDIT4 | 125.6916 | 331.3184 | 1.39833 | 1.98E-10 |
| DRD4 | 0.435821 | 1.869892 | 2.101147 | 3.04E-11 |
| DRD5 | 0.035351 | 0.403236 | 3.511788 | 4.97E-10 |
| EGFR | 44.22186 | 128.3899 | 1.5377 | 1.75E-08 |
| ELANE | 0.994969 | 0.16708 | -2.57411 | 3.95E-10 |
| FANCD2 | 3.299367 | 7.802905 | 1.241822 | 7.83E-13 |
| FTL | 2629.25 | 6478.861 | 1.301089 | 3.09E-14 |
| G6PD | 63.53959 | 134.3553 | 1.080325 | 6.13E-05 |
| GCLC | 20.31385 | 42.70614 | 1.07198 | 5.49E-05 |
| GPT2 | 58.18057 | 24.66142 | -1.23828 | 3.00E-17 |
| GSDMB | 2.517971 | 6.283959 | 1.319412 | 1.24E-10 |
| GSDMD | 21.3268 | 51.95119 | 1.284489 | 4.30E-16 |
| GSDME | 2.564806 | 15.27305 | 2.574066 | 4.17E-21 |
| GZMA | 11.04465 | 37.26482 | 1.754467 | 1.30E-05 |
| GZMB | 9.164367 | 29.77701 | 1.700092 | 8.37E-09 |
| HELLS | 2.353808 | 7.496628 | 1.671245 | 1.47E-15 |
| HIF1A | 92.0426 | 216.2162 | 1.232101 | 9.76E-14 |
| HSF1 | 37.64405 | 79.726 | 1.082628 | 1.45E-22 |
| IFNG | 0.390934 | 1.997371 | 2.353104 | 5.70E-09 |
| IL1A | 11.8754 | 69.02461 | 2.539135 | 4.69E-10 |
| IL1B | 10.2795 | 41.12488 | 2.000242 | 3.16E-08 |
| IL33 | 39.5604 | 15.00666 | -1.39845 | 2.23E-12 |
| LINC00472 | 0.392283 | 0.192976 | -1.02347 | 0.005731 |
| LPIN1 | 12.77148 | 5.86005 | -1.12394 | 7.91E-11 |
| LURAP1L | 10.40471 | 21.41638 | 1.041479 | 1.78E-06 |
| MIOX | 0.034896 | 1.18893 | 5.090443 | 8.21E-12 |
| MT3 | 1.949017 | 0.612382 | -1.67024 | 8.60E-07 |
| NCF2 | 12.56776 | 37.0646 | 1.560315 | 5.82E-17 |
| NGB | 0.104502 | 2.557712 | 4.613251 | 3.54E-06 |
| NLRP1 | 5.132662 | 11.8683 | 1.209335 | 3.50E-13 |
| NLRP6 | 0.156781 | 0.401794 | 1.357705 | 2.01E-05 |
| NLRP7 | 0.098322 | 2.182151 | 4.472095 | 6.85E-16 |
| NOX4 | 0.587616 | 2.988946 | 2.346692 | 5.02E-18 |
| NOX5 | 0.096285 | 0.350002 | 1.861988 | 4.77E-07 |
| PANX1 | 23.1214 | 52.50026 | 1.183096 | 1.88E-12 |
| PIK3CA | 6.665924 | 14.10154 | 1.080976 | 6.30E-13 |
| PLCG1 | 9.890336 | 22.67351 | 1.196916 | 1.43E-20 |
| PLIN4 | 30.299 | 2.083025 | -3.86252 | 3.08E-17 |
| PML | 20.26711 | 48.74819 | 1.266208 | 6.10E-15 |
| PRKAA2 | 7.213093 | 1.642099 | -2.13508 | 9.46E-11 |
| PSAT1 | 41.12895 | 84.00853 | 1.030382 | 4.92E-07 |
| PTGS2 | 8.263021 | 34.66281 | 2.068647 | 7.28E-05 |
| PYCARD | 47.98479 | 96.8044 | 1.012495 | 2.62E-11 |
| RGS4 | 0.757181 | 6.470392 | 3.095144 | 5.62E-18 |
| RRM2 | 22.79896 | 66.19831 | 1.537826 | 2.06E-16 |
| SCD | 138.2098 | 286.5376 | 1.051864 | 9.28E-08 |
| SLC1A4 | 19.28278 | 52.6926 | 1.450287 | 1.14E-14 |
| SLC1A5 | 107.1341 | 221.2611 | 1.046332 | 4.86E-14 |
| SLC2A1 | 172.1239 | 752.5875 | 2.128412 | 7.57E-19 |
| SLC2A6 | 1.496314 | 9.101116 | 2.604631 | 3.72E-22 |
| SLC3A2 | 89.98396 | 247.4285 | 1.459272 | 2.76E-20 |
| SLC7A11 | 4.175245 | 18.32113 | 2.133576 | 2.75E-05 |
| SLC7A5 | 156.7652 | 462.3673 | 1.560434 | 2.01E-12 |
| SOCS1 | 6.861916 | 34.42469 | 2.326761 | 1.32E-21 |
| SRXN1 | 3.067886 | 6.316011 | 1.041769 | 0.00428 |
| STMN1 | 31.51435 | 104.794 | 1.733476 | 8.52E-20 |
| TF | 26.67238 | 3.293836 | -3.01751 | 4.67E-17 |
| TFR2 | 0.48359 | 1.721562 | 1.831862 | 1.54E-07 |
| TFRC | 33.23405 | 122.7819 | 1.885364 | 1.50E-14 |
| TGFBR1 | 17.56039 | 40.27016 | 1.197386 | 4.48E-16 |
| TNF | 4.04207 | 8.559719 | 1.082469 | 0.001558 |
| TNFAIP3 | 30.81117 | 77.90514 | 1.338265 | 1.10E-10 |
| TP63 | 88.36686 | 236.2065 | 1.418471 | 1.53E-14 |
| TRIB3 | 9.306681 | 29.38088 | 1.658539 | 1.37E-15 |
| TXNRD1 | 24.85262 | 68.96596 | 1.472486 | 2.78E-08 |
| ULK1 | 16.13956 | 35.27806 | 1.12817 | 5.48E-17 |
| VEGFA | 11.55545 | 32.98971 | 1.513442 | 2.49E-14 |
| ZFP36 | 940.0529 | 451.6706 | -1.05747 | 5.66E-09 |
| ZFP69B | 1.145283 | 2.662442 | 1.217046 | 1.94E-12 |
