# Supplementary material for: Relationships of Ferroptosis and Pyroptosis-Related Genes with Clinical Prognosis and Tumor Immune Microenvironment in Head and Neck Squamous Cell Carcinoma
Source: Oxid Med Cell Longev. 2022 Oct 5;2022:3713929. doi: 10.1155/2022/3713929 (PMC9557253; doi:10.1155/2022/3713929)
Supplement: Supplementary 4 — Supplementary Table 3. Forty-eight prognostic-related ferroptosis and pyroptosis were obtained by univariate Cox regression analysis in TCGA-HNSC dataset. [file 3713929.f4.docx]

Supplementary table 3. Forty-eight prognostic related ferroptosis and pyroptosis was obtained by univariate Cox regression analysis in TCGA-HNSC dataset.

| id | HR | HR.95L | HR.95H | pvalue |
| --- | --- | --- | --- | --- |
| TRIB3 | 1.008904 | 1.005155 | 1.012667 | 3.06E-06 |
| SLC2A3 | 1.005941 | 1.003113 | 1.008778 | 3.74E-05 |
| FLT3 | 0.58626 | 0.453416 | 0.758025 | 4.64E-05 |
| FTH1 | 1.000542 | 1.00028 | 1.000804 | 5.04E-05 |
| BNIP3 | 1.008224 | 1.004166 | 1.012299 | 6.89E-05 |
| CYCS | 1.004657 | 1.002181 | 1.00714 | 0.000224 |
| HSPA5 | 1.000705 | 1.000308 | 1.001102 | 0.000494 |
| CDKN2A | 0.994618 | 0.991497 | 0.997749 | 0.000764 |
| ASNS | 1.00718 | 1.002881 | 1.011497 | 0.001045 |
| PRDX6 | 1.001589 | 1.000633 | 1.002547 | 0.001119 |
| NQO1 | 1.001037 | 1.000399 | 1.001676 | 0.001452 |
| ALB | 1.02643 | 1.009172 | 1.043984 | 0.002568 |
| GABARAPL2 | 1.009826 | 1.003399 | 1.016294 | 0.002686 |
| OTUB1 | 1.006187 | 1.002132 | 1.010258 | 0.002754 |
| NLRP2 | 1.010698 | 1.003669 | 1.017775 | 0.002802 |
| VDAC2 | 1.004011 | 1.001343 | 1.006687 | 0.003199 |
| ATP5MC3 | 1.003266 | 1.000962 | 1.005576 | 0.005451 |
| IL1A | 1.001256 | 1.000358 | 1.002154 | 0.006088 |
| G6PD | 1.000961 | 1.000266 | 1.001657 | 0.006726 |
| ATG5 | 1.013421 | 1.003532 | 1.023406 | 0.007703 |
| NOX3 | 2.229587 | 1.228043 | 4.047951 | 0.008412 |
| TXNRD1 | 1.001429 | 1.000346 | 1.002514 | 0.009724 |
| AURKA | 1.007418 | 1.001669 | 1.0132 | 0.011374 |
| NLRP1 | 0.975806 | 0.957323 | 0.994646 | 0.012068 |
| SELENOS | 1.008837 | 1.001723 | 1.016001 | 0.014825 |
| GPX2 | 1.000281 | 1.000054 | 1.000509 | 0.01544 |
| ALOX12B | 0.996066 | 0.992792 | 0.999352 | 0.018973 |
| GZMB | 0.99441 | 0.989755 | 0.999087 | 0.019206 |
| YWHAE | 1.001015 | 1.00016 | 1.001871 | 0.019905 |
| EGFR | 1.000524 | 1.000076 | 1.000973 | 0.021945 |
| MAP3K5 | 0.989158 | 0.979786 | 0.998621 | 0.024825 |
| MAPK9 | 1.033776 | 1.004163 | 1.064262 | 0.025085 |
| IL6 | 1.001873 | 1.000208 | 1.003542 | 0.027447 |
| CISD2 | 1.009323 | 1.001023 | 1.017691 | 0.027621 |
| GSDME | 1.011233 | 1.001122 | 1.021446 | 0.029358 |
| SLC7A11 | 1.003227 | 1.000303 | 1.006159 | 0.030497 |
| MAP1LC3A | 0.99167 | 0.984062 | 0.999337 | 0.033267 |
| HSF1 | 1.004582 | 1.000291 | 1.008893 | 0.03634 |
| SOCS1 | 0.994394 | 0.98917 | 0.999645 | 0.036414 |
| CAV1 | 1.000367 | 1.000019 | 1.000715 | 0.03867 |
| GABARAPL1 | 1.004456 | 1.000199 | 1.008731 | 0.040195 |
| DDIT4 | 1.000413 | 1.000018 | 1.000807 | 0.040226 |
| CD44 | 1.000558 | 1.000016 | 1.0011 | 0.043741 |
| SLC2A6 | 1.015472 | 1.000347 | 1.030825 | 0.044927 |
| CXCL2 | 1.004513 | 1.000061 | 1.008986 | 0.046959 |
| SRXN1 | 1.012261 | 1.000114 | 1.024555 | 0.047881 |
| ACSL3 | 1.008176 | 1.00002 | 1.016399 | 0.049439 |
| SLC3A2 | 1.000887 | 1.000001 | 1.001773 | 0.049657 |
