# Supplementary material for: Relationships of Ferroptosis and Pyroptosis-Related Genes with Clinical Prognosis and Tumor Immune Microenvironment in Head and Neck Squamous Cell Carcinoma
Source: Oxid Med Cell Longev. 2022 Oct 5;2022:3713929. doi: 10.1155/2022/3713929 (PMC9557253; doi:10.1155/2022/3713929)
Supplement: Supplementary 5 — Supplementary Table 4. One thousand four hundred and sixty-seven DEGs between cluster A and cluster B were obtained through differential expression analysis in TCGA-HNSC dataset. [file 3713929.f5.DOCX]

Supplementary table 4. One thousand four hundred and sixty-seven DEGs between clusterA and clusterB was obtained through differential expression analysis in TCGA-HNSC dataset.

| gene | conMean | treatMean | logFC | pValue | fdr |
| --- | --- | --- | --- | --- | --- |
| CAV1 | 193.7502 | 713.0156 | 1.879736 | 2.28E-43 | 4.88E-39 |
| CAV2 | 49.39379 | 110.7589 | 1.165021 | 1.69E-38 | 1.81E-34 |
| PXN | 60.00301 | 134.4827 | 1.164314 | 5.72E-37 | 4.08E-33 |
| SNAI2 | 92.5569 | 189.8476 | 1.03643 | 4.17E-33 | 2.23E-29 |
| LAMC2 | 485.3384 | 1618.154 | 1.737286 | 5.27E-33 | 2.26E-29 |
| SERPINE1 | 267.6652 | 913.0058 | 1.770194 | 2.05E-31 | 7.30E-28 |
| ITGA3 | 138.9801 | 341.0383 | 1.295056 | 2.39E-31 | 7.30E-28 |
| ITGB4 | 359.954 | 746.5679 | 1.052461 | 4.75E-31 | 1.27E-27 |
| LAMA3 | 82.26713 | 293.8682 | 1.836781 | 5.86E-31 | 1.39E-27 |
| ACTN1 | 123.454 | 261.0688 | 1.080457 | 4.98E-30 | 1.07E-26 |
| NT5E | 25.96291 | 85.62432 | 1.721569 | 1.73E-29 | 3.37E-26 |
| PLEK2 | 116.304 | 273.365 | 1.232928 | 8.95E-29 | 1.60E-25 |
| ITGA6 | 349.031 | 846.1061 | 1.277484 | 1.02E-28 | 1.68E-25 |
| ITGA5 | 71.16381 | 169.2742 | 1.250146 | 1.16E-27 | 1.66E-24 |
| ITGB1-DT | 0.630942 | 2.042627 | 1.694846 | 1.55E-27 | 2.01E-24 |
| LINC00941 | 2.996538 | 8.908253 | 1.571846 | 1.59E-27 | 2.01E-24 |
| CARD10 | 27.40156 | 57.50958 | 1.069544 | 2.76E-27 | 3.28E-24 |
| LAMB3 | 545.9454 | 1285.148 | 1.235106 | 2.92E-27 | 3.29E-24 |
| VEGFC | 22.61698 | 67.01113 | 1.566994 | 4.05E-27 | 4.13E-24 |
| COL17A1 | 507.5749 | 1464.647 | 1.528861 | 2.21E-26 | 1.86E-23 |
| INHBA | 41.83844 | 124.9279 | 1.578195 | 3.10E-26 | 2.36E-23 |
| AC002066.1 | 0.306782 | 1.268747 | 2.04812 | 3.20E-26 | 2.36E-23 |
| BCAR3 | 14.88317 | 39.91376 | 1.423204 | 3.37E-26 | 2.40E-23 |
| LINC02535 | 0.350342 | 1.23282 | 1.815127 | 3.65E-26 | 2.52E-23 |
| MET | 41.74894 | 85.17612 | 1.028709 | 7.01E-26 | 4.69E-23 |
| COL4A6 | 10.87962 | 29.24129 | 1.426379 | 1.21E-25 | 7.41E-23 |
| RPSAP52 | 1.802226 | 5.893104 | 1.709248 | 1.86E-25 | 1.08E-22 |
| HS1BP3-IT1 | 1.155935 | 4.318604 | 1.901505 | 1.92E-25 | 1.08E-22 |
| CDK6 | 25.24641 | 57.58909 | 1.189718 | 2.07E-25 | 1.14E-22 |
| GSDME | 11.77305 | 26.17469 | 1.152684 | 2.25E-25 | 1.21E-22 |
| LINC02454 | 1.815343 | 5.042926 | 1.474019 | 3.76E-25 | 1.92E-22 |
| PLEC | 192.8454 | 393.2055 | 1.027839 | 5.46E-25 | 2.70E-22 |
| SEMA3C | 36.86274 | 102.1703 | 1.470741 | 2.96E-24 | 1.29E-21 |
| PDGFC | 11.94603 | 25.20264 | 1.077043 | 4.34E-24 | 1.86E-21 |
| FSTL3 | 47.81594 | 106.3965 | 1.153887 | 4.99E-24 | 2.09E-21 |
| MYOSLID | 6.917208 | 20.88482 | 1.594193 | 1.85E-23 | 7.19E-21 |
| TGFBI | 282.2527 | 773.9868 | 1.455321 | 2.04E-23 | 7.68E-21 |
| THBS1 | 89.64786 | 245.5702 | 1.453795 | 4.26E-23 | 1.55E-20 |
| EGFR | 89.62946 | 249.119 | 1.47479 | 4.78E-23 | 1.70E-20 |
| AREG | 99.31893 | 255.5191 | 1.363291 | 5.17E-23 | 1.79E-20 |
| GPR39 | 3.687141 | 8.596682 | 1.221277 | 9.82E-23 | 3.05E-20 |
| NRG1 | 9.483099 | 30.02256 | 1.662616 | 1.15E-22 | 3.46E-20 |
| AMIGO2 | 23.13349 | 54.72862 | 1.242313 | 2.70E-22 | 7.92E-20 |
| FEZ1 | 15.34853 | 40.04609 | 1.383561 | 3.20E-22 | 9.25E-20 |
| F3 | 151.4823 | 448.0937 | 1.564651 | 4.97E-22 | 1.40E-19 |
| LINC01179 | 0.177534 | 0.961123 | 2.436625 | 5.13E-22 | 1.43E-19 |
| PICART1 | 0.799141 | 1.788649 | 1.162348 | 5.80E-22 | 1.55E-19 |
| INPP4B | 4.063142 | 9.47648 | 1.221756 | 7.15E-22 | 1.89E-19 |
| TTLL11-IT1 | 1.157385 | 3.403227 | 1.556034 | 1.29E-21 | 3.25E-19 |
| SH2D5 | 8.271003 | 21.83384 | 1.400432 | 1.64E-21 | 4.05E-19 |
| PLAU | 288.1119 | 580.6192 | 1.010963 | 1.75E-21 | 4.26E-19 |
| INHBA-AS1 | 0.15802 | 0.451413 | 1.514342 | 5.00E-21 | 1.09E-18 |
| AC006159.1 | 0.189788 | 0.666508 | 1.812231 | 5.06E-21 | 1.10E-18 |
| ZBED2 | 25.81811 | 60.49389 | 1.228406 | 1.09E-20 | 2.21E-18 |
| PTHLH | 158.4525 | 349.8783 | 1.142803 | 1.45E-20 | 2.80E-18 |
| HMGA2 | 5.633942 | 20.54974 | 1.866904 | 2.26E-20 | 4.10E-18 |
| WNT7A | 6.266165 | 15.77133 | 1.33165 | 3.15E-20 | 5.44E-18 |
| AC037198.1 | 0.798388 | 2.113155 | 1.404238 | 3.71E-20 | 6.36E-18 |
| DDIT4 | 245.2757 | 599.3203 | 1.288923 | 3.97E-20 | 6.67E-18 |
| IL31RA | 1.971843 | 4.521717 | 1.197326 | 3.99E-20 | 6.67E-18 |
| PRSS23 | 30.5625 | 67.98031 | 1.153354 | 4.21E-20 | 6.94E-18 |
| LNCOG | 0.737657 | 2.675788 | 1.858942 | 7.38E-20 | 1.16E-17 |
| SH3TC2 | 0.538407 | 1.166762 | 1.115742 | 1.26E-19 | 1.89E-17 |
| CCBE1 | 0.996607 | 3.843872 | 1.947464 | 1.49E-19 | 2.20E-17 |
| EVA1A | 9.433976 | 23.04238 | 1.288352 | 1.86E-19 | 2.71E-17 |
| MT2P1 | 14.32075 | 38.11645 | 1.412307 | 2.07E-19 | 2.99E-17 |
| LINC01322 | 2.180275 | 8.655025 | 1.989028 | 2.22E-19 | 3.17E-17 |
| TENM2 | 38.67796 | 87.97995 | 1.185663 | 3.84E-19 | 5.24E-17 |
| CYP27B1 | 8.079058 | 20.2435 | 1.3252 | 4.20E-19 | 5.63E-17 |
| PDPN | 102.4616 | 214.8163 | 1.06802 | 4.43E-19 | 5.85E-17 |
| BSPRY | 23.5901 | 10.40475 | -1.18094 | 6.04E-19 | 7.84E-17 |
| MT2A | 909.9057 | 2164.015 | 1.249922 | 7.87E-19 | 9.97E-17 |
| AL049775.1 | 0.836944 | 2.362703 | 1.497235 | 1.16E-18 | 1.41E-16 |
| DCBLD2 | 17.06597 | 36.22238 | 1.085759 | 3.04E-18 | 3.47E-16 |
| AC010327.4 | 0.488612 | 1.367886 | 1.485188 | 3.31E-18 | 3.72E-16 |
| HLF | 6.171045 | 1.797553 | -1.77948 | 4.25E-18 | 4.72E-16 |
| LINC02560 | 23.89818 | 8.358375 | -1.51561 | 5.37E-18 | 5.87E-16 |
| LINC00911 | 0.706328 | 2.070478 | 1.551554 | 6.43E-18 | 6.95E-16 |
| NLRP3P1 | 0.904477 | 1.895134 | 1.067145 | 6.69E-18 | 7.16E-16 |
| AC243773.1 | 0.152498 | 0.589802 | 1.951444 | 7.33E-18 | 7.68E-16 |
| FLRT2 | 3.367555 | 9.007409 | 1.419411 | 8.22E-18 | 8.39E-16 |
| SCAT1 | 3.441008 | 7.468349 | 1.117958 | 8.64E-18 | 8.69E-16 |
| RPS16P2 | 0.496721 | 1.314929 | 1.404477 | 9.01E-18 | 8.93E-16 |
| SMIM5 | 2.937593 | 1.001664 | -1.55224 | 9.61E-18 | 9.40E-16 |
| SPACA4 | 1.109136 | 0.424908 | -1.38421 | 9.70E-18 | 9.44E-16 |
| CAMK2N1 | 24.00791 | 51.50076 | 1.101084 | 1.11E-17 | 1.07E-15 |
| LINC02551 | 0.794067 | 2.252637 | 1.504282 | 1.54E-17 | 1.40E-15 |
| MANCR | 1.994591 | 4.865426 | 1.286473 | 1.56E-17 | 1.41E-15 |
| CT62 | 0.411216 | 1.341572 | 1.705956 | 1.63E-17 | 1.45E-15 |
| LINC01546 | 0.534389 | 1.438501 | 1.428604 | 2.03E-17 | 1.76E-15 |
| DKK3 | 66.73478 | 133.8486 | 1.004091 | 2.22E-17 | 1.91E-15 |
| LINC01998 | 0.273522 | 1.094211 | 2.000164 | 4.46E-17 | 3.70E-15 |
| FAT1 | 53.21648 | 107 | 1.007666 | 4.81E-17 | 3.96E-15 |
| AC012065.2 | 1.724967 | 4.320994 | 1.324795 | 7.04E-17 | 5.67E-15 |
| RHOB | 249.6123 | 506.7841 | 1.021683 | 7.54E-17 | 5.98E-15 |
| LHX1 | 1.020648 | 3.033456 | 1.571476 | 7.61E-17 | 6.02E-15 |
| PPP4R4 | 3.077553 | 7.076664 | 1.201286 | 1.09E-16 | 8.41E-15 |
| LINC01356 | 0.500836 | 1.178448 | 1.234479 | 1.16E-16 | 8.83E-15 |
| CSF2 | 9.037094 | 32.02697 | 1.825356 | 1.65E-16 | 1.21E-14 |
| ZBTB7C | 20.88384 | 7.015521 | -1.57376 | 2.00E-16 | 1.45E-14 |
| EPHB6 | 16.09998 | 6.219698 | -1.37214 | 2.35E-16 | 1.68E-14 |
| SUSD4 | 26.61535 | 7.801599 | -1.77042 | 2.57E-16 | 1.81E-14 |
| AKNAD1 | 0.354956 | 0.711121 | 1.002457 | 2.70E-16 | 1.88E-14 |
| LHX1-DT | 0.826787 | 2.173519 | 1.394446 | 3.09E-16 | 2.12E-14 |
| BICDL2 | 19.38879 | 9.098209 | -1.09157 | 3.17E-16 | 2.16E-14 |
| MAB21L4 | 30.04543 | 9.432506 | -1.67143 | 3.30E-16 | 2.23E-14 |
| CDH13 | 14.36494 | 32.23202 | 1.165943 | 3.32E-16 | 2.24E-14 |
| MIR31HG | 7.667815 | 22.16843 | 1.531619 | 3.53E-16 | 2.35E-14 |
| AL161431.1 | 23.81267 | 54.47118 | 1.193764 | 3.97E-16 | 2.59E-14 |
| TM7SF2 | 20.26933 | 9.235454 | -1.13404 | 4.04E-16 | 2.62E-14 |
| LINP1 | 6.497246 | 17.97558 | 1.468138 | 4.69E-16 | 2.98E-14 |
| APCDD1L | 4.392 | 11.17441 | 1.347249 | 4.71E-16 | 2.99E-14 |
| IFNWP19 | 8.21759 | 23.37487 | 1.508171 | 4.81E-16 | 3.03E-14 |
| FAM3B | 12.33779 | 2.26546 | -2.44521 | 5.33E-16 | 3.33E-14 |
| NUPR1 | 44.44002 | 20.20646 | -1.13704 | 5.53E-16 | 3.43E-14 |
| LTBP1 | 66.77059 | 141.8034 | 1.086607 | 5.63E-16 | 3.47E-14 |
| LIPE-AS1 | 2.246681 | 1.024622 | -1.1327 | 7.20E-16 | 4.33E-14 |
| SERPINE2 | 37.03482 | 96.31131 | 1.378823 | 7.42E-16 | 4.45E-14 |
| HTR7 | 8.443368 | 17.69224 | 1.067226 | 7.59E-16 | 4.52E-14 |
| PLA2G3 | 14.05487 | 3.999056 | -1.81334 | 7.70E-16 | 4.57E-14 |
| TEX101 | 1.62108 | 0.432346 | -1.9067 | 7.78E-16 | 4.60E-14 |
| CSPG4 | 44.49503 | 115.3239 | 1.373976 | 8.74E-16 | 5.09E-14 |
| FKBP9P1 | 2.980997 | 7.664786 | 1.36245 | 9.49E-16 | 5.46E-14 |
| DNER | 2.596867 | 6.107037 | 1.233701 | 1.02E-15 | 5.83E-14 |
| SLC16A2 | 13.08203 | 30.32759 | 1.213045 | 1.44E-15 | 8.01E-14 |
| LRRN4 | 0.441821 | 1.937989 | 2.133028 | 1.47E-15 | 8.14E-14 |
| P3H2 | 54.1733 | 138.0478 | 1.349515 | 1.58E-15 | 8.67E-14 |
| PCDHGC5 | 2.000759 | 5.020037 | 1.327151 | 1.58E-15 | 8.70E-14 |
| AC002401.4 | 4.403384 | 11.60058 | 1.397512 | 1.78E-15 | 9.67E-14 |
| TENM3-AS1 | 1.042522 | 2.322243 | 1.155441 | 1.95E-15 | 1.05E-13 |
| FRMD5 | 0.673978 | 1.950933 | 1.533391 | 2.18E-15 | 1.17E-13 |
| AC104024.2 | 0.261017 | 0.609821 | 1.22424 | 2.31E-15 | 1.22E-13 |
| LINC01615 | 10.98234 | 25.86961 | 1.236073 | 2.99E-15 | 1.54E-13 |
| SCG5 | 4.44586 | 10.51473 | 1.241877 | 3.37E-15 | 1.73E-13 |
| LINC00460 | 3.123229 | 8.520747 | 1.447942 | 3.56E-15 | 1.82E-13 |
| AC090023.2 | 0.237577 | 0.574477 | 1.273854 | 3.68E-15 | 1.87E-13 |
| ANGPTL4 | 46.39663 | 114.5512 | 1.303901 | 4.17E-15 | 2.10E-13 |
| TENM3 | 6.374845 | 13.65226 | 1.098678 | 4.54E-15 | 2.28E-13 |
| SYT7 | 11.98617 | 25.473 | 1.087598 | 4.92E-15 | 2.45E-13 |
| DSCAM | 0.406051 | 1.279872 | 1.656267 | 5.35E-15 | 2.62E-13 |
| DISP2 | 0.833606 | 2.168886 | 1.379517 | 6.18E-15 | 2.98E-13 |
| TLL1 | 2.075101 | 6.939003 | 1.741547 | 7.21E-15 | 3.45E-13 |
| LINC01116 | 6.813004 | 13.69201 | 1.006971 | 7.98E-15 | 3.74E-13 |
| MMP10 | 228.3306 | 665.2322 | 1.542734 | 8.79E-15 | 4.07E-13 |
| L1CAM | 4.687632 | 17.26251 | 1.880711 | 1.00E-14 | 4.60E-13 |
| MSNP1 | 0.412593 | 0.837461 | 1.021303 | 1.01E-14 | 4.63E-13 |
| FAM87A | 0.287483 | 0.650518 | 1.178109 | 1.19E-14 | 5.34E-13 |
| CLDN4 | 144.7737 | 65.91905 | -1.13503 | 1.50E-14 | 6.62E-13 |
| TNFRSF13C | 3.243009 | 1.389727 | -1.22253 | 1.55E-14 | 6.82E-13 |
| ANTXR2 | 9.382848 | 22.60456 | 1.268516 | 1.60E-14 | 7.00E-13 |
| AC124276.1 | 0.398653 | 1.307501 | 1.713606 | 1.94E-14 | 8.37E-13 |
| AC090673.1 | 0.303908 | 0.664429 | 1.128483 | 2.13E-14 | 9.12E-13 |
| BIRC2 | 42.10312 | 101.7209 | 1.272617 | 2.88E-14 | 1.20E-12 |
| RNF157-AS1 | 0.447716 | 0.136116 | -1.71775 | 2.94E-14 | 1.23E-12 |
| NPM1P25 | 3.162075 | 1.489419 | -1.08612 | 2.99E-14 | 1.25E-12 |
| SLC6A11 | 7.014393 | 15.35995 | 1.130784 | 3.89E-14 | 1.57E-12 |
| RBBP8NL | 5.508464 | 2.537771 | -1.11809 | 3.95E-14 | 1.59E-12 |
| CYP2E1 | 1.833523 | 0.335777 | -2.44904 | 3.96E-14 | 1.59E-12 |
| IL1A | 49.93693 | 128.4781 | 1.363343 | 4.01E-14 | 1.61E-12 |
| NRTN | 4.628994 | 1.919414 | -1.27003 | 4.23E-14 | 1.68E-12 |
| WFDC21P | 102.8261 | 32.59432 | -1.65751 | 5.55E-14 | 2.15E-12 |
| DKK1 | 18.0875 | 59.53908 | 1.718844 | 7.67E-14 | 2.89E-12 |
| ARHGAP29 | 3.153292 | 6.894126 | 1.128509 | 7.88E-14 | 2.95E-12 |
| FOXA2 | 0.895313 | 1.803139 | 1.010046 | 8.27E-14 | 3.08E-12 |
| KHSRPP1 | 0.593669 | 1.35442 | 1.189945 | 9.82E-14 | 3.61E-12 |
| AL596244.1 | 3.6496 | 9.64275 | 1.401706 | 1.01E-13 | 3.68E-12 |
| EGFR-AS1 | 0.233134 | 0.925765 | 1.989485 | 1.07E-13 | 3.88E-12 |
| LINC02577 | 2.138405 | 4.629452 | 1.114307 | 1.13E-13 | 4.07E-12 |
| LINC00707 | 4.267943 | 12.83769 | 1.588772 | 1.20E-13 | 4.26E-12 |
| LINC01357 | 1.094656 | 2.470899 | 1.174559 | 1.24E-13 | 4.39E-12 |
| CYP26B1 | 14.14914 | 31.0959 | 1.136011 | 1.24E-13 | 4.40E-12 |
| TNC | 305.0722 | 637.4384 | 1.063135 | 1.38E-13 | 4.83E-12 |
| ATP13A4 | 5.461086 | 1.802989 | -1.5988 | 1.49E-13 | 5.20E-12 |
| SLC38A4 | 2.616318 | 6.858709 | 1.390399 | 1.74E-13 | 5.98E-12 |
| LINC02029 | 0.1534 | 0.401196 | 1.387007 | 1.80E-13 | 6.19E-12 |
| COLCA1 | 5.194138 | 0.470206 | -3.46552 | 2.01E-13 | 6.86E-12 |
| GDPD3 | 14.86311 | 6.474842 | -1.19882 | 2.03E-13 | 6.89E-12 |
| AL161645.1 | 0.658998 | 0.115895 | -2.50745 | 2.18E-13 | 7.35E-12 |
| GCNT1P3 | 0.174046 | 0.398465 | 1.194987 | 2.32E-13 | 7.79E-12 |
| SCNN1B | 15.78577 | 5.569013 | -1.50313 | 2.35E-13 | 7.90E-12 |
| SRPX | 20.73443 | 46.65124 | 1.169887 | 3.05E-13 | 1.00E-11 |
| AC090409.1 | 1.183603 | 2.666359 | 1.171686 | 3.16E-13 | 1.03E-11 |
| AC097451.1 | 0.250211 | 1.259131 | 2.331211 | 3.17E-13 | 1.03E-11 |
| SUN3 | 0.983682 | 2.579309 | 1.390721 | 3.45E-13 | 1.11E-11 |
| GGT6 | 29.82996 | 14.23304 | -1.06752 | 3.61E-13 | 1.15E-11 |
| PLAC1 | 3.179733 | 6.644907 | 1.063344 | 3.73E-13 | 1.19E-11 |
| ITGB6 | 63.38041 | 129.6166 | 1.032142 | 3.75E-13 | 1.19E-11 |
| LINC01704 | 0.330364 | 0.930907 | 1.49458 | 4.36E-13 | 1.37E-11 |
| REPIN1-AS1 | 2.172239 | 0.917275 | -1.24376 | 4.84E-13 | 1.51E-11 |
| SPRR3 | 1553.499 | 275.3786 | -2.49603 | 5.52E-13 | 1.70E-11 |
| PRSS27 | 19.01403 | 6.181771 | -1.62097 | 7.44E-13 | 2.24E-11 |
| STX19 | 7.182702 | 3.516231 | -1.0305 | 8.48E-13 | 2.54E-11 |
| MYH16 | 0.479743 | 1.305104 | 1.443832 | 9.22E-13 | 2.75E-11 |
| PCDH7 | 14.96677 | 30.47352 | 1.025793 | 1.01E-12 | 3.00E-11 |
| LINC00973 | 0.437477 | 1.276186 | 1.544559 | 1.15E-12 | 3.35E-11 |
| TIMP3 | 4.547037 | 10.01444 | 1.139083 | 1.22E-12 | 3.53E-11 |
| AC010754.1 | 0.825025 | 2.348859 | 1.509451 | 1.28E-12 | 3.69E-11 |
| RBP7 | 15.47307 | 5.739009 | -1.43089 | 1.53E-12 | 4.35E-11 |
| KLRB1 | 4.41677 | 1.830641 | -1.27064 | 1.53E-12 | 4.35E-11 |
| IQCN | 0.790217 | 0.291978 | -1.43639 | 1.60E-12 | 4.52E-11 |
| LINC00880 | 0.142082 | 0.39294 | 1.467591 | 1.63E-12 | 4.60E-11 |
| FST | 97.59322 | 195.4544 | 1.001979 | 1.92E-12 | 5.32E-11 |
| CYP4F29P | 1.894602 | 0.533641 | -1.82795 | 1.94E-12 | 5.39E-11 |
| ANKRD35 | 13.50078 | 6.361638 | -1.08557 | 1.98E-12 | 5.47E-11 |
| AL356867.1 | 5.455308 | 1.465343 | -1.89642 | 2.01E-12 | 5.56E-11 |
| ELF3 | 69.09959 | 33.12154 | -1.06091 | 2.19E-12 | 6.01E-11 |
| TMPRSS2 | 9.056283 | 1.904993 | -2.24913 | 2.22E-12 | 6.07E-11 |
| LEXM | 2.412409 | 0.906239 | -1.41251 | 2.33E-12 | 6.35E-11 |
| IRX6 | 4.189747 | 0.625333 | -2.74417 | 2.41E-12 | 6.52E-11 |
| SLITRK6 | 10.76968 | 31.5401 | 1.550212 | 2.41E-12 | 6.52E-11 |
| AKR7A2P1 | 0.406137 | 0.853914 | 1.072123 | 2.57E-12 | 6.89E-11 |
| AL445531.1 | 0.422321 | 0.188171 | -1.1663 | 2.58E-12 | 6.90E-11 |
| FLRT3 | 12.94354 | 30.53126 | 1.238055 | 2.92E-12 | 7.65E-11 |
| WTAPP1 | 0.644909 | 1.567295 | 1.281109 | 3.25E-12 | 8.43E-11 |
| LIPM | 4.57252 | 2.10172 | -1.12142 | 3.43E-12 | 8.85E-11 |
| MT1L | 21.34161 | 60.61038 | 1.505896 | 3.56E-12 | 9.13E-11 |
| KRT13 | 2834.414 | 484.1576 | -2.5495 | 4.06E-12 | 1.02E-10 |
| YAP1 | 74.58703 | 170.9536 | 1.196608 | 4.52E-12 | 1.13E-10 |
| CXCL17 | 102.775 | 38.52587 | -1.41559 | 4.95E-12 | 1.22E-10 |
| TNPO1P3 | 0.159487 | 0.400082 | 1.326858 | 5.14E-12 | 1.26E-10 |
| LUARIS | 0.964585 | 2.162096 | 1.164451 | 5.36E-12 | 1.31E-10 |
| FUT3 | 27.5176 | 11.46651 | -1.26293 | 6.43E-12 | 1.55E-10 |
| NAV3 | 1.407445 | 3.148555 | 1.161611 | 6.56E-12 | 1.57E-10 |
| AP003068.4 | 1.650805 | 0.621555 | -1.40922 | 7.52E-12 | 1.79E-10 |
| ICA1 | 3.963776 | 1.497611 | -1.40421 | 7.86E-12 | 1.86E-10 |
| OR10Y1P | 0.253457 | 0.612051 | 1.271909 | 8.01E-12 | 1.89E-10 |
| AL691482.3 | 2.499122 | 1.087513 | -1.20039 | 8.96E-12 | 2.08E-10 |
| CYP2C18 | 13.64962 | 4.758543 | -1.52027 | 9.22E-12 | 2.13E-10 |
| PCNPP3 | 0.856584 | 1.891811 | 1.143102 | 1.14E-11 | 2.59E-10 |
| PCP4L1 | 19.21961 | 5.759325 | -1.73861 | 1.17E-11 | 2.65E-10 |
| FGF5 | 0.255441 | 1.432807 | 2.487782 | 1.17E-11 | 2.65E-10 |
| LRATD1 | 29.41255 | 13.99416 | -1.07161 | 1.20E-11 | 2.69E-10 |
| CD52 | 56.88077 | 26.69152 | -1.09156 | 1.26E-11 | 2.82E-10 |
| AC022165.1 | 0.304965 | 0.61771 | 1.018286 | 1.33E-11 | 2.96E-10 |
| FAM3D | 25.00021 | 8.988489 | -1.47579 | 1.35E-11 | 3.00E-10 |
| MISP3 | 5.313659 | 2.529763 | -1.0707 | 1.45E-11 | 3.21E-10 |
| ELF3-AS1 | 3.449097 | 1.701072 | -1.01977 | 1.52E-11 | 3.35E-10 |
| TNFRSF13B | 0.629292 | 0.212601 | -1.56558 | 1.76E-11 | 3.82E-10 |
| AC022497.1 | 0.258304 | 0.123831 | -1.0607 | 1.92E-11 | 4.16E-10 |
| FTLP10 | 0.862807 | 0.120434 | -2.8408 | 1.97E-11 | 4.25E-10 |
| CEACAM5 | 103.4618 | 37.61507 | -1.45972 | 2.00E-11 | 4.31E-10 |
| SYNGR1 | 8.344254 | 4.069959 | -1.03577 | 2.26E-11 | 4.79E-10 |
| AL391001.1 | 1.297812 | 0.571819 | -1.18245 | 2.29E-11 | 4.84E-10 |
| TRIML2 | 1.490075 | 4.964068 | 1.736138 | 2.38E-11 | 5.03E-10 |
| TJP3 | 8.657182 | 2.84844 | -1.60373 | 2.43E-11 | 5.12E-10 |
| GAS6-DT | 1.950144 | 4.701127 | 1.269426 | 2.44E-11 | 5.14E-10 |
| CEACAM7 | 10.40483 | 3.054614 | -1.76819 | 2.46E-11 | 5.18E-10 |
| ELFN2 | 0.246301 | 1.054589 | 2.098187 | 2.58E-11 | 5.41E-10 |
| MYH14 | 73.21418 | 32.57055 | -1.16855 | 2.64E-11 | 5.51E-10 |
| SMAD5-AS1 | 0.462593 | 0.197865 | -1.22523 | 2.72E-11 | 5.67E-10 |
| JPT1P1 | 0.286646 | 0.692124 | 1.27176 | 2.97E-11 | 6.14E-10 |
| AC245041.1 | 26.9045 | 59.82325 | 1.152859 | 3.00E-11 | 6.19E-10 |
| NUP210 | 34.65188 | 15.31069 | -1.17839 | 3.10E-11 | 6.38E-10 |
| SFTA1P | 2.575178 | 8.557577 | 1.73253 | 3.61E-11 | 7.34E-10 |
| FUT6 | 6.987271 | 1.653822 | -2.07892 | 3.67E-11 | 7.46E-10 |
| C15orf62 | 7.442664 | 3.473068 | -1.09961 | 3.71E-11 | 7.53E-10 |
| MAL | 133.1272 | 15.72897 | -3.08131 | 3.73E-11 | 7.56E-10 |
| ASCL4 | 0.591572 | 0.03553 | -4.05746 | 3.75E-11 | 7.58E-10 |
| CCDC177 | 0.674572 | 0.282413 | -1.25617 | 3.84E-11 | 7.72E-10 |
| SOWAHB | 7.9564 | 3.612378 | -1.13917 | 4.31E-11 | 8.58E-10 |
| LINC01315 | 4.753631 | 1.596158 | -1.57443 | 4.53E-11 | 8.97E-10 |
| AL049775.3 | 0.230971 | 0.503586 | 1.124523 | 4.71E-11 | 9.31E-10 |
| POU2AF1 | 8.169577 | 2.54907 | -1.68029 | 5.41E-11 | 1.05E-09 |
| TRPV6 | 0.673993 | 0.179617 | -1.90781 | 5.65E-11 | 1.09E-09 |
| HPGD | 7.442197 | 2.441667 | -1.60786 | 5.68E-11 | 1.10E-09 |
| AC024592.2 | 0.815716 | 0.253745 | -1.68469 | 5.78E-11 | 1.11E-09 |
| TMPRSS11B | 10.0195 | 1.471015 | -2.76793 | 5.87E-11 | 1.13E-09 |
| PLAC8 | 16.17508 | 5.663513 | -1.514 | 6.01E-11 | 1.15E-09 |
| PPM1L | 5.198188 | 2.230554 | -1.22061 | 6.07E-11 | 1.16E-09 |
| CYP4F12 | 3.411652 | 0.873779 | -1.96513 | 6.13E-11 | 1.17E-09 |
| AC004687.1 | 2.847932 | 1.249151 | -1.18897 | 6.43E-11 | 1.22E-09 |
| FAM83E | 17.13391 | 6.744591 | -1.34505 | 6.62E-11 | 1.25E-09 |
| LNCAROD | 3.644645 | 11.98757 | 1.717689 | 6.65E-11 | 1.25E-09 |
| TPRG1 | 5.684737 | 2.819749 | -1.01153 | 7.32E-11 | 1.36E-09 |
| COLCA2 | 4.641075 | 1.466144 | -1.66243 | 7.34E-11 | 1.37E-09 |
| NCCRP1 | 167.2359 | 66.82768 | -1.32337 | 8.82E-11 | 1.60E-09 |
| TTC39C-AS1 | 0.585239 | 0.289176 | -1.01707 | 1.07E-10 | 1.90E-09 |
| KANK4 | 3.182547 | 7.868569 | 1.305919 | 1.10E-10 | 1.94E-09 |
| SCNN1A | 53.61029 | 23.73414 | -1.17555 | 1.14E-10 | 2.02E-09 |
| RPS27P16 | 2.282422 | 5.252271 | 1.202376 | 1.17E-10 | 2.06E-09 |
| ARSF | 0.508891 | 0.12136 | -2.06806 | 1.19E-10 | 2.10E-09 |
| AC005083.1 | 3.56953 | 1.627907 | -1.13272 | 1.23E-10 | 2.16E-09 |
| GATM | 8.400413 | 3.838068 | -1.13008 | 1.25E-10 | 2.18E-09 |
| AC124276.2 | 0.159786 | 0.499216 | 1.643527 | 1.27E-10 | 2.21E-09 |
| AL512631.1 | 0.402269 | 0.129184 | -1.63873 | 1.54E-10 | 2.64E-09 |
| TMPRSS11A | 42.91885 | 12.48585 | -1.78132 | 1.70E-10 | 2.89E-09 |
| ADAMTS15 | 7.978806 | 16.84751 | 1.07829 | 1.72E-10 | 2.92E-09 |
| CYP2C9 | 0.547563 | 0.21248 | -1.3657 | 1.94E-10 | 3.26E-09 |
| SMARCE1P6 | 0.279751 | 0.123769 | -1.17649 | 2.05E-10 | 3.41E-09 |
| SKAP1 | 5.364456 | 2.322746 | -1.2076 | 2.08E-10 | 3.46E-09 |
| AP000619.1 | 0.297375 | 1.082186 | 1.863592 | 2.13E-10 | 3.54E-09 |
| SLC27A2 | 4.132138 | 1.332019 | -1.63327 | 2.18E-10 | 3.61E-09 |
| ADH7 | 71.79298 | 24.93461 | -1.52569 | 2.34E-10 | 3.83E-09 |
| SPINK5 | 157.39 | 55.16999 | -1.51239 | 2.38E-10 | 3.89E-09 |
| TTC9 | 23.67005 | 11.78082 | -1.00662 | 2.48E-10 | 4.05E-09 |
| ARHGEF26 | 7.550573 | 2.525066 | -1.58027 | 2.52E-10 | 4.10E-09 |
| SPOCK1 | 11.49408 | 27.22795 | 1.244197 | 2.71E-10 | 4.38E-09 |
| SLC6A2 | 2.690777 | 6.372434 | 1.243822 | 2.82E-10 | 4.54E-09 |
| AC002511.2 | 1.187788 | 0.296397 | -2.00267 | 2.92E-10 | 4.69E-09 |
| AC117422.1 | 0.167738 | 0.424648 | 1.340058 | 2.93E-10 | 4.70E-09 |
| C5orf66-AS1 | 12.80734 | 3.548372 | -1.85174 | 3.06E-10 | 4.87E-09 |
| MFSD6L | 1.029231 | 0.410401 | -1.32646 | 3.26E-10 | 5.15E-09 |
| IL24 | 21.88962 | 60.94884 | 1.477352 | 3.29E-10 | 5.19E-09 |
| C8G | 9.814905 | 1.085067 | -3.17719 | 3.31E-10 | 5.21E-09 |
| CALML5 | 729.7058 | 308.9403 | -1.23999 | 3.40E-10 | 5.36E-09 |
| AC108718.1 | 0.331925 | 0.759447 | 1.194092 | 3.46E-10 | 5.44E-09 |
| PDZK1 | 0.818304 | 1.853876 | 1.179836 | 3.49E-10 | 5.48E-09 |
| SOX21 | 19.06099 | 7.210326 | -1.40249 | 3.71E-10 | 5.79E-09 |
| FN1 | 365.1432 | 773.0653 | 1.082128 | 4.36E-10 | 6.70E-09 |
| GZMM | 6.122968 | 2.708186 | -1.1769 | 4.46E-10 | 6.84E-09 |
| TMPRSS11BNL | 2.183984 | 0.362229 | -2.59199 | 4.66E-10 | 7.08E-09 |
| AL021407.1 | 0.334481 | 0.110404 | -1.59913 | 4.80E-10 | 7.28E-09 |
| SLC26A9 | 5.633984 | 1.555381 | -1.85689 | 4.82E-10 | 7.30E-09 |
| SHISA8 | 0.727042 | 0.244152 | -1.57426 | 4.82E-10 | 7.30E-09 |
| AL035661.1 | 22.84962 | 10.35459 | -1.1419 | 5.16E-10 | 7.76E-09 |
| SBK1 | 5.930284 | 1.599053 | -1.89088 | 5.63E-10 | 8.36E-09 |
| ILDR1 | 10.6884 | 4.822857 | -1.14809 | 5.71E-10 | 8.46E-09 |
| MUC15 | 11.6031 | 4.454212 | -1.38127 | 5.88E-10 | 8.69E-09 |
| RASGRP1 | 7.507643 | 3.579274 | -1.06869 | 6.17E-10 | 9.07E-09 |
| KRT78 | 58.33331 | 12.71848 | -2.19739 | 6.37E-10 | 9.33E-09 |
| MIR8071-2 | 11.42896 | 4.364431 | -1.38883 | 6.41E-10 | 9.38E-09 |
| MT1E | 150.8435 | 311.8359 | 1.047735 | 6.51E-10 | 9.50E-09 |
| MS4A1 | 3.772303 | 1.051097 | -1.84355 | 6.90E-10 | 9.98E-09 |
| OCA2 | 2.357445 | 0.553412 | -2.0908 | 6.94E-10 | 1.00E-08 |
| LY9 | 0.958692 | 0.444738 | -1.10811 | 7.23E-10 | 1.04E-08 |
| ATG9B | 4.064006 | 1.754822 | -1.21158 | 7.74E-10 | 1.11E-08 |
| AC079313.1 | 0.342307 | 0.931387 | 1.444091 | 7.86E-10 | 1.12E-08 |
| AL159169.2 | 0.983426 | 0.46948 | -1.06675 | 7.93E-10 | 1.13E-08 |
| AC008406.3 | 1.011528 | 0.308714 | -1.71219 | 8.03E-10 | 1.14E-08 |
| KLHL4 | 0.459258 | 1.471861 | 1.680265 | 8.19E-10 | 1.16E-08 |
| AC002511.1 | 0.614458 | 0.18054 | -1.767 | 8.53E-10 | 1.21E-08 |
| MYO3A | 2.046592 | 0.473829 | -2.11079 | 8.61E-10 | 1.21E-08 |
| FOLR3 | 5.763738 | 17.44043 | 1.597359 | 8.65E-10 | 1.22E-08 |
| LORICRIN | 14.62502 | 5.096769 | -1.52078 | 8.83E-10 | 1.24E-08 |
| ATP6V0E2 | 11.20631 | 4.915982 | -1.18876 | 9.22E-10 | 1.29E-08 |
| ACTBL2 | 0.281222 | 0.572954 | 1.026706 | 9.42E-10 | 1.32E-08 |
| AC107308.1 | 0.653862 | 1.677854 | 1.359559 | 9.73E-10 | 1.36E-08 |
| IGKV2D-24 | 7.010457 | 1.68641 | -2.05555 | 1.00E-09 | 1.38E-08 |
| KRT33A | 0.752349 | 0.356313 | -1.07826 | 1.01E-09 | 1.39E-08 |
| ZMAT1 | 1.073715 | 0.365083 | -1.55632 | 1.04E-09 | 1.43E-08 |
| CHIT1 | 8.294815 | 1.85072 | -2.16412 | 1.04E-09 | 1.43E-08 |
| ROBO2 | 0.905919 | 0.301179 | -1.58876 | 1.06E-09 | 1.46E-08 |
| AC131953.1 | 0.170039 | 0.405188 | 1.252726 | 1.12E-09 | 1.53E-08 |
| AL159169.3 | 0.28453 | 0.093328 | -1.6082 | 1.18E-09 | 1.60E-08 |
| DYNAP | 3.624299 | 0.219149 | -4.04772 | 1.19E-09 | 1.61E-08 |
| AL157935.1 | 0.9756 | 0.475501 | -1.03684 | 1.22E-09 | 1.65E-08 |
| PBX1 | 6.889566 | 2.976791 | -1.21065 | 1.30E-09 | 1.76E-08 |
| FFAR4 | 0.601657 | 0.238546 | -1.33467 | 1.31E-09 | 1.76E-08 |
| DLEC1 | 0.684535 | 0.175125 | -1.96674 | 1.32E-09 | 1.78E-08 |
| RALGPS1 | 2.313899 | 1.099134 | -1.07396 | 1.36E-09 | 1.83E-08 |
| MAP4K1 | 7.383444 | 3.659387 | -1.01269 | 1.49E-09 | 1.99E-08 |
| AL021328.1 | 0.542708 | 0.154216 | -1.81522 | 1.59E-09 | 2.11E-08 |
| L3MBTL4 | 2.271236 | 1.125674 | -1.01269 | 1.59E-09 | 2.12E-08 |
| NPPC | 6.645744 | 3.243061 | -1.03507 | 1.59E-09 | 2.12E-08 |
| GPR160 | 6.347309 | 2.465772 | -1.36411 | 1.61E-09 | 2.14E-08 |
| LINC01781 | 0.526621 | 0.255266 | -1.04476 | 1.62E-09 | 2.15E-08 |
| AC253536.4 | 1.821805 | 0.356848 | -2.35199 | 1.64E-09 | 2.17E-08 |
| AL359979.1 | 0.76477 | 0.293057 | -1.38384 | 1.66E-09 | 2.19E-08 |
| LINC01805 | 1.301967 | 0.588992 | -1.14437 | 1.70E-09 | 2.24E-08 |
| EYA2 | 21.81775 | 6.922439 | -1.65615 | 1.72E-09 | 2.27E-08 |
| TGM3 | 294.5009 | 36.00138 | -3.03215 | 1.87E-09 | 2.44E-08 |
| AP000553.2 | 0.546618 | 0.197811 | -1.46641 | 2.10E-09 | 2.72E-08 |
| MZB1 | 35.21603 | 13.13803 | -1.42248 | 2.10E-09 | 2.72E-08 |
| TFPI2 | 6.559622 | 52.29214 | 2.994909 | 2.11E-09 | 2.73E-08 |
| EPHX3 | 39.82426 | 13.75356 | -1.53384 | 2.17E-09 | 2.79E-08 |
| RN7SL151P | 0.398101 | 1.115683 | 1.486722 | 2.27E-09 | 2.90E-08 |
| CD79A | 36.36104 | 11.67971 | -1.63839 | 2.28E-09 | 2.91E-08 |
| CXorf65 | 0.627609 | 0.298933 | -1.07004 | 2.32E-09 | 2.97E-08 |
| LINC01206 | 1.512027 | 0.224645 | -2.75076 | 2.37E-09 | 3.01E-08 |
| ACP3 | 10.03204 | 4.628327 | -1.11605 | 2.38E-09 | 3.02E-08 |
| IGLC7 | 38.47628 | 14.62327 | -1.3957 | 2.40E-09 | 3.05E-08 |
| AL121839.2 | 0.643698 | 0.274376 | -1.23023 | 2.43E-09 | 3.07E-08 |
| LINC02384 | 0.646163 | 0.317521 | -1.02505 | 2.44E-09 | 3.08E-08 |
| MYO7B | 1.155659 | 2.790784 | 1.271955 | 2.45E-09 | 3.10E-08 |
| AC005722.4 | 1.866602 | 0.464485 | -2.00671 | 2.55E-09 | 3.20E-08 |
| AC003092.1 | 0.250771 | 0.820024 | 1.709293 | 2.58E-09 | 3.24E-08 |
| AC005162.1 | 0.695726 | 0.235981 | -1.55985 | 2.72E-09 | 3.39E-08 |
| MUC21 | 24.96928 | 2.830641 | -3.14095 | 2.81E-09 | 3.48E-08 |
| ELAVL2 | 1.846716 | 4.501795 | 1.285538 | 2.82E-09 | 3.50E-08 |
| OR7E14P | 4.605951 | 1.863715 | -1.30532 | 2.90E-09 | 3.58E-08 |
| SEMA3A | 2.699886 | 6.612836 | 1.292371 | 2.90E-09 | 3.58E-08 |
| AL096816.1 | 0.297694 | 0.105971 | -1.49017 | 3.08E-09 | 3.77E-08 |
| KIAA0319 | 1.024572 | 0.254289 | -2.01048 | 3.40E-09 | 4.11E-08 |
| AC103563.7 | 0.676406 | 0.151176 | -2.16166 | 3.73E-09 | 4.47E-08 |
| AP001207.3 | 4.786353 | 2.252722 | -1.08726 | 3.80E-09 | 4.54E-08 |
| CYSRT1 | 77.36165 | 30.20989 | -1.3566 | 3.85E-09 | 4.59E-08 |
| IGLC3 | 949.4627 | 323.1913 | -1.55472 | 3.87E-09 | 4.61E-08 |
| IFNK | 0.640562 | 7.106183 | 3.471664 | 3.94E-09 | 4.68E-08 |
| MARCHF4 | 0.228277 | 0.461696 | 1.016156 | 4.04E-09 | 4.78E-08 |
| IGKV2-24 | 98.70506 | 25.62481 | -1.94558 | 4.04E-09 | 4.78E-08 |
| SPIB | 11.38823 | 1.385271 | -3.0393 | 4.07E-09 | 4.81E-08 |
| CSTA | 1355.711 | 651.4611 | -1.0573 | 4.14E-09 | 4.88E-08 |
| PLA2G2D | 4.327175 | 1.121616 | -1.94785 | 4.72E-09 | 5.50E-08 |
| ATP13A5 | 5.100972 | 0.747963 | -2.76973 | 4.77E-09 | 5.54E-08 |
| BFSP2 | 0.241277 | 0.115239 | -1.06607 | 4.83E-09 | 5.60E-08 |
| DNAH5 | 0.854289 | 1.790961 | 1.067938 | 4.86E-09 | 5.64E-08 |
| LINC01133 | 34.91363 | 15.15163 | -1.20432 | 5.58E-09 | 6.37E-08 |
| LRRC8D-DT | 2.274867 | 0.885631 | -1.361 | 6.15E-09 | 6.98E-08 |
| FOXC2 | 5.60542 | 12.15851 | 1.117072 | 6.24E-09 | 7.05E-08 |
| VSIG10L | 32.50591 | 12.54713 | -1.37334 | 6.40E-09 | 7.21E-08 |
| ARHGEF26-AS1 | 0.340541 | 0.084452 | -2.01162 | 6.66E-09 | 7.49E-08 |
| SOX21-AS1 | 6.924123 | 3.298492 | -1.06982 | 7.02E-09 | 7.83E-08 |
| RASGRF1 | 0.344961 | 0.823258 | 1.254911 | 7.15E-09 | 7.96E-08 |
| AC005392.2 | 17.07391 | 5.07719 | -1.74969 | 7.20E-09 | 8.01E-08 |
| ANK1 | 2.458329 | 4.997868 | 1.023635 | 7.31E-09 | 8.11E-08 |
| AC078820.1 | 0.637194 | 1.433524 | 1.169763 | 7.59E-09 | 8.40E-08 |
| TMPRSS11GP | 0.907715 | 0.336009 | -1.43374 | 7.72E-09 | 8.53E-08 |
| CD19 | 2.587802 | 1.031005 | -1.32768 | 7.92E-09 | 8.73E-08 |
| ADRA1B | 0.850053 | 2.058359 | 1.275869 | 8.04E-09 | 8.84E-08 |
| TRBC2 | 37.03229 | 17.54561 | -1.07767 | 8.27E-09 | 9.06E-08 |
| MIR600HG | 2.271005 | 1.121151 | -1.01835 | 8.41E-09 | 9.20E-08 |
| VPREB3 | 3.793748 | 1.681831 | -1.17359 | 8.77E-09 | 9.57E-08 |
| FCRL2 | 0.388299 | 0.134135 | -1.53348 | 9.03E-09 | 9.83E-08 |
| AC107464.2 | 0.794423 | 0.311675 | -1.34986 | 9.18E-09 | 9.98E-08 |
| CYP4B1 | 6.716741 | 2.470985 | -1.44268 | 9.38E-09 | 1.02E-07 |
| SLC6A4 | 0.652512 | 0.220168 | -1.5674 | 9.43E-09 | 1.02E-07 |
| SPTSSB | 5.60367 | 2.709985 | -1.04809 | 9.55E-09 | 1.03E-07 |
| FAM30A | 0.733664 | 0.283572 | -1.3714 | 9.77E-09 | 1.06E-07 |
| AC069224.1 | 0.744861 | 0.345993 | -1.10623 | 1.00E-08 | 1.08E-07 |
| LINC01871 | 10.87659 | 5.157191 | -1.07657 | 1.10E-08 | 1.17E-07 |
| MEIG1 | 0.511654 | 0.249048 | -1.03874 | 1.11E-08 | 1.18E-07 |
| IKZF3 | 6.225581 | 2.518621 | -1.30557 | 1.14E-08 | 1.21E-07 |
| MCF2L-AS1 | 2.665465 | 0.736994 | -1.85466 | 1.23E-08 | 1.29E-07 |
| PI15 | 3.420242 | 7.570208 | 1.146235 | 1.36E-08 | 1.42E-07 |
| TNFRSF17 | 4.449772 | 1.663901 | -1.41916 | 1.37E-08 | 1.43E-07 |
| IGHV3-19 | 5.178691 | 1.62602 | -1.67124 | 1.43E-08 | 1.49E-07 |
| ANKRD44-AS1 | 0.450671 | 0.214713 | -1.06967 | 1.47E-08 | 1.52E-07 |
| RASL11A | 11.26094 | 4.331015 | -1.37855 | 1.49E-08 | 1.55E-07 |
| AC116021.1 | 0.230866 | 0.473607 | 1.036636 | 1.53E-08 | 1.58E-07 |
| SAMD12-AS1 | 1.490725 | 0.433083 | -1.7833 | 1.53E-08 | 1.58E-07 |
| DHRS2 | 5.842005 | 18.79646 | 1.685925 | 1.64E-08 | 1.68E-07 |
| LCN12 | 0.602165 | 0.155571 | -1.95259 | 1.72E-08 | 1.76E-07 |
| CNFN | 1227.543 | 431.3717 | -1.50877 | 1.73E-08 | 1.77E-07 |
| AC010735.1 | 0.18434 | 0.457452 | 1.311253 | 1.73E-08 | 1.77E-07 |
| HNRNPA1P33 | 0.746828 | 1.631207 | 1.127092 | 1.77E-08 | 1.81E-07 |
| JAML | 8.782898 | 2.551584 | -1.7833 | 1.79E-08 | 1.82E-07 |
| LINC01644 | 0.817907 | 0.373871 | -1.1294 | 1.92E-08 | 1.94E-07 |
| DOC2B | 25.08702 | 11.49797 | -1.12556 | 1.93E-08 | 1.96E-07 |
| LINC00330 | 0.270411 | 0.073837 | -1.87273 | 2.13E-08 | 2.14E-07 |
| ALDH3A1 | 247.3075 | 69.87317 | -1.8235 | 2.14E-08 | 2.15E-07 |
| AC005064.1 | 0.382318 | 0.078795 | -2.2786 | 2.39E-08 | 2.38E-07 |
| HERC2P6 | 0.554794 | 0.084227 | -2.7196 | 2.40E-08 | 2.39E-07 |
| PDE6B | 1.916505 | 0.728145 | -1.39618 | 2.52E-08 | 2.49E-07 |
| HTR3A | 2.587924 | 0.807443 | -1.68036 | 2.62E-08 | 2.57E-07 |
| CD27 | 16.38786 | 7.712954 | -1.08727 | 2.64E-08 | 2.59E-07 |
| IGHV3-35 | 4.164975 | 1.487342 | -1.48557 | 2.77E-08 | 2.70E-07 |
| IGLC2 | 1404.474 | 535.7858 | -1.3903 | 2.78E-08 | 2.71E-07 |
| NCR3 | 1.341195 | 0.642877 | -1.0609 | 2.99E-08 | 2.88E-07 |
| IGHV3OR16-8 | 3.348338 | 1.118524 | -1.58185 | 3.00E-08 | 2.89E-07 |
| MYO3B | 1.365508 | 2.858639 | 1.06589 | 3.21E-08 | 3.07E-07 |
| LINC00862 | 0.207084 | 0.570846 | 1.462883 | 3.33E-08 | 3.18E-07 |
| IGKC | 3171.584 | 1156.516 | -1.45542 | 3.45E-08 | 3.28E-07 |
| HS3ST6 | 6.117613 | 2.707631 | -1.17594 | 3.52E-08 | 3.34E-07 |
| HS3ST4 | 1.456924 | 0.161113 | -3.17678 | 3.63E-08 | 3.43E-07 |
| LHFPL1 | 0.489363 | 0.128755 | -1.92628 | 3.64E-08 | 3.44E-07 |
| AC010280.1 | 1.911801 | 0.53714 | -1.83156 | 3.74E-08 | 3.52E-07 |
| PRKAG2-AS1 | 2.342901 | 0.953814 | -1.29652 | 4.10E-08 | 3.81E-07 |
| MTND4P20 | 0.479698 | 0.219336 | -1.12899 | 4.27E-08 | 3.96E-07 |
| TUBA5P | 2.316343 | 0.91 | -1.34791 | 4.27E-08 | 3.96E-07 |
| BTNL9 | 4.946856 | 1.063756 | -2.21734 | 4.54E-08 | 4.18E-07 |
| CCER2 | 0.591958 | 0.213447 | -1.47162 | 4.79E-08 | 4.40E-07 |
| LINC01269 | 4.783663 | 2.141621 | -1.15941 | 4.92E-08 | 4.51E-07 |
| DERL3 | 20.2819 | 9.297421 | -1.12529 | 4.93E-08 | 4.52E-07 |
| SVIP | 4.959329 | 2.108996 | -1.23359 | 5.07E-08 | 4.63E-07 |
| WNK2 | 9.135911 | 2.914919 | -1.64809 | 5.10E-08 | 4.65E-07 |
| C4orf19 | 3.408853 | 1.2065 | -1.49846 | 5.35E-08 | 4.83E-07 |
| IGHV3OR16-12 | 1.218923 | 0.336644 | -1.85631 | 5.63E-08 | 5.05E-07 |
| IGKV3D-11 | 13.74053 | 5.006522 | -1.45656 | 5.95E-08 | 5.31E-07 |
| IGLV2-28 | 3.508825 | 0.817138 | -2.10234 | 6.07E-08 | 5.41E-07 |
| KLB | 0.249544 | 0.109951 | -1.18243 | 6.08E-08 | 5.41E-07 |
| SPAG17 | 1.999962 | 0.79462 | -1.33164 | 6.18E-08 | 5.49E-07 |
| FETUB | 13.40421 | 3.793411 | -1.82112 | 6.18E-08 | 5.49E-07 |
| IGLL5 | 96.43478 | 41.67032 | -1.21053 | 6.23E-08 | 5.52E-07 |
| AL021407.2 | 0.36278 | 0.158798 | -1.19191 | 6.39E-08 | 5.65E-07 |
| IGKV2OR22-4 | 5.718298 | 1.72682 | -1.72747 | 6.44E-08 | 5.69E-07 |
| IGLV6-57 | 213.9966 | 66.6239 | -1.68348 | 6.48E-08 | 5.72E-07 |
| FREM1 | 0.404889 | 0.197148 | -1.03825 | 6.59E-08 | 5.80E-07 |
| CRNN | 273.7542 | 25.15799 | -3.44379 | 6.87E-08 | 6.03E-07 |
| FCRL5 | 1.407505 | 0.593257 | -1.24641 | 6.91E-08 | 6.06E-07 |
| AC136621.1 | 0.67853 | 1.459699 | 1.105186 | 6.99E-08 | 6.12E-07 |
| ASTL | 0.5506 | 0.25755 | -1.09615 | 7.42E-08 | 6.48E-07 |
| AP003108.5 | 0.237303 | 0.493259 | 1.055618 | 7.46E-08 | 6.51E-07 |
| AC106795.2 | 2.013308 | 0.872879 | -1.20571 | 7.58E-08 | 6.60E-07 |
| RPRM | 3.099894 | 1.399444 | -1.14736 | 7.77E-08 | 6.76E-07 |
| AL109936.2 | 1.076352 | 0.452108 | -1.25141 | 7.85E-08 | 6.82E-07 |
| DNASE1L3 | 8.052818 | 4.026181 | -1.00008 | 7.95E-08 | 6.90E-07 |
| PPIAP39 | 0.617148 | 0.25702 | -1.26374 | 8.02E-08 | 6.96E-07 |
| XCR1 | 1.465198 | 0.693736 | -1.07864 | 8.07E-08 | 6.99E-07 |
| MEI1 | 5.925944 | 1.40561 | -2.07585 | 8.14E-08 | 7.03E-07 |
| SPRR2A | 2488.625 | 993.7218 | -1.32443 | 8.17E-08 | 7.05E-07 |
| MUC20 | 13.09836 | 5.368886 | -1.28669 | 8.27E-08 | 7.12E-07 |
| CGNL1 | 3.570545 | 1.451833 | -1.29827 | 8.74E-08 | 7.49E-07 |
| AC080013.4 | 0.42744 | 0.207487 | -1.0427 | 9.15E-08 | 7.81E-07 |
| TFAP2B | 0.278699 | 0.058287 | -2.25745 | 9.74E-08 | 8.24E-07 |
| AFF3 | 0.445613 | 0.159266 | -1.48435 | 9.96E-08 | 8.41E-07 |
| JCHAIN | 273.2768 | 96.42026 | -1.50295 | 1.01E-07 | 8.51E-07 |
| SH3BGRL2 | 10.67242 | 5.238061 | -1.02678 | 1.02E-07 | 8.54E-07 |
| LINC00885 | 5.289709 | 2.466526 | -1.10071 | 1.02E-07 | 8.59E-07 |
| IGHG3 | 664.2542 | 244.4984 | -1.44191 | 1.06E-07 | 8.84E-07 |
| CYP26A1 | 8.591642 | 2.8072 | -1.6138 | 1.07E-07 | 8.97E-07 |
| SLC9A4 | 1.330856 | 0.169569 | -2.97241 | 1.08E-07 | 9.04E-07 |
| PNMA3 | 1.076572 | 0.295936 | -1.86309 | 1.09E-07 | 9.06E-07 |
| LINC00992 | 1.264006 | 0.435515 | -1.53721 | 1.11E-07 | 9.24E-07 |
| KIF25 | 0.441576 | 0.127342 | -1.79396 | 1.13E-07 | 9.43E-07 |
| IGLV2-18 | 28.01192 | 9.111147 | -1.62034 | 1.13E-07 | 9.43E-07 |
| PTX3 | 4.535623 | 10.42499 | 1.200674 | 1.16E-07 | 9.63E-07 |
| FAM184A | 0.590284 | 0.268295 | -1.13759 | 1.18E-07 | 9.78E-07 |
| IGHA1 | 4301.426 | 1520.032 | -1.50071 | 1.21E-07 | 1.00E-06 |
| ANKRD36BP2 | 0.767951 | 0.295933 | -1.37574 | 1.23E-07 | 1.02E-06 |
| AC068338.3 | 1.079864 | 0.534972 | -1.01331 | 1.28E-07 | 1.05E-06 |
| AL122058.1 | 0.520352 | 0.166433 | -1.64455 | 1.36E-07 | 1.11E-06 |
| LINC00861 | 0.518612 | 0.218997 | -1.24375 | 1.36E-07 | 1.11E-06 |
| ABCA3 | 11.34745 | 2.175256 | -2.38311 | 1.37E-07 | 1.12E-06 |
| ACER1 | 3.742324 | 1.265488 | -1.56424 | 1.39E-07 | 1.13E-06 |
| SOX2 | 118.977 | 44.57661 | -1.41632 | 1.39E-07 | 1.13E-06 |
| KEL | 2.205685 | 0.142328 | -3.95393 | 1.40E-07 | 1.14E-06 |
| IL36A | 31.92892 | 5.957775 | -2.42202 | 1.41E-07 | 1.14E-06 |
| Z85994.1 | 0.233763 | 0.575338 | 1.299367 | 1.47E-07 | 1.19E-06 |
| AC068587.2 | 1.153158 | 0.482698 | -1.2564 | 1.48E-07 | 1.20E-06 |
| TGM7 | 0.321783 | 0.075638 | -2.08891 | 1.51E-07 | 1.22E-06 |
| COLEC10 | 0.281512 | 0.114053 | -1.30349 | 1.54E-07 | 1.24E-06 |
| IGLV1-50 | 4.176296 | 1.565716 | -1.4154 | 1.56E-07 | 1.26E-06 |
| ITGAD | 0.293618 | 0.138826 | -1.08066 | 1.61E-07 | 1.29E-06 |
| SLC16A11 | 2.251883 | 1.044644 | -1.10812 | 1.64E-07 | 1.31E-06 |
| IGLV3-9 | 56.45692 | 24.98977 | -1.17581 | 1.64E-07 | 1.32E-06 |
| LINC01475 | 0.834296 | 0.243139 | -1.77878 | 1.65E-07 | 1.32E-06 |
| SPRR2F | 185.8687 | 59.97137 | -1.63194 | 1.67E-07 | 1.34E-06 |
| ADCY5 | 1.895657 | 0.722099 | -1.39243 | 1.68E-07 | 1.35E-06 |
| GPR18 | 1.159568 | 0.544594 | -1.09034 | 1.75E-07 | 1.39E-06 |
| SAMD12 | 6.234268 | 2.853042 | -1.12772 | 1.75E-07 | 1.39E-06 |
| TRDV1 | 1.090589 | 0.367606 | -1.56887 | 1.79E-07 | 1.42E-06 |
| ACTG1P25 | 0.334154 | 0.158481 | -1.0762 | 1.81E-07 | 1.44E-06 |
| IGKV3-11 | 529.8489 | 203.5544 | -1.38017 | 1.83E-07 | 1.45E-06 |
| CHDH | 1.916333 | 0.67368 | -1.50821 | 1.87E-07 | 1.48E-06 |
| IGKV3-7 | 13.83705 | 5.924813 | -1.2237 | 1.89E-07 | 1.49E-06 |
| TRBV12-3 | 0.766825 | 0.338839 | -1.1783 | 1.93E-07 | 1.52E-06 |
| STC1 | 20.7037 | 42.59548 | 1.040812 | 1.94E-07 | 1.53E-06 |
| SMIM10L2A | 2.059574 | 0.907142 | -1.18295 | 1.94E-07 | 1.53E-06 |
| AC091564.6 | 0.251702 | 0.512578 | 1.026055 | 2.00E-07 | 1.57E-06 |
| LINC00582 | 0.68086 | 0.263767 | -1.3681 | 2.04E-07 | 1.60E-06 |
| AADACL2 | 1.197183 | 0.340226 | -1.81508 | 2.12E-07 | 1.65E-06 |
| TMPRSS11E | 89.40815 | 32.52501 | -1.45886 | 2.14E-07 | 1.66E-06 |
| KCNH1-IT1 | 0.35069 | 0.083767 | -2.06574 | 2.14E-07 | 1.66E-06 |
| IGKV1-12 | 8.848119 | 3.407164 | -1.3768 | 2.15E-07 | 1.67E-06 |
| IGHV3-72 | 28.24787 | 11.57725 | -1.28685 | 2.17E-07 | 1.68E-06 |
| CRABP1 | 15.68838 | 2.311704 | -2.76267 | 2.20E-07 | 1.70E-06 |
| RFPL1S | 0.371212 | 0.052489 | -2.82217 | 2.21E-07 | 1.71E-06 |
| LINC01907 | 0.227597 | 0.480282 | 1.077399 | 2.22E-07 | 1.72E-06 |
| IGKV1-17 | 197.4331 | 43.6489 | -2.17735 | 2.24E-07 | 1.73E-06 |
| IGKV2-30 | 14.25759 | 4.877499 | -1.54752 | 2.29E-07 | 1.77E-06 |
| CRYGS | 2.470692 | 1.213406 | -1.02585 | 2.40E-07 | 1.84E-06 |
| SCML4 | 0.465893 | 0.217114 | -1.10155 | 2.41E-07 | 1.85E-06 |
| IGHV3-38 | 3.003581 | 1.175086 | -1.35392 | 2.51E-07 | 1.92E-06 |
| UGT1A7 | 8.606016 | 4.281134 | -1.00735 | 2.53E-07 | 1.93E-06 |
| RHCG | 531.2484 | 192.3488 | -1.46566 | 2.57E-07 | 1.96E-06 |
| LIPH | 12.59107 | 5.456662 | -1.20631 | 2.57E-07 | 1.96E-06 |
| AC084816.1 | 0.377391 | 0.092715 | -2.02518 | 2.64E-07 | 2.00E-06 |
| AC008649.1 | 1.76527 | 0.761282 | -1.21339 | 2.65E-07 | 2.01E-06 |
| AC092681.1 | 0.405381 | 0.12006 | -1.75552 | 2.66E-07 | 2.01E-06 |
| DTNB-AS1 | 1.313249 | 0.535343 | -1.2946 | 2.75E-07 | 2.08E-06 |
| DEFB1 | 177.7617 | 85.41928 | -1.05731 | 2.87E-07 | 2.16E-06 |
| LINC02036 | 0.630475 | 0.174644 | -1.85202 | 2.94E-07 | 2.20E-06 |
| S100A8 | 7775.44 | 3721.495 | -1.06304 | 2.97E-07 | 2.22E-06 |
| AC138904.1 | 0.592304 | 0.159291 | -1.89467 | 3.01E-07 | 2.25E-06 |
| IGHV1OR15-9 | 8.683773 | 3.409844 | -1.34862 | 3.06E-07 | 2.28E-06 |
| AC063948.1 | 1.587641 | 0.778024 | -1.029 | 3.06E-07 | 2.28E-06 |
| OXER1 | 2.325465 | 1.089958 | -1.09325 | 3.06E-07 | 2.28E-06 |
| LINC01522 | 0.544933 | 1.202898 | 1.142364 | 3.07E-07 | 2.28E-06 |
| ELF5 | 2.856078 | 1.115482 | -1.35637 | 3.08E-07 | 2.29E-06 |
| KCNJ11 | 2.459325 | 1.053801 | -1.22266 | 3.14E-07 | 2.33E-06 |
| IGKV2-28 | 9.037627 | 1.635342 | -2.46635 | 3.23E-07 | 2.39E-06 |
| IGLV1-51 | 410.5135 | 152.8757 | -1.42507 | 3.27E-07 | 2.42E-06 |
| AC115284.2 | 0.824522 | 0.349323 | -1.239 | 3.29E-07 | 2.43E-06 |
| IGKV1OR22-1 | 3.29036 | 0.918085 | -1.84155 | 3.32E-07 | 2.45E-06 |
| DELEC1 | 1.03156 | 2.418592 | 1.22934 | 3.47E-07 | 2.55E-06 |
| IGHV3-7 | 9.37716 | 3.071871 | -1.61003 | 3.47E-07 | 2.55E-06 |
| HEPHL1 | 49.31706 | 117.2437 | 1.249352 | 3.64E-07 | 2.65E-06 |
| KCNJ10 | 0.4574 | 0.208821 | -1.13119 | 3.65E-07 | 2.66E-06 |
| MGAT3 | 8.213654 | 1.593002 | -2.36628 | 3.70E-07 | 2.70E-06 |
| AC006262.4 | 1.956695 | 0.443346 | -2.14191 | 3.79E-07 | 2.75E-06 |
| AC025580.1 | 0.609305 | 0.203452 | -1.58248 | 3.86E-07 | 2.80E-06 |
| IGKV1D-27 | 4.909151 | 1.980623 | -1.30952 | 3.88E-07 | 2.81E-06 |
| FMO3 | 9.883182 | 1.962206 | -2.3325 | 3.94E-07 | 2.84E-06 |
| IGHGP | 204.0662 | 86.04963 | -1.2458 | 3.99E-07 | 2.88E-06 |
| GABRP | 29.45776 | 9.169001 | -1.68381 | 4.01E-07 | 2.89E-06 |
| IGHV3-47 | 3.49422 | 1.430068 | -1.28889 | 4.10E-07 | 2.95E-06 |
| TRBJ2-1 | 3.061436 | 1.480667 | -1.04796 | 4.12E-07 | 2.95E-06 |
| AL356740.1 | 0.532449 | 0.148934 | -1.83797 | 4.15E-07 | 2.97E-06 |
| IGHJ3 | 88.40088 | 32.56121 | -1.44091 | 4.18E-07 | 2.99E-06 |
| MFSD4A | 5.480156 | 1.868081 | -1.55266 | 4.20E-07 | 3.00E-06 |
| AC016705.2 | 0.605673 | 0.090589 | -2.74113 | 4.21E-07 | 3.01E-06 |
| LINC00589 | 0.161494 | 0.44718 | 1.469374 | 4.23E-07 | 3.02E-06 |
| AC243829.2 | 0.533925 | 0.133904 | -1.99543 | 4.33E-07 | 3.09E-06 |
| PARM1 | 23.27189 | 6.178351 | -1.91329 | 4.39E-07 | 3.12E-06 |
| LINC00601 | 0.243474 | 0.51667 | 1.085475 | 4.52E-07 | 3.20E-06 |
| MYB | 4.278431 | 1.654718 | -1.3705 | 4.56E-07 | 3.23E-06 |
| AC093159.1 | 2.29061 | 0.760689 | -1.59035 | 4.59E-07 | 3.25E-06 |
| IGHV3-75 | 1.13654 | 0.400191 | -1.50589 | 4.67E-07 | 3.30E-06 |
| CLEC4O | 0.940277 | 2.345311 | 1.318622 | 4.67E-07 | 3.30E-06 |
| IGHV3-25 | 1.228405 | 0.392568 | -1.64577 | 4.75E-07 | 3.35E-06 |
| CXCL3 | 4.630639 | 10.77898 | 1.218938 | 4.86E-07 | 3.42E-06 |
| CYP4X1 | 14.70451 | 4.401887 | -1.74006 | 4.91E-07 | 3.45E-06 |
| COL6A5 | 0.280065 | 0.137184 | -1.02965 | 4.93E-07 | 3.47E-06 |
| RSPO1 | 0.627628 | 0.298547 | -1.07195 | 4.95E-07 | 3.48E-06 |
| IFNWP2 | 0.178209 | 0.450276 | 1.337237 | 5.03E-07 | 3.53E-06 |
| IGLL3P | 0.886459 | 0.202414 | -2.13074 | 5.03E-07 | 3.53E-06 |
| RNF225 | 3.589069 | 1.670885 | -1.103 | 5.10E-07 | 3.57E-06 |
| CCDC185 | 0.279159 | 0.09521 | -1.55191 | 5.22E-07 | 3.65E-06 |
| IGKV1-16 | 142.0441 | 60.26176 | -1.23702 | 5.31E-07 | 3.70E-06 |
| LINC02576 | 0.594504 | 0.276216 | -1.10589 | 5.49E-07 | 3.82E-06 |
| IGLV3-32 | 0.672224 | 0.242585 | -1.47045 | 5.58E-07 | 3.88E-06 |
| SERPINB12 | 8.53434 | 1.797534 | -2.24726 | 5.66E-07 | 3.94E-06 |
| UPK3A | 0.434306 | 0.190632 | -1.18792 | 5.71E-07 | 3.97E-06 |
| ZNF541 | 2.597061 | 0.712611 | -1.86569 | 5.75E-07 | 3.99E-06 |
| MESP1 | 1.066236 | 0.374739 | -1.50857 | 5.84E-07 | 4.04E-06 |
| THEMIS | 1.44073 | 0.621657 | -1.21261 | 5.91E-07 | 4.08E-06 |
| TRAV18 | 0.243971 | 0.105444 | -1.21024 | 6.01E-07 | 4.15E-06 |
| NSUN7 | 1.651162 | 0.757987 | -1.12324 | 6.10E-07 | 4.20E-06 |
| KRT24 | 54.13649 | 10.5377 | -2.36104 | 6.15E-07 | 4.23E-06 |
| BCL2 | 6.476202 | 2.633408 | -1.29822 | 6.26E-07 | 4.30E-06 |
| AC006026.3 | 1.418515 | 0.673421 | -1.0748 | 6.29E-07 | 4.32E-06 |
| AL645939.2 | 0.216017 | 0.508887 | 1.236203 | 6.31E-07 | 4.33E-06 |
| SERPINB11 | 4.793818 | 1.768526 | -1.43863 | 6.33E-07 | 4.34E-06 |
| OXGR1 | 1.587855 | 0.519602 | -1.6116 | 6.33E-07 | 4.34E-06 |
| IGHV3-53 | 40.05308 | 15.32565 | -1.38597 | 6.33E-07 | 4.34E-06 |
| IGHV3-63 | 3.988171 | 1.296299 | -1.62133 | 6.40E-07 | 4.38E-06 |
| IGHG2 | 2192.239 | 920.4712 | -1.25196 | 6.47E-07 | 4.42E-06 |
| IGLV3-12 | 5.386659 | 1.414483 | -1.92912 | 6.47E-07 | 4.43E-06 |
| TRBV7-6 | 1.365805 | 0.471056 | -1.53578 | 6.48E-07 | 4.43E-06 |
| ALDH1A1 | 98.83481 | 36.71201 | -1.42877 | 6.76E-07 | 4.60E-06 |
| ZNF683 | 3.701401 | 1.557551 | -1.24879 | 6.78E-07 | 4.60E-06 |
| Z97653.1 | 0.671681 | 0.286747 | -1.228 | 7.03E-07 | 4.75E-06 |
| UPB1 | 0.66646 | 0.140066 | -2.25041 | 7.24E-07 | 4.87E-06 |
| C15orf56 | 0.367722 | 0.167065 | -1.13821 | 7.40E-07 | 4.97E-06 |
| KLRK1 | 0.365118 | 0.180627 | -1.01535 | 7.54E-07 | 5.04E-06 |
| IGLC6 | 4.502303 | 1.669424 | -1.43131 | 7.57E-07 | 5.06E-06 |
| A2ML1 | 139.3795 | 66.58204 | -1.06581 | 7.58E-07 | 5.06E-06 |
| IGKV4-1 | 687.252 | 323.6393 | -1.08645 | 7.58E-07 | 5.06E-06 |
| NEXMIF | 0.393742 | 0.120551 | -1.70761 | 7.60E-07 | 5.07E-06 |
| NKX2-3 | 3.071665 | 0.42791 | -2.84364 | 7.69E-07 | 5.13E-06 |
| GSTA1 | 68.87108 | 22.8925 | -1.58902 | 7.75E-07 | 5.17E-06 |
| IGHV3-74 | 104.465 | 40.1068 | -1.3811 | 7.79E-07 | 5.19E-06 |
| ALOXE3P1 | 7.203388 | 2.445199 | -1.55872 | 7.81E-07 | 5.20E-06 |
| NMU | 39.858 | 16.96263 | -1.23251 | 7.84E-07 | 5.21E-06 |
| IGLV1-47 | 229.8149 | 92.00962 | -1.32062 | 7.87E-07 | 5.23E-06 |
| AC104971.3 | 0.44023 | 0.217155 | -1.01953 | 7.88E-07 | 5.23E-06 |
| TLR10 | 1.469721 | 0.519382 | -1.50067 | 7.93E-07 | 5.26E-06 |
| TRBV11-2 | 1.431991 | 0.5946 | -1.26803 | 8.21E-07 | 5.43E-06 |
| CAPN14 | 8.497307 | 2.661619 | -1.6747 | 8.44E-07 | 5.56E-06 |
| BMP3 | 1.377945 | 0.345301 | -1.99659 | 8.61E-07 | 5.66E-06 |
| IGHV3-37 | 0.755084 | 0.257794 | -1.55042 | 8.87E-07 | 5.82E-06 |
| AL772337.3 | 0.272647 | 0.602553 | 1.144055 | 9.12E-07 | 5.97E-06 |
| IGHG1 | 2384.55 | 1002.423 | -1.25023 | 9.18E-07 | 6.01E-06 |
| SPRR1A | 5772.646 | 2748.986 | -1.07033 | 9.31E-07 | 6.08E-06 |
| IGLV1-44 | 275.2971 | 128.2235 | -1.10233 | 9.45E-07 | 6.16E-06 |
| AP001360.1 | 2.405921 | 0.311083 | -2.95122 | 9.54E-07 | 6.22E-06 |
| GZMH | 17.00499 | 8.129614 | -1.0647 | 9.66E-07 | 6.28E-06 |
| AC100791.2 | 0.412571 | 0.151936 | -1.44118 | 9.70E-07 | 6.30E-06 |
| PCSK2 | 0.907752 | 0.025025 | -5.18084 | 9.72E-07 | 6.31E-06 |
| TACR1 | 0.620192 | 0.297282 | -1.06088 | 9.80E-07 | 6.36E-06 |
| IGLL1 | 0.76462 | 0.193413 | -1.98306 | 9.90E-07 | 6.41E-06 |
| IGHV5-78 | 4.632175 | 1.537286 | -1.5913 | 1.01E-06 | 6.51E-06 |
| MIXL1 | 0.941673 | 0.38882 | -1.27612 | 1.01E-06 | 6.52E-06 |
| ZNF662 | 1.320945 | 0.635088 | -1.05654 | 1.01E-06 | 6.54E-06 |
| IGHV3-60 | 3.170719 | 0.950846 | -1.73753 | 1.03E-06 | 6.66E-06 |
| UBASH3A | 2.242174 | 1.1094 | -1.01512 | 1.04E-06 | 6.68E-06 |
| DSG1-AS1 | 4.239777 | 1.996666 | -1.0864 | 1.04E-06 | 6.70E-06 |
| IGHV4-59 | 210.3083 | 80.30619 | -1.38892 | 1.07E-06 | 6.85E-06 |
| CCDC60 | 0.681824 | 0.202227 | -1.75342 | 1.08E-06 | 6.93E-06 |
| YWHAEP3 | 0.251062 | 0.565363 | 1.171134 | 1.09E-06 | 6.97E-06 |
| ACSL6 | 0.245741 | 0.104365 | -1.23551 | 1.09E-06 | 6.99E-06 |
| AF131215.7 | 1.205957 | 0.54274 | -1.15185 | 1.10E-06 | 7.02E-06 |
| LRRK2-DT | 0.305427 | 0.149253 | -1.03307 | 1.13E-06 | 7.18E-06 |
| AC103563.1 | 5.10225 | 1.943554 | -1.39244 | 1.13E-06 | 7.22E-06 |
| COPDA1 | 0.723297 | 0.320409 | -1.17467 | 1.15E-06 | 7.30E-06 |
| IGKV2OR2-1 | 1.527944 | 0.427736 | -1.8368 | 1.16E-06 | 7.40E-06 |
| FA2H | 5.502586 | 2.32699 | -1.24164 | 1.17E-06 | 7.41E-06 |
| IGKV3D-15 | 15.51788 | 5.868948 | -1.40276 | 1.19E-06 | 7.56E-06 |
| AC034223.1 | 0.647661 | 1.665948 | 1.363033 | 1.22E-06 | 7.71E-06 |
| PRR36 | 2.053442 | 0.656659 | -1.64483 | 1.23E-06 | 7.74E-06 |
| AC023983.2 | 0.507595 | 0.204431 | -1.31207 | 1.23E-06 | 7.75E-06 |
| TMEM211 | 0.73242 | 0.202076 | -1.85777 | 1.23E-06 | 7.77E-06 |
| IGHV3OR16-6 | 2.270201 | 0.540691 | -2.06994 | 1.26E-06 | 7.91E-06 |
| SYNPR-AS1 | 1.008777 | 0.343706 | -1.55336 | 1.28E-06 | 8.06E-06 |
| IGLV3-10 | 159.313 | 58.70346 | -1.44035 | 1.30E-06 | 8.14E-06 |
| NTRK2 | 30.29344 | 14.60438 | -1.0526 | 1.31E-06 | 8.17E-06 |
| FTH1P22 | 1.984818 | 0.97825 | -1.02073 | 1.31E-06 | 8.20E-06 |
| IGLV3-1 | 215.3081 | 79.14792 | -1.44378 | 1.34E-06 | 8.38E-06 |
| Z99916.1 | 0.493567 | 0.209847 | -1.23391 | 1.35E-06 | 8.40E-06 |
| YPEL1 | 0.990437 | 0.4171 | -1.24767 | 1.37E-06 | 8.51E-06 |
| IGHV3OR15-7 | 4.578142 | 1.847131 | -1.30948 | 1.38E-06 | 8.57E-06 |
| ATP10B | 5.164839 | 2.3217 | -1.15354 | 1.42E-06 | 8.77E-06 |
| TRBV5-1 | 2.513903 | 1.131115 | -1.15218 | 1.42E-06 | 8.80E-06 |
| CCL25 | 0.515954 | 0.130295 | -1.98546 | 1.43E-06 | 8.85E-06 |
| HOXB1 | 0.302512 | 0.145288 | -1.05808 | 1.43E-06 | 8.85E-06 |
| AL162274.1 | 0.23412 | 0.52014 | 1.151651 | 1.45E-06 | 8.95E-06 |
| AC020907.1 | 3.572941 | 1.085992 | -1.7181 | 1.51E-06 | 9.26E-06 |
| CFAP91 | 0.384125 | 0.093025 | -2.04588 | 1.51E-06 | 9.27E-06 |
| AC131649.2 | 0.685848 | 0.33611 | -1.02896 | 1.57E-06 | 9.60E-06 |
| IGKV6-21 | 25.07639 | 6.497671 | -1.94833 | 1.58E-06 | 9.66E-06 |
| CEP126 | 0.985644 | 2.309219 | 1.228267 | 1.60E-06 | 9.74E-06 |
| AL139393.1 | 0.571065 | 0.256908 | -1.1524 | 1.62E-06 | 9.86E-06 |
| IGHV1OR15-2 | 12.60053 | 5.532446 | -1.18749 | 1.63E-06 | 9.88E-06 |
| IGKV1-39 | 11.68059 | 3.158439 | -1.88683 | 1.64E-06 | 9.96E-06 |
| CTTNBP2 | 1.777366 | 0.746949 | -1.25066 | 1.64E-06 | 9.96E-06 |
| IGLV5-37 | 10.13992 | 2.776213 | -1.86886 | 1.73E-06 | 1.05E-05 |
| AC034105.1 | 2.905141 | 0.967795 | -1.58583 | 1.74E-06 | 1.05E-05 |
| IGHV7-81 | 3.388819 | 0.863608 | -1.97233 | 1.74E-06 | 1.05E-05 |
| AL391056.1 | 1.100174 | 0.377162 | -1.54448 | 1.78E-06 | 1.07E-05 |
| IGKV1-5 | 625.2229 | 241.9449 | -1.36969 | 1.80E-06 | 1.08E-05 |
| IGKV1-27 | 115.0444 | 49.53421 | -1.21569 | 1.81E-06 | 1.08E-05 |
| LINC02158 | 1.429382 | 0.559409 | -1.35342 | 1.83E-06 | 1.09E-05 |
| SNX31 | 2.857053 | 1.009558 | -1.5008 | 1.83E-06 | 1.10E-05 |
| CNR2 | 0.260597 | 0.105261 | -1.30785 | 1.86E-06 | 1.11E-05 |
| IGHV7-56 | 2.14208 | 0.795492 | -1.42909 | 1.90E-06 | 1.13E-05 |
| IGHV3-71 | 4.440162 | 1.691746 | -1.3921 | 2.00E-06 | 1.18E-05 |
| IGLV3-19 | 411.0284 | 175.3082 | -1.22934 | 2.02E-06 | 1.19E-05 |
| KIAA0895LP1 | 0.497747 | 0.164907 | -1.59376 | 2.02E-06 | 1.20E-05 |
| AC009560.1 | 0.555907 | 0.262767 | -1.08106 | 2.04E-06 | 1.20E-05 |
| CXCL5 | 7.768507 | 27.19499 | 1.807632 | 2.06E-06 | 1.22E-05 |
| FAM222A | 5.033378 | 2.511795 | -1.00281 | 2.06E-06 | 1.22E-05 |
| ANKRD34B | 0.669865 | 0.108677 | -2.62382 | 2.06E-06 | 1.22E-05 |
| ATP12A | 14.06432 | 5.024819 | -1.4849 | 2.12E-06 | 1.25E-05 |
| IGHV7-27 | 1.712736 | 0.756697 | -1.17851 | 2.15E-06 | 1.27E-05 |
| AC006042.1 | 0.850649 | 0.396992 | -1.09945 | 2.17E-06 | 1.28E-05 |
| IGHV4-55 | 17.08585 | 7.162737 | -1.25422 | 2.23E-06 | 1.31E-05 |
| PNOC | 1.391745 | 0.584518 | -1.25157 | 2.24E-06 | 1.31E-05 |
| IGKV2-26 | 1.377249 | 0.517393 | -1.41246 | 2.25E-06 | 1.32E-05 |
| IGLV3-16 | 10.68683 | 2.923414 | -1.87011 | 2.26E-06 | 1.32E-05 |
| IGLJ2 | 3.183134 | 1.186332 | -1.42394 | 2.26E-06 | 1.32E-05 |
| IGHG4 | 1398.119 | 576.1545 | -1.27896 | 2.28E-06 | 1.33E-05 |
| AC011483.3 | 0.348324 | 0.098764 | -1.81836 | 2.32E-06 | 1.35E-05 |
| AC138207.3 | 1.957418 | 0.914258 | -1.09828 | 2.45E-06 | 1.42E-05 |
| IGHV6-1 | 10.05943 | 4.114361 | -1.28981 | 2.47E-06 | 1.43E-05 |
| CNKSR2 | 0.306192 | 0.110175 | -1.47463 | 2.50E-06 | 1.44E-05 |
| WDR49 | 0.711574 | 0.205297 | -1.7933 | 2.52E-06 | 1.45E-05 |
| CRB2 | 1.036624 | 0.208678 | -2.31254 | 2.54E-06 | 1.47E-05 |
| TESC | 4.257791 | 1.673683 | -1.34708 | 2.55E-06 | 1.47E-05 |
| RPTN | 23.05559 | 6.234063 | -1.88687 | 2.57E-06 | 1.48E-05 |
| IGKV1-33 | 4.34656 | 1.8028 | -1.26963 | 2.58E-06 | 1.48E-05 |
| FAM189A2 | 3.18466 | 1.351945 | -1.2361 | 2.60E-06 | 1.49E-05 |
| IGKV2D-29 | 46.41492 | 17.77004 | -1.38514 | 2.68E-06 | 1.54E-05 |
| DISP3 | 0.471829 | 1.804818 | 1.935517 | 2.71E-06 | 1.55E-05 |
| PRKAR2B | 4.488951 | 2.034059 | -1.14202 | 2.71E-06 | 1.55E-05 |
| COCH | 4.336582 | 1.79268 | -1.27444 | 2.75E-06 | 1.57E-05 |
| IGKV3OR2-268 | 7.571133 | 3.003418 | -1.3339 | 2.76E-06 | 1.58E-05 |
| AL356234.2 | 3.684616 | 1.520056 | -1.27739 | 2.78E-06 | 1.59E-05 |
| RIMKLA | 0.603353 | 0.223235 | -1.43444 | 2.84E-06 | 1.62E-05 |
| MYRIP | 0.363754 | 0.161874 | -1.16809 | 2.84E-06 | 1.62E-05 |
| AL365361.1 | 1.519958 | 0.655655 | -1.21302 | 2.84E-06 | 1.62E-05 |
| STXBP6 | 1.625992 | 0.553173 | -1.55552 | 2.89E-06 | 1.65E-05 |
| IGHA2 | 999.0524 | 364.9637 | -1.45281 | 2.96E-06 | 1.68E-05 |
| AC103563.2 | 0.568664 | 0.102512 | -2.47179 | 2.97E-06 | 1.68E-05 |
| IGHV4OR15-8 | 0.907272 | 0.238019 | -1.93046 | 3.00E-06 | 1.69E-05 |
| HAMP | 0.679864 | 0.336283 | -1.01557 | 3.02E-06 | 1.70E-05 |
| IGHV3-15 | 224.2394 | 81.77877 | -1.45524 | 3.05E-06 | 1.72E-05 |
| IGHV3-65 | 0.806511 | 0.188819 | -2.09469 | 3.09E-06 | 1.74E-05 |
| IGHV1-18 | 398.7972 | 157.3569 | -1.34161 | 3.18E-06 | 1.78E-05 |
| IGLV2-14 | 508.6005 | 182.1466 | -1.48143 | 3.18E-06 | 1.78E-05 |
| CRYM | 1.659143 | 0.55933 | -1.56867 | 3.24E-06 | 1.81E-05 |
| ZFHX2 | 0.505591 | 0.214753 | -1.23529 | 3.26E-06 | 1.82E-05 |
| TRBV19 | 3.161625 | 1.41739 | -1.15743 | 3.28E-06 | 1.83E-05 |
| CYP4F3 | 13.77188 | 3.379533 | -2.02683 | 3.30E-06 | 1.85E-05 |
| REELD1 | 0.305734 | 0.125888 | -1.28013 | 3.36E-06 | 1.87E-05 |
| AL390728.3 | 0.477484 | 0.229823 | -1.05493 | 3.50E-06 | 1.94E-05 |
| IGLV1-40 | 458.0933 | 184.5105 | -1.31194 | 3.60E-06 | 1.99E-05 |
| ARG1 | 5.996918 | 1.914195 | -1.64748 | 3.60E-06 | 2.00E-05 |
| TRAT1 | 1.549304 | 0.663929 | -1.22252 | 3.62E-06 | 2.00E-05 |
| CLLU1-AS1 | 0.859887 | 0.396563 | -1.1166 | 3.66E-06 | 2.02E-05 |
| BEND5 | 2.117408 | 0.931581 | -1.18455 | 3.69E-06 | 2.04E-05 |
| LINC02038 | 0.305545 | 0.062371 | -2.29244 | 3.72E-06 | 2.05E-05 |
| ABCD2 | 0.420513 | 0.203367 | -1.04806 | 3.76E-06 | 2.07E-05 |
| IGFBP1 | 0.157296 | 0.389657 | 1.308721 | 3.78E-06 | 2.08E-05 |
| LINC02487 | 2.425711 | 0.372067 | -2.70477 | 3.79E-06 | 2.08E-05 |
| B3GALT5 | 1.523564 | 0.342897 | -2.1516 | 3.83E-06 | 2.10E-05 |
| IL2RG | 47.23357 | 21.95024 | -1.10558 | 3.90E-06 | 2.14E-05 |
| AC100793.4 | 0.566338 | 0.200995 | -1.4945 | 3.93E-06 | 2.15E-05 |
| GLS2 | 0.890173 | 0.313518 | -1.50554 | 4.03E-06 | 2.20E-05 |
| IGHV3-6 | 0.665731 | 0.189541 | -1.81243 | 4.07E-06 | 2.22E-05 |
| CD40LG | 1.136544 | 0.466297 | -1.28533 | 4.07E-06 | 2.22E-05 |
| IGHJ1 | 15.41476 | 5.2891 | -1.54322 | 4.10E-06 | 2.23E-05 |
| PDCL2 | 0.418401 | 0.122483 | -1.7723 | 4.32E-06 | 2.34E-05 |
| TRAV22 | 0.727567 | 0.342817 | -1.08564 | 4.35E-06 | 2.35E-05 |
| LINC01018 | 0.971355 | 0.285444 | -1.76679 | 4.39E-06 | 2.37E-05 |
| IGLV2-23 | 346.236 | 124.254 | -1.47846 | 4.40E-06 | 2.37E-05 |
| MUC20P1 | 4.345077 | 1.431289 | -1.60207 | 4.42E-06 | 2.38E-05 |
| WSCD2 | 1.366696 | 0.476972 | -1.51872 | 4.42E-06 | 2.38E-05 |
| IGLV2-5 | 1.864735 | 0.56712 | -1.71725 | 4.46E-06 | 2.40E-05 |
| IGHM | 406.5661 | 97.81781 | -2.05532 | 4.49E-06 | 2.41E-05 |
| CHL1 | 6.256287 | 2.855444 | -1.13159 | 4.61E-06 | 2.47E-05 |
| IGHV3-23 | 438.9098 | 179.2197 | -1.2922 | 4.71E-06 | 2.51E-05 |
| AC104461.1 | 0.201065 | 0.410091 | 1.028279 | 4.75E-06 | 2.53E-05 |
| AC011944.1 | 0.220832 | 0.476276 | 1.108849 | 4.93E-06 | 2.62E-05 |
| MIR8071-1 | 4.482319 | 1.977218 | -1.18077 | 4.96E-06 | 2.63E-05 |
| CACNA1B | 1.863975 | 0.69933 | -1.41434 | 4.96E-06 | 2.63E-05 |
| FOXD3-AS1 | 3.980202 | 1.373974 | -1.53449 | 5.07E-06 | 2.68E-05 |
| IGKV1-6 | 106.0374 | 43.2657 | -1.29328 | 5.10E-06 | 2.70E-05 |
| IGKV3-20 | 961.8982 | 368.9654 | -1.3824 | 5.14E-06 | 2.71E-05 |
| CYP2F1 | 0.41284 | 0.197724 | -1.0621 | 5.17E-06 | 2.73E-05 |
| IGHV3-62 | 2.16426 | 0.776974 | -1.47794 | 5.20E-06 | 2.74E-05 |
| IGHV1-24 | 172.9038 | 85.16408 | -1.02165 | 5.24E-06 | 2.76E-05 |
| AC093458.2 | 0.336315 | 0.132146 | -1.34768 | 5.25E-06 | 2.76E-05 |
| FOXD3 | 1.711502 | 0.483346 | -1.82413 | 5.29E-06 | 2.78E-05 |
| IGHV3-79 | 0.693727 | 0.268032 | -1.37196 | 5.43E-06 | 2.85E-05 |
| ATRNL1 | 0.478051 | 0.204205 | -1.22715 | 5.47E-06 | 2.87E-05 |
| KRT40 | 0.331666 | 0.082686 | -2.00401 | 5.59E-06 | 2.92E-05 |
| NR3C2 | 1.401101 | 0.516116 | -1.44079 | 5.59E-06 | 2.92E-05 |
| TRBV10-2 | 0.574549 | 0.18198 | -1.65865 | 5.64E-06 | 2.94E-05 |
| IGKV1D-42 | 4.697697 | 1.36887 | -1.77897 | 5.70E-06 | 2.97E-05 |
| IGHV1-14 | 2.70751 | 1.242606 | -1.1236 | 5.73E-06 | 2.98E-05 |
| IGKV1D-17 | 4.973845 | 1.983991 | -1.32596 | 5.73E-06 | 2.98E-05 |
| IGKV2OR22-3 | 1.638146 | 0.585038 | -1.48546 | 5.74E-06 | 2.98E-05 |
| UNC5B-AS1 | 11.07157 | 4.483385 | -1.3042 | 5.75E-06 | 2.99E-05 |
| E2F6P4 | 0.417716 | 1.1866 | 1.506237 | 5.82E-06 | 3.02E-05 |
| IGHV3-33 | 119.3842 | 40.0413 | -1.57605 | 5.83E-06 | 3.02E-05 |
| IGHV4-61 | 26.98177 | 9.691024 | -1.47726 | 5.93E-06 | 3.07E-05 |
| ALOX12B | 37.47544 | 17.50913 | -1.09784 | 5.95E-06 | 3.08E-05 |
| AC128687.2 | 0.248371 | 0.109398 | -1.18291 | 6.04E-06 | 3.12E-05 |
| AC005393.1 | 1.391058 | 0.631164 | -1.14009 | 6.06E-06 | 3.13E-05 |
| SLC27A6 | 0.773862 | 0.306979 | -1.33393 | 6.10E-06 | 3.15E-05 |
| LINC02026 | 0.419081 | 0.163465 | -1.35825 | 6.12E-06 | 3.16E-05 |
| IGHV3-49 | 94.0091 | 46.21592 | -1.02441 | 6.15E-06 | 3.17E-05 |
| REN | 0.455139 | 0.172589 | -1.39897 | 6.19E-06 | 3.19E-05 |
| KCNA2 | 0.542247 | 0.141009 | -1.94316 | 6.20E-06 | 3.19E-05 |
| SPRR2E | 2036.604 | 811.0168 | -1.32836 | 6.23E-06 | 3.21E-05 |
| RHOXF1-AS1 | 3.213764 | 0.801516 | -2.00346 | 6.27E-06 | 3.22E-05 |
| LINC00092 | 0.590806 | 0.238202 | -1.3105 | 6.27E-06 | 3.22E-05 |
| AC244090.2 | 0.422744 | 0.199562 | -1.08295 | 6.38E-06 | 3.27E-05 |
| LY6G6C | 68.84833 | 31.51524 | -1.12737 | 6.38E-06 | 3.27E-05 |
| MMP3 | 151.0413 | 311.7082 | 1.045253 | 6.41E-06 | 3.28E-05 |
| BCL2L15 | 0.491617 | 0.223463 | -1.1375 | 6.43E-06 | 3.29E-05 |
| LYNX1 | 75.35906 | 32.79213 | -1.20043 | 6.54E-06 | 3.34E-05 |
| AC246787.2 | 0.376273 | 0.155105 | -1.27853 | 6.92E-06 | 3.51E-05 |
| MT-TT | 11.32741 | 5.562727 | -1.02595 | 7.00E-06 | 3.55E-05 |
| RNU1-72P | 0.281865 | 0.671546 | 1.25248 | 7.08E-06 | 3.58E-05 |
| TAGLN3 | 1.765866 | 4.378759 | 1.310146 | 7.27E-06 | 3.67E-05 |
| CLIC6 | 8.380509 | 3.485594 | -1.26563 | 7.28E-06 | 3.67E-05 |
| RHOXF1 | 0.639295 | 0.184526 | -1.79266 | 7.32E-06 | 3.69E-05 |
| TRAV20 | 0.838987 | 0.380197 | -1.1419 | 7.44E-06 | 3.74E-05 |
| BRME1 | 5.387922 | 2.538218 | -1.08591 | 7.58E-06 | 3.80E-05 |
| FOXA1 | 15.00508 | 4.708637 | -1.67207 | 7.87E-06 | 3.93E-05 |
| FOXJ1 | 4.420752 | 2.064118 | -1.09877 | 7.89E-06 | 3.94E-05 |
| POMC | 8.320872 | 2.55975 | -1.70073 | 8.00E-06 | 3.99E-05 |
| IGHV3-66 | 24.30162 | 10.47099 | -1.21466 | 8.05E-06 | 4.01E-05 |
| FAM240C | 2.121642 | 0.770835 | -1.46069 | 8.13E-06 | 4.05E-05 |
| IGHV1-67 | 6.521561 | 2.921013 | -1.15875 | 8.14E-06 | 4.05E-05 |
| AL663074.1 | 0.210411 | 0.422047 | 1.004192 | 8.16E-06 | 4.06E-05 |
| TRAV21 | 2.307436 | 1.152613 | -1.00138 | 8.21E-06 | 4.08E-05 |
| ASRGL1 | 2.721082 | 1.066204 | -1.3517 | 8.24E-06 | 4.09E-05 |
| ZDHHC11B | 3.402109 | 1.421176 | -1.25934 | 8.38E-06 | 4.15E-05 |
| AC069148.1 | 1.222579 | 0.514722 | -1.24806 | 8.53E-06 | 4.22E-05 |
| IGLV2-33 | 1.529122 | 0.4611 | -1.72955 | 8.54E-06 | 4.23E-05 |
| ST8SIA6 | 0.764702 | 0.365281 | -1.06589 | 8.55E-06 | 4.23E-05 |
| DAPL1 | 55.04702 | 22.94792 | -1.2623 | 8.73E-06 | 4.31E-05 |
| RNFT2 | 1.639548 | 0.781845 | -1.06834 | 8.76E-06 | 4.32E-05 |
| IGHV4-28 | 30.90491 | 10.61774 | -1.54136 | 8.83E-06 | 4.35E-05 |
| KRT77 | 1.63723 | 0.504943 | -1.69706 | 8.83E-06 | 4.35E-05 |
| AC084819.1 | 0.942207 | 0.228349 | -2.0448 | 8.94E-06 | 4.40E-05 |
| IGHV2-26 | 71.04823 | 23.7932 | -1.57825 | 9.17E-06 | 4.49E-05 |
| TRBV5-6 | 1.570845 | 0.593227 | -1.40489 | 9.17E-06 | 4.49E-05 |
| IRAG2 | 4.642187 | 1.874496 | -1.3083 | 9.30E-06 | 4.55E-05 |
| LINC01726 | 0.30972 | 0.024766 | -3.64456 | 9.34E-06 | 4.57E-05 |
| ELN | 20.34094 | 9.094743 | -1.16128 | 9.36E-06 | 4.57E-05 |
| AC010343.3 | 2.17301 | 4.773944 | 1.135487 | 9.37E-06 | 4.57E-05 |
| IGKV2D-30 | 1.787379 | 0.582982 | -1.61632 | 9.38E-06 | 4.58E-05 |
| CYP27A1 | 25.26535 | 9.133177 | -1.46797 | 9.52E-06 | 4.63E-05 |
| MYCN | 2.921556 | 0.861046 | -1.76258 | 9.58E-06 | 4.66E-05 |
| AC022182.2 | 0.383895 | 0.171682 | -1.16097 | 9.65E-06 | 4.68E-05 |
| MSMB | 106.6119 | 45.1722 | -1.23886 | 9.74E-06 | 4.72E-05 |
| ADCY6-DT | 0.272939 | 0.102907 | -1.40724 | 9.85E-06 | 4.77E-05 |
| IGLV4-69 | 169.213 | 55.14615 | -1.61751 | 9.94E-06 | 4.81E-05 |
| SPOCK2 | 22.39911 | 10.72837 | -1.06201 | 1.00E-05 | 4.85E-05 |
| IGHV3OR16-7 | 0.428658 | 0.149499 | -1.51969 | 1.00E-05 | 4.85E-05 |
| BEX2 | 24.7556 | 11.5256 | -1.10291 | 1.01E-05 | 4.87E-05 |
| GDA | 4.658286 | 2.069939 | -1.17021 | 1.01E-05 | 4.89E-05 |
| TMEM178A | 0.879777 | 0.31882 | -1.46439 | 1.02E-05 | 4.91E-05 |
| FCRL1 | 0.346382 | 0.112408 | -1.62362 | 1.02E-05 | 4.93E-05 |
| IGHV3-16 | 0.414403 | 0.189448 | -1.12924 | 1.06E-05 | 5.09E-05 |
| AC142381.1 | 4.52682 | 1.843605 | -1.29597 | 1.09E-05 | 5.20E-05 |
| HUNK | 1.969693 | 0.907606 | -1.11783 | 1.09E-05 | 5.23E-05 |
| AC005363.2 | 0.399252 | 0.189022 | -1.07875 | 1.10E-05 | 5.24E-05 |
| FMO2 | 10.42088 | 3.380174 | -1.62431 | 1.11E-05 | 5.29E-05 |
| LINC01767 | 0.455551 | 0.130291 | -1.80588 | 1.13E-05 | 5.37E-05 |
| TRBV18 | 1.712257 | 0.836965 | -1.03266 | 1.13E-05 | 5.38E-05 |
| AC131097.2 | 0.809816 | 0.322774 | -1.32707 | 1.15E-05 | 5.47E-05 |
| IGKV2-18 | 0.829848 | 0.315675 | -1.39441 | 1.15E-05 | 5.48E-05 |
| IGLV2-11 | 267.2384 | 102.7433 | -1.37908 | 1.16E-05 | 5.52E-05 |
| AC244034.2 | 0.589062 | 0.24172 | -1.28508 | 1.19E-05 | 5.63E-05 |
| KRT4 | 581.2022 | 69.7689 | -3.05838 | 1.19E-05 | 5.63E-05 |
| IGHV3OR16-9 | 5.991365 | 1.505398 | -1.99274 | 1.21E-05 | 5.69E-05 |
| CR2 | 1.440761 | 0.455504 | -1.66129 | 1.22E-05 | 5.76E-05 |
| IGHV1OR16-3 | 1.884204 | 0.746336 | -1.33606 | 1.24E-05 | 5.86E-05 |
| CSF3 | 10.86156 | 28.42006 | 1.387678 | 1.27E-05 | 5.97E-05 |
| IGKV3D-7 | 1.400416 | 0.347619 | -2.01028 | 1.29E-05 | 6.03E-05 |
| CYP4Z1 | 0.496909 | 0.14666 | -1.76051 | 1.31E-05 | 6.10E-05 |
| IGHV3OR16-15 | 1.284912 | 0.298544 | -2.10565 | 1.33E-05 | 6.20E-05 |
| IGHV4-4 | 18.15894 | 6.143996 | -1.56343 | 1.35E-05 | 6.26E-05 |
| LIM2 | 1.641705 | 0.664299 | -1.30529 | 1.36E-05 | 6.31E-05 |
| TRDC | 4.2537 | 1.962929 | -1.11571 | 1.36E-05 | 6.32E-05 |
| ESRG | 6.370344 | 1.168713 | -2.44645 | 1.37E-05 | 6.35E-05 |
| GYS2 | 0.251393 | 0.041721 | -2.59108 | 1.38E-05 | 6.38E-05 |
| CD8B2 | 10.15094 | 0.468706 | -4.43679 | 1.40E-05 | 6.46E-05 |
| AL133467.1 | 0.731947 | 0.357566 | -1.03353 | 1.40E-05 | 6.48E-05 |
| ALOX12 | 15.44224 | 6.810346 | -1.18108 | 1.41E-05 | 6.53E-05 |
| IGKV1OR22-5 | 1.814034 | 0.483804 | -1.90671 | 1.44E-05 | 6.65E-05 |
| C20orf197 | 1.463567 | 3.134304 | 1.098656 | 1.46E-05 | 6.73E-05 |
| DCC | 0.2493 | 0.078836 | -1.66096 | 1.49E-05 | 6.82E-05 |
| PTN | 47.77205 | 16.81381 | -1.50652 | 1.49E-05 | 6.85E-05 |
| IGKV1D-43 | 3.344841 | 1.16753 | -1.51848 | 1.52E-05 | 6.96E-05 |
| IGHV3-11 | 167.6072 | 71.60583 | -1.22693 | 1.53E-05 | 6.98E-05 |
| SPRR2D | 1158.592 | 575.6424 | -1.00913 | 1.62E-05 | 7.34E-05 |
| IGLV1-41 | 10.98684 | 4.744953 | -1.21131 | 1.62E-05 | 7.37E-05 |
| AC083841.1 | 3.07165 | 1.108742 | -1.47009 | 1.64E-05 | 7.44E-05 |
| IGHV3-73 | 92.1498 | 31.79757 | -1.53506 | 1.65E-05 | 7.47E-05 |
| LRRC37A14P | 0.245365 | 0.105597 | -1.21636 | 1.66E-05 | 7.53E-05 |
| IFNG-AS1 | 0.27147 | 0.120368 | -1.17334 | 1.68E-05 | 7.59E-05 |
| AC083801.2 | 0.98347 | 0.43397 | -1.18029 | 1.71E-05 | 7.73E-05 |
| HS3ST5 | 0.800125 | 0.359985 | -1.15229 | 1.74E-05 | 7.85E-05 |
| RAB39B | 0.806544 | 0.384397 | -1.06915 | 1.78E-05 | 7.99E-05 |
| AP003500.1 | 0.570693 | 0.226333 | -1.33427 | 1.78E-05 | 8.00E-05 |
| FGF14-AS2 | 2.149348 | 0.975984 | -1.13897 | 1.79E-05 | 8.01E-05 |
| VSIG8 | 15.42261 | 6.338192 | -1.2829 | 1.79E-05 | 8.04E-05 |
| IGLV8-61 | 89.38942 | 25.24057 | -1.82436 | 1.80E-05 | 8.06E-05 |
| FASLG | 2.540081 | 1.259427 | -1.01211 | 1.85E-05 | 8.26E-05 |
| ALKAL2 | 0.918794 | 0.420248 | -1.1285 | 1.85E-05 | 8.26E-05 |
| IGLV2-34 | 5.424801 | 1.289537 | -2.07272 | 1.85E-05 | 8.28E-05 |
| DPT | 28.60963 | 14.24012 | -1.00654 | 1.87E-05 | 8.35E-05 |
| CSF2RBP1 | 0.380432 | 0.114402 | -1.73353 | 1.88E-05 | 8.38E-05 |
| KRT36 | 4.926178 | 0.222475 | -4.46875 | 1.88E-05 | 8.38E-05 |
| NELL1 | 0.802228 | 0.30576 | -1.39161 | 1.92E-05 | 8.52E-05 |
| TSHR | 0.272325 | 0.091939 | -1.56658 | 1.93E-05 | 8.57E-05 |
| IGHJ3P | 12.74498 | 5.540095 | -1.20195 | 1.93E-05 | 8.58E-05 |
| LGI3 | 6.736356 | 1.971377 | -1.77276 | 1.96E-05 | 8.68E-05 |
| IGHJ2 | 48.23672 | 16.33636 | -1.56205 | 1.96E-05 | 8.69E-05 |
| IGLV5-45 | 33.63758 | 15.12042 | -1.15358 | 2.00E-05 | 8.83E-05 |
| BDNF | 1.221611 | 0.593622 | -1.04117 | 2.01E-05 | 8.89E-05 |
| STAG3 | 4.556453 | 1.291971 | -1.81834 | 2.07E-05 | 9.11E-05 |
| LINC02621 | 0.853293 | 0.384481 | -1.15013 | 2.09E-05 | 9.18E-05 |
| SYTL5 | 2.570237 | 1.060264 | -1.27748 | 2.10E-05 | 9.23E-05 |
| PYDC1 | 3.187866 | 0.466994 | -2.77112 | 2.11E-05 | 9.28E-05 |
| IGKV1OR2-6 | 6.7664 | 2.129598 | -1.66781 | 2.12E-05 | 9.29E-05 |
| AKR1B10 | 481.2322 | 225.3851 | -1.09434 | 2.16E-05 | 9.44E-05 |
| CDRT15P6 | 0.328768 | 0.13096 | -1.32795 | 2.20E-05 | 9.58E-05 |
| ZNF831 | 0.644758 | 0.303749 | -1.08588 | 2.26E-05 | 9.81E-05 |
| TMPRSS11F | 7.423613 | 3.415772 | -1.11991 | 2.27E-05 | 9.87E-05 |
| KCNH1 | 0.418456 | 0.15492 | -1.43355 | 2.29E-05 | 9.96E-05 |
| DBX2 | 0.462123 | 0.038964 | -3.56807 | 2.54E-05 | 0.000109 |
| CHL1-AS2 | 0.417142 | 0.180674 | -1.20715 | 2.68E-05 | 0.000115 |
| PCP4 | 4.279482 | 0.062874 | -6.08882 | 2.69E-05 | 0.000115 |
| IGKV1D-33 | 2.107633 | 0.677347 | -1.63766 | 2.72E-05 | 0.000116 |
| TMEM178B | 3.750578 | 0.681795 | -2.4597 | 2.85E-05 | 0.000121 |
| IGLV3-25 | 387.4176 | 162.2692 | -1.2555 | 2.88E-05 | 0.000122 |
| SPEF2 | 0.461904 | 0.21579 | -1.09797 | 2.91E-05 | 0.000123 |
| IGKJ5 | 15.80817 | 7.017756 | -1.17159 | 2.95E-05 | 0.000124 |
| DDC | 0.89601 | 0.162746 | -2.46089 | 2.99E-05 | 0.000126 |
| IGHV3OR16-17 | 4.345015 | 1.347775 | -1.68878 | 3.04E-05 | 0.000128 |
| IGHV3-48 | 53.09938 | 20.06371 | -1.40411 | 3.12E-05 | 0.000131 |
| CD177 | 42.83556 | 8.605463 | -2.31548 | 3.16E-05 | 0.000132 |
| AC004923.1 | 0.3618 | 0.127698 | -1.50245 | 3.22E-05 | 0.000135 |
| FDCSP | 1215.615 | 133.0306 | -3.19186 | 3.23E-05 | 0.000135 |
| AJAP1 | 2.187682 | 5.170503 | 1.240901 | 3.24E-05 | 0.000135 |
| AC004817.3 | 0.364893 | 1.231854 | 1.755284 | 3.26E-05 | 0.000136 |
| MMP2-AS1 | 0.523557 | 0.220614 | -1.24682 | 3.31E-05 | 0.000138 |
| NDUFB4P11 | 0.487493 | 0.172819 | -1.49612 | 3.32E-05 | 0.000138 |
| ZDHHC2 | 8.887797 | 4.179401 | -1.08853 | 3.33E-05 | 0.000139 |
| FGL1 | 0.170388 | 0.446901 | 1.391135 | 3.44E-05 | 0.000143 |
| STAP1 | 1.645622 | 0.796146 | -1.04753 | 3.55E-05 | 0.000147 |
| AC103563.3 | 3.748059 | 1.493873 | -1.32709 | 3.56E-05 | 0.000147 |
| IGHV3OR16-11 | 1.113083 | 0.341276 | -1.70555 | 3.56E-05 | 0.000147 |
| CSMD2 | 0.534393 | 1.105988 | 1.049363 | 3.57E-05 | 0.000147 |
| APOC1 | 78.72814 | 38.07837 | -1.04791 | 3.61E-05 | 0.000149 |
| NEFH | 9.275017 | 0.520643 | -4.15498 | 3.67E-05 | 0.000151 |
| SPRR2B | 260.8906 | 79.04242 | -1.72275 | 3.68E-05 | 0.000152 |
| ST8SIA6-AS1 | 0.531395 | 0.237453 | -1.16214 | 3.70E-05 | 0.000152 |
| MIR593 | 0.352079 | 0.815268 | 1.211377 | 3.77E-05 | 0.000155 |
| CYP4F22 | 19.74224 | 9.134856 | -1.11183 | 3.83E-05 | 0.000157 |
| INA | 3.675542 | 0.848834 | -2.1144 | 3.86E-05 | 0.000158 |
| GFRA3 | 3.312512 | 1.615749 | -1.03572 | 3.92E-05 | 0.00016 |
| TMEM150C | 7.841319 | 3.01732 | -1.37783 | 3.94E-05 | 0.000161 |
| TOB1-AS1 | 2.837366 | 0.727722 | -1.96309 | 3.98E-05 | 0.000162 |
| SYP | 1.142174 | 0.478124 | -1.25633 | 3.99E-05 | 0.000163 |
| IGHV4-34 | 146.2418 | 67.06425 | -1.12474 | 4.02E-05 | 0.000164 |
| OR7E22P | 0.883355 | 0.395361 | -1.15982 | 4.03E-05 | 0.000164 |
| IGLV3-21 | 600.6071 | 223.1303 | -1.42854 | 4.08E-05 | 0.000166 |
| SVOPL | 0.308682 | 0.10973 | -1.49216 | 4.15E-05 | 0.000168 |
| PRR15L | 7.614316 | 3.614934 | -1.07475 | 4.16E-05 | 0.000169 |
| DNASE2B | 0.391033 | 0.134627 | -1.53833 | 4.16E-05 | 0.000169 |
| IGFL4 | 0.966498 | 0.327145 | -1.56284 | 4.24E-05 | 0.000172 |
| AC120036.3 | 1.06096 | 0.517852 | -1.03476 | 4.25E-05 | 0.000172 |
| AC073534.2 | 0.294244 | 0.132421 | -1.15188 | 4.27E-05 | 0.000173 |
| UBD | 40.71585 | 15.0239 | -1.43833 | 4.33E-05 | 0.000175 |
| IGHV1-17 | 1.489381 | 0.598096 | -1.31626 | 4.34E-05 | 0.000175 |
| IGHV3-64 | 23.92401 | 6.315005 | -1.9216 | 4.38E-05 | 0.000177 |
| LINC02448 | 0.472025 | 0.196042 | -1.2677 | 4.51E-05 | 0.000182 |
| IGHV1-69D | 221.3425 | 92.34447 | -1.26118 | 4.53E-05 | 0.000182 |
| IGLV4-60 | 32.0125 | 8.983631 | -1.83326 | 4.56E-05 | 0.000183 |
| VNN1 | 6.322006 | 3.118735 | -1.01942 | 4.60E-05 | 0.000185 |
| SERPINI2 | 0.258883 | 0.044093 | -2.55369 | 4.72E-05 | 0.000189 |
| AC011473.3 | 0.609764 | 0.241061 | -1.33885 | 4.85E-05 | 0.000193 |
| IGHV2-5 | 29.6633 | 11.47672 | -1.36997 | 4.88E-05 | 0.000194 |
| CACNA1D | 0.316953 | 0.132942 | -1.25348 | 4.91E-05 | 0.000195 |
| PPARG | 2.749141 | 1.300425 | -1.08 | 4.97E-05 | 0.000197 |
| SLURP1 | 219.5522 | 69.3139 | -1.66335 | 4.98E-05 | 0.000197 |
| IGKV1OR9-2 | 0.702448 | 0.257814 | -1.44606 | 5.30E-05 | 0.000208 |
| AL359979.2 | 0.410402 | 0.081962 | -2.32401 | 5.40E-05 | 0.000212 |
| AC005920.4 | 0.530143 | 0.257371 | -1.04253 | 5.46E-05 | 0.000214 |
| GPR62 | 0.293363 | 0.132058 | -1.15151 | 5.65E-05 | 0.000221 |
| IGLV1-36 | 48.9194 | 17.58288 | -1.47624 | 5.73E-05 | 0.000223 |
| IGHV3OR16-10 | 1.670641 | 0.664436 | -1.3302 | 5.75E-05 | 0.000224 |
| AP002498.1 | 0.448851 | 0.208924 | -1.10326 | 5.92E-05 | 0.00023 |
| DUSP15 | 0.574288 | 0.273017 | -1.07278 | 6.01E-05 | 0.000233 |
| CALML6 | 0.581053 | 0.266563 | -1.12419 | 6.05E-05 | 0.000235 |
| AL138789.1 | 5.865068 | 13.53889 | 1.20689 | 6.08E-05 | 0.000236 |
| IGKV1D-39 | 6.936123 | 1.5804 | -2.13384 | 6.16E-05 | 0.000238 |
| IGKV7-3 | 1.431119 | 0.613258 | -1.22258 | 6.17E-05 | 0.000239 |
| LINC01549 | 0.276775 | 0.107585 | -1.36324 | 6.21E-05 | 0.00024 |
| ABCA17P | 1.000764 | 0.157389 | -2.6687 | 6.32E-05 | 0.000244 |
| SPINK7 | 53.32686 | 9.448452 | -2.49671 | 6.41E-05 | 0.000247 |
| TRBJ2-2 | 1.413332 | 0.622102 | -1.18388 | 6.45E-05 | 0.000248 |
| CLDN3 | 19.53792 | 1.980386 | -3.30242 | 6.48E-05 | 0.000249 |
| PLAAT5 | 0.783897 | 0.263178 | -1.57463 | 6.53E-05 | 0.000251 |
| CRISP3 | 12.29408 | 5.484262 | -1.16459 | 6.55E-05 | 0.000251 |
| MRAP2 | 11.26574 | 4.247816 | -1.40715 | 6.70E-05 | 0.000256 |
| RHBDL3 | 2.129353 | 0.692049 | -1.62147 | 6.70E-05 | 0.000256 |
| SULT1B1 | 1.099785 | 0.44854 | -1.29391 | 6.76E-05 | 0.000258 |
| IGLV3-27 | 58.80999 | 17.6533 | -1.73612 | 6.79E-05 | 0.000259 |
| IGHV3-36 | 0.366909 | 0.128327 | -1.51559 | 7.06E-05 | 0.000268 |
| MAOB | 17.44349 | 8.513326 | -1.03489 | 7.33E-05 | 0.000277 |
| AC078909.2 | 0.344454 | 0.161349 | -1.09413 | 7.43E-05 | 0.000281 |
| PACRG | 0.367005 | 0.169639 | -1.11333 | 7.76E-05 | 0.000291 |
| AF131216.3 | 1.223431 | 0.438319 | -1.48088 | 7.77E-05 | 0.000292 |
| SLC9A2 | 3.949016 | 0.866689 | -2.18791 | 7.79E-05 | 0.000292 |
| SCUBE2 | 3.199954 | 1.38055 | -1.21281 | 7.83E-05 | 0.000294 |
| CHST9 | 0.939759 | 0.331283 | -1.50423 | 8.04E-05 | 0.0003 |
| AC034223.2 | 2.108986 | 4.74935 | 1.171181 | 8.09E-05 | 0.000302 |
| FAM171A1 | 13.4259 | 6.035516 | -1.15347 | 8.10E-05 | 0.000302 |
| PRELP | 13.72447 | 6.484569 | -1.08167 | 8.15E-05 | 0.000304 |
| COLGALT2 | 2.092782 | 0.783547 | -1.41733 | 8.15E-05 | 0.000304 |
| ORM2 | 0.780511 | 0.25341 | -1.62294 | 8.26E-05 | 0.000308 |
| IGHV3-20 | 16.54238 | 8.169184 | -1.0179 | 8.31E-05 | 0.00031 |
| TDH | 0.70098 | 0.326088 | -1.10411 | 8.49E-05 | 0.000316 |
| DCT | 0.344968 | 0.030366 | -3.50593 | 8.72E-05 | 0.000322 |
| RSPH14 | 0.56304 | 0.281317 | -1.00104 | 8.77E-05 | 0.000324 |
| TRBV23-1 | 0.360438 | 0.152487 | -1.24107 | 8.78E-05 | 0.000324 |
| LINC02091 | 0.533676 | 0.260528 | -1.03453 | 8.90E-05 | 0.000328 |
| AP001099.1 | 0.813707 | 0.166253 | -2.29113 | 8.96E-05 | 0.00033 |
| COL26A1 | 2.122107 | 0.464264 | -2.19248 | 8.99E-05 | 0.000331 |
| CNR1 | 0.4351 | 0.186207 | -1.22444 | 9.00E-05 | 0.000331 |
| CES3 | 2.108945 | 0.870898 | -1.27595 | 9.07E-05 | 0.000333 |
| IGLV9-49 | 35.16314 | 15.89052 | -1.1459 | 9.09E-05 | 0.000334 |
| SPRR2C | 179.0055 | 75.49225 | -1.2456 | 9.20E-05 | 0.000338 |
| AMH | 2.189167 | 1.049839 | -1.06021 | 9.31E-05 | 0.000341 |
| LINC01474 | 0.233414 | 0.105143 | -1.15054 | 9.33E-05 | 0.000342 |
| RPSAP69 | 0.7038 | 0.295678 | -1.25114 | 9.37E-05 | 0.000343 |
| TRAV38-1 | 0.473798 | 0.181929 | -1.38089 | 9.38E-05 | 0.000343 |
| IGHV5-51 | 479.4756 | 188.8332 | -1.34435 | 9.75E-05 | 0.000356 |
| KLK12 | 58.93707 | 25.98086 | -1.18173 | 9.99E-05 | 0.000364 |
| TRBV4-2 | 2.074499 | 0.839057 | -1.30592 | 0.000101 | 0.000367 |
| SYNE4 | 2.800141 | 1.007007 | -1.47543 | 0.000101 | 0.000367 |
| LCE1E | 0.480475 | 0.148318 | -1.69577 | 0.000102 | 0.00037 |
| SCGB2A1 | 6.753298 | 0.368417 | -4.19618 | 0.000102 | 0.000371 |
| MGAT3-AS1 | 0.558167 | 0.088622 | -2.65497 | 0.000102 | 0.000371 |
| RPE65 | 3.702392 | 8.643312 | 1.223127 | 0.000103 | 0.000372 |
| GCNT2 | 3.120655 | 1.372859 | -1.18467 | 0.000108 | 0.000392 |
| IGLV3-29 | 2.313629 | 0.649844 | -1.83199 | 0.00011 | 0.000397 |
| TRBJ2-2P | 2.250916 | 0.979347 | -1.20062 | 0.000111 | 0.0004 |
| RNU6-167P | 1.191842 | 0.543883 | -1.13182 | 0.000111 | 0.0004 |
| AC087392.2 | 0.377371 | 0.171832 | -1.13498 | 0.000112 | 0.000402 |
| IGLV4-3 | 2.377586 | 0.33653 | -2.82069 | 0.000112 | 0.000404 |
| DKK4 | 1.419885 | 0.435796 | -1.70405 | 0.000113 | 0.000406 |
| SLCO4C1 | 1.110911 | 0.392242 | -1.50193 | 0.000113 | 0.000407 |
| TRBV10-3 | 1.461209 | 0.715499 | -1.03014 | 0.000114 | 0.000409 |
| B3GALT5-AS1 | 1.1997 | 0.203427 | -2.56009 | 0.000116 | 0.000416 |
| CLCA4 | 33.32664 | 14.69011 | -1.18183 | 0.000116 | 0.000416 |
| IGHV3-21 | 194.9308 | 88.43922 | -1.1402 | 0.000117 | 0.000418 |
| LTF | 131.8263 | 56.84952 | -1.21342 | 0.000121 | 0.000431 |
| CLDN6 | 1.295897 | 3.794992 | 1.550146 | 0.000122 | 0.000434 |
| RNF128 | 10.96487 | 22.85442 | 1.059585 | 0.000122 | 0.000435 |
| TGM6 | 0.841417 | 0.20559 | -2.03305 | 0.000125 | 0.000443 |
| IL17RB | 4.344554 | 1.825004 | -1.25131 | 0.000125 | 0.000443 |
| ADARB2 | 0.945207 | 0.223643 | -2.07943 | 0.000127 | 0.000449 |
| KCNH8 | 0.439509 | 0.114749 | -1.93742 | 0.000128 | 0.000451 |
| LINC01697 | 0.31325 | 0.124515 | -1.33099 | 0.000129 | 0.000454 |
| CCKAR | 0.79677 | 0.233703 | -1.76949 | 0.000129 | 0.000455 |
| AL109914.1 | 0.648739 | 0.27678 | -1.2289 | 0.000134 | 0.00047 |
| TRBV27 | 0.996443 | 0.450771 | -1.14439 | 0.000135 | 0.000473 |
| AC010307.4 | 0.484413 | 0.175717 | -1.46298 | 0.000138 | 0.000484 |
| AC015722.2 | 1.098975 | 0.141578 | -2.95649 | 0.00014 | 0.000488 |
| SBSPON | 3.357365 | 1.306021 | -1.36215 | 0.00014 | 0.000488 |
| CNTD1 | 1.095189 | 0.517245 | -1.08226 | 0.00014 | 0.00049 |
| ITLN1 | 0.935741 | 0.152325 | -2.61896 | 0.000141 | 0.000493 |
| TREX2 | 6.53558 | 3.044708 | -1.10201 | 0.000143 | 0.000497 |
| OLFM1 | 20.23973 | 9.43431 | -1.1012 | 0.000143 | 0.000498 |
| IGKV1-13 | 3.244052 | 0.874747 | -1.89086 | 0.000145 | 0.000506 |
| LINC01537 | 0.451515 | 1.622267 | 1.845166 | 0.000148 | 0.000512 |
| IGHV4-39 | 358.5738 | 157.5683 | -1.18629 | 0.000148 | 0.000515 |
| NPM2 | 3.733363 | 1.84427 | -1.01743 | 0.000149 | 0.000517 |
| AC006262.2 | 1.458484 | 3.140413 | 1.106484 | 0.000151 | 0.000521 |
| ZFR2 | 1.466818 | 0.16685 | -3.13606 | 0.000154 | 0.00053 |
| DLGAP1-AS5 | 0.723248 | 0.074709 | -3.27513 | 0.000155 | 0.000534 |
| IGKV1D-12 | 7.117325 | 1.227726 | -2.53535 | 0.000158 | 0.000543 |
| CD22 | 1.906905 | 0.887142 | -1.104 | 0.000159 | 0.000547 |
| PCDH19 | 3.102549 | 1.195578 | -1.37575 | 0.00016 | 0.00055 |
| AL596223.1 | 0.331308 | 0.858013 | 1.372826 | 0.000162 | 0.000555 |
| TRBV30 | 1.547671 | 0.660661 | -1.22812 | 0.000162 | 0.000556 |
| IL19 | 1.923402 | 0.55611 | -1.79022 | 0.000164 | 0.00056 |
| LCE1B | 2.281847 | 0.554308 | -2.04144 | 0.000166 | 0.000568 |
| LINC01857 | 1.514166 | 0.70015 | -1.11279 | 0.000167 | 0.000568 |
| LDLRAD1 | 0.745915 | 0.305003 | -1.29019 | 0.00017 | 0.000577 |
| B3GALT2 | 0.4857 | 0.205764 | -1.23907 | 0.000175 | 0.000594 |
| ITLN2 | 0.597763 | 0.175055 | -1.77176 | 0.000178 | 0.000605 |
| IGHV1-45 | 8.068071 | 2.803294 | -1.5251 | 0.000181 | 0.000614 |
| EPHA7 | 2.778403 | 0.40122 | -2.79179 | 0.000182 | 0.000616 |
| VNN3 | 2.055853 | 0.983583 | -1.06362 | 0.000184 | 0.000621 |
| ADH1C | 14.79313 | 1.676495 | -3.14141 | 0.000184 | 0.000623 |
| VEGFD | 0.648511 | 0.319764 | -1.02012 | 0.000188 | 0.000635 |
| AC016229.2 | 0.355794 | 0.150119 | -1.24493 | 0.000189 | 0.000639 |
| MCHR1 | 0.46921 | 1.068302 | 1.187014 | 0.00019 | 0.00064 |
| IGHV4-31 | 94.53565 | 37.96342 | -1.31625 | 0.000191 | 0.000645 |
| FCRL3 | 0.920305 | 0.453172 | -1.02205 | 0.000193 | 0.00065 |
| FEZF1-AS1 | 1.957979 | 0.86278 | -1.1823 | 0.000198 | 0.000663 |
| IGLVI-70 | 5.125557 | 1.292996 | -1.98699 | 0.000204 | 0.000681 |
| PLAAT3 | 13.62075 | 6.355898 | -1.09964 | 0.000206 | 0.000688 |
| SCN4B | 4.148617 | 9.539522 | 1.201287 | 0.00021 | 0.000698 |
| PIWIL1 | 0.163896 | 0.389845 | 1.25012 | 0.000213 | 0.000706 |
| KCNJ18 | 2.02667 | 0.865394 | -1.22768 | 0.000215 | 0.000712 |
| EPHA10 | 0.599161 | 0.28394 | -1.07736 | 0.000216 | 0.000716 |
| RIC3 | 0.584364 | 0.231285 | -1.3372 | 0.000218 | 0.000722 |
| FALEC | 1.252901 | 0.604499 | -1.05146 | 0.000219 | 0.000723 |
| IGHV2-70 | 61.0531 | 27.48554 | -1.15139 | 0.00022 | 0.000727 |
| LCE1C | 10.05747 | 3.782846 | -1.41072 | 0.000236 | 0.00077 |
| LRRC26 | 0.535226 | 0.26062 | -1.0382 | 0.000237 | 0.000774 |
| AC004490.1 | 0.234843 | 0.09573 | -1.29465 | 0.000238 | 0.000778 |
| NLGN1 | 0.264706 | 0.09569 | -1.46795 | 0.000239 | 0.000778 |
| AC007240.1 | 0.867483 | 0.383085 | -1.17917 | 0.00024 | 0.000781 |
| SBSN | 1182.567 | 557.4701 | -1.08496 | 0.000241 | 0.000785 |
| LONRF2 | 0.482316 | 0.182569 | -1.40154 | 0.00025 | 0.000809 |
| AMOT | 4.828538 | 2.233305 | -1.11241 | 0.000251 | 0.000812 |
| IGKV1OR2-108 | 36.46174 | 10.30213 | -1.82344 | 0.000251 | 0.000814 |
| TRBJ2-3 | 3.064755 | 1.499492 | -1.0313 | 0.000252 | 0.000815 |
| GABRG3 | 0.263792 | 0.10131 | -1.38063 | 0.000259 | 0.000835 |
| CSMD1 | 0.427223 | 0.167218 | -1.35326 | 0.00027 | 0.000866 |
| LINC01886 | 0.288728 | 0.129242 | -1.15964 | 0.000277 | 0.000884 |
| F7 | 0.309172 | 0.135148 | -1.19387 | 0.000284 | 0.000904 |
| CLDN8 | 5.264409 | 2.3941 | -1.13679 | 0.000286 | 0.00091 |
| OSTN-AS1 | 0.287777 | 0.080477 | -1.83831 | 0.000289 | 0.000919 |
| KRT42P | 6.461053 | 3.05812 | -1.07912 | 0.00029 | 0.000921 |
| TRAV10 | 0.650355 | 0.293934 | -1.14573 | 0.000294 | 0.000932 |
| TDRD10 | 1.838794 | 0.368849 | -2.31766 | 0.000296 | 0.000938 |
| AL513485.1 | 0.355268 | 0.142064 | -1.32236 | 0.000298 | 0.000944 |
| CDH4 | 0.932838 | 2.13426 | 1.194038 | 0.000301 | 0.000952 |
| C6orf15 | 36.31708 | 13.87409 | -1.38825 | 0.000308 | 0.000971 |
| SOX2-OT | 1.271525 | 0.344942 | -1.88213 | 0.00031 | 0.000977 |
| MT-TM | 1.444756 | 0.574314 | -1.33091 | 0.000311 | 0.000978 |
| IGHV1OR21-1 | 3.376205 | 1.452844 | -1.21652 | 0.000315 | 0.000991 |
| FXYD6 | 6.802545 | 3.156826 | -1.1076 | 0.000317 | 0.000996 |
| GPR15 | 3.687834 | 1.428977 | -1.36779 | 0.000322 | 0.001009 |
| CEL | 23.43929 | 10.21913 | -1.19766 | 0.000332 | 0.001039 |
| LINC01914 | 0.915427 | 0.3916 | -1.22506 | 0.000342 | 0.001064 |
| C1QTNF4 | 0.786764 | 0.158284 | -2.31342 | 0.000353 | 0.001094 |
| AC015961.2 | 0.504102 | 0.24229 | -1.05698 | 0.00037 | 0.00114 |
| AC093904.3 | 1.930866 | 0.608641 | -1.66558 | 0.000372 | 0.001146 |
| FOXI1 | 0.471173 | 0.177063 | -1.41199 | 0.000373 | 0.001149 |
| PDE2A-AS2 | 1.850586 | 3.784573 | 1.032149 | 0.000374 | 0.001152 |
| IGHV3-43 | 47.81462 | 14.77541 | -1.69425 | 0.000387 | 0.001188 |
| AC243829.1 | 0.305682 | 0.137342 | -1.15426 | 0.000394 | 0.001206 |
| ALDH1L1 | 2.208046 | 0.711384 | -1.63407 | 0.000402 | 0.001226 |
| IGHV1-68 | 0.998304 | 0.235278 | -2.08511 | 0.000404 | 0.001234 |
| IGKV1OR2-118 | 0.439971 | 0.193185 | -1.18742 | 0.000413 | 0.001256 |
| AL138962.1 | 0.359172 | 0.755798 | 1.073328 | 0.000431 | 0.001304 |
| IL26 | 0.283986 | 0.137082 | -1.05078 | 0.000436 | 0.001317 |
| TEX15 | 0.394931 | 0.192466 | -1.03699 | 0.00044 | 0.001328 |
| P2RX2 | 0.262477 | 0.094723 | -1.4704 | 0.000441 | 0.001332 |
| AC135068.1 | 4.130366 | 1.473515 | -1.48701 | 0.000444 | 0.00134 |
| AC007319.1 | 1.422289 | 0.510455 | -1.47836 | 0.00045 | 0.001354 |
| IGSF10 | 1.209666 | 0.548301 | -1.14157 | 0.000455 | 0.001367 |
| FMO6P | 0.800428 | 0.232109 | -1.78597 | 0.000461 | 0.001384 |
| AC020659.1 | 0.525797 | 0.26054 | -1.013 | 0.000474 | 0.001417 |
| IGHV7-34-1 | 0.570458 | 0.27791 | -1.0375 | 0.000481 | 0.001436 |
| CDRT15P12 | 0.561288 | 0.270516 | -1.05302 | 0.000491 | 0.001464 |
| FSIP2-AS2 | 0.326975 | 0.154879 | -1.07804 | 0.000496 | 0.001476 |
| LINC01983 | 0.430129 | 0.126134 | -1.76981 | 0.000506 | 0.001503 |
| SMIM43 | 0.314303 | 0.154991 | -1.01997 | 0.000539 | 0.001592 |
| HMSD | 1.841625 | 0.693306 | -1.40942 | 0.000543 | 0.001603 |
| IGHV3-76 | 2.087596 | 0.873109 | -1.25761 | 0.000545 | 0.001607 |
| KRT15 | 386.3233 | 188.0347 | -1.03881 | 0.000551 | 0.001624 |
| ERICH5 | 1.455447 | 0.685701 | -1.08581 | 0.000562 | 0.001654 |
| AL390755.2 | 1.897603 | 0.227352 | -3.06118 | 0.000575 | 0.001685 |
| AL035701.1 | 0.304645 | 0.07839 | -1.95839 | 0.00058 | 0.001698 |
| AC010643.1 | 0.901524 | 0.37521 | -1.26467 | 0.000598 | 0.001745 |
| DUX4L27 | 0.283855 | 0.103264 | -1.45881 | 0.000599 | 0.001749 |
| DEFB4A | 109.6163 | 33.41475 | -1.71391 | 0.000607 | 0.001769 |
| AC037487.3 | 1.454384 | 0.68455 | -1.08718 | 0.000613 | 0.001782 |
| HMGN2P15 | 3.558911 | 1.434967 | -1.31042 | 0.000616 | 0.001791 |
| SLC22A1 | 1.95704 | 7.24312 | 1.887938 | 0.000621 | 0.001805 |
| LINC02598 | 0.490984 | 0.236342 | -1.0548 | 0.000622 | 0.001808 |
| AC009264.1 | 0.420914 | 0.192064 | -1.13194 | 0.000629 | 0.001825 |
| AL357143.1 | 1.226004 | 0.306074 | -2.00201 | 0.00063 | 0.001828 |
| AC083829.1 | 0.729 | 1.83197 | 1.329405 | 0.000669 | 0.001924 |
| RNF150 | 2.947793 | 1.041903 | -1.50041 | 0.000672 | 0.001931 |
| TRBV24-1 | 0.937857 | 0.43573 | -1.10593 | 0.000682 | 0.001953 |
| HTRA4 | 0.709954 | 0.33258 | -1.09402 | 0.000695 | 0.00199 |
| MAEL | 0.781631 | 0.048671 | -4.00536 | 0.000712 | 0.002032 |
| RORB | 1.614224 | 0.490608 | -1.7182 | 0.000723 | 0.00206 |
| LINC02700 | 0.810142 | 1.711565 | 1.079069 | 0.000733 | 0.002084 |
| SIM2 | 5.764419 | 1.518164 | -1.92485 | 0.000739 | 0.002099 |
| ANKRD30BP3 | 0.321293 | 0.109624 | -1.55133 | 0.00074 | 0.002103 |
| KRT2 | 17.02384 | 3.585258 | -2.24741 | 0.000742 | 0.002108 |
| TRBV7-4 | 0.4707 | 0.185825 | -1.34087 | 0.000742 | 0.002109 |
| SMCO1 | 0.400562 | 0.139235 | -1.52451 | 0.000743 | 0.002111 |
| CFAP300 | 1.68184 | 4.386484 | 1.383025 | 0.000744 | 0.002112 |
| IGHD | 45.50009 | 18.61192 | -1.28964 | 0.00075 | 0.002126 |
| AADAC | 5.887199 | 1.676519 | -1.81211 | 0.000751 | 0.002129 |
| TCERG1L | 0.580612 | 0.275453 | -1.07577 | 0.000767 | 0.002167 |
| GS1-24F4.2 | 0.236967 | 0.11317 | -1.06619 | 0.000788 | 0.002219 |
| PIP5K1B | 1.665525 | 0.791457 | -1.07339 | 0.000791 | 0.002227 |
| AL161668.4 | 0.278879 | 0.132242 | -1.07646 | 0.000798 | 0.002242 |
| HMX2 | 0.902216 | 0.2292 | -1.97687 | 0.000804 | 0.002258 |
| IGKV1OR10-1 | 0.912371 | 0.374299 | -1.28543 | 0.000805 | 0.002261 |
| LINC01337 | 0.26647 | 0.132792 | -1.00481 | 0.000827 | 0.002316 |
| LINC01033 | 0.481876 | 0.205572 | -1.22902 | 0.000835 | 0.002335 |
| HAL | 3.588759 | 0.934228 | -1.94164 | 0.00087 | 0.002421 |
| AC008759.2 | 0.429249 | 0.186746 | -1.20074 | 0.000883 | 0.002452 |
| AL512324.1 | 0.995406 | 0.407994 | -1.28674 | 0.000885 | 0.002458 |
| U8 | 0.264025 | 0.090115 | -1.55084 | 0.00092 | 0.002544 |
| DMRT1 | 0.521099 | 0.170848 | -1.60884 | 0.000921 | 0.002547 |
| REM1 | 2.606606 | 1.202251 | -1.11643 | 0.000944 | 0.002603 |
| CHST4 | 0.631975 | 0.234126 | -1.43258 | 0.000947 | 0.00261 |
| TMEM200C | 0.430491 | 0.146939 | -1.55077 | 0.000954 | 0.002625 |
| PRMT8 | 0.227277 | 0.692381 | 1.607118 | 0.000983 | 0.002695 |
| INSM1 | 3.095911 | 0.140956 | -4.45705 | 0.000991 | 0.002712 |
| OR2I1P | 34.40788 | 15.2627 | -1.17273 | 0.001025 | 0.002792 |
| NTSR1 | 0.80331 | 3.345686 | 2.058274 | 0.001029 | 0.002804 |
| TRAV24 | 0.829591 | 0.399859 | -1.05291 | 0.001048 | 0.002849 |
| UPK1B | 116.9612 | 43.93688 | -1.41253 | 0.001056 | 0.002867 |
| ADORA2A-AS1 | 0.44217 | 0.147615 | -1.58276 | 0.001094 | 0.002956 |
| AC011483.2 | 0.260884 | 0.096949 | -1.42811 | 0.001099 | 0.002968 |
| IGFALS | 0.381 | 0.163487 | -1.22061 | 0.0011 | 0.00297 |
| CYP4F23P | 0.258505 | 0.107474 | -1.26621 | 0.001101 | 0.002973 |
| AL022724.2 | 0.693432 | 0.193367 | -1.84241 | 0.001153 | 0.003088 |
| FCER2 | 0.894592 | 0.380686 | -1.23263 | 0.001154 | 0.003089 |
| ADAMTS18 | 0.338512 | 0.144403 | -1.2291 | 0.001174 | 0.003137 |
| EPYC | 3.175644 | 0.489769 | -2.69687 | 0.001251 | 0.003319 |
| DYDC2 | 0.338496 | 0.138804 | -1.28609 | 0.001272 | 0.003368 |
| KRT33B | 0.678566 | 0.325841 | -1.05832 | 0.001307 | 0.00345 |
| AL512324.3 | 0.302576 | 0.108234 | -1.48315 | 0.001318 | 0.003473 |
| ADAM22 | 0.888708 | 0.363932 | -1.28804 | 0.001345 | 0.003534 |
| CLDN10 | 5.912375 | 1.315848 | -2.16774 | 0.001352 | 0.003552 |
| AC026369.3 | 0.485064 | 0.195467 | -1.31125 | 0.001416 | 0.003699 |
| AC004808.2 | 0.333597 | 0.124342 | -1.42379 | 0.001433 | 0.003739 |
| NPW | 8.386368 | 3.997872 | -1.06881 | 0.001435 | 0.00374 |
| IGKV2-4 | 0.89974 | 0.3285 | -1.45361 | 0.001442 | 0.003758 |
| IL17A | 0.239233 | 0.106468 | -1.168 | 0.001459 | 0.0038 |
| AL645608.6 | 0.826162 | 0.302515 | -1.44942 | 0.001466 | 0.003815 |
| KLK13 | 141.9477 | 66.69623 | -1.08968 | 0.001553 | 0.004008 |
| NMUR2 | 0.422504 | 0.157719 | -1.42161 | 0.001556 | 0.004015 |
| AC036108.1 | 0.355213 | 0.94672 | 1.414253 | 0.001589 | 0.004088 |
| TG | 1.099875 | 0.431744 | -1.34909 | 0.001607 | 0.004124 |
| CAPN9 | 0.61346 | 0.258134 | -1.24885 | 0.001609 | 0.004128 |
| RNF183 | 1.676427 | 0.487756 | -1.78116 | 0.001629 | 0.004166 |
| CLDN17 | 7.39096 | 2.84021 | -1.37976 | 0.001645 | 0.004202 |
| S100A7 | 3908.804 | 1879.892 | -1.05608 | 0.001655 | 0.004225 |
| PSORS1C2 | 18.41213 | 8.205673 | -1.16596 | 0.001747 | 0.004428 |
| PKDCC | 8.457675 | 2.881845 | -1.55327 | 0.001768 | 0.004476 |
| AL034376.1 | 5.588059 | 2.1844 | -1.35511 | 0.001774 | 0.004487 |
| TAC3 | 0.879677 | 0.095431 | -3.20445 | 0.001777 | 0.004494 |
| ALDOB | 0.318188 | 0.147588 | -1.1083 | 0.001832 | 0.004612 |
| ENDOU | 10.78452 | 4.559291 | -1.24208 | 0.001852 | 0.004654 |
| UBXN7-AS1 | 0.245043 | 0.122333 | -1.00222 | 0.001914 | 0.004792 |
| NOS2 | 8.286125 | 1.985068 | -2.06151 | 0.001992 | 0.004957 |
| OTOP1 | 0.291131 | 0.055436 | -2.39276 | 0.002006 | 0.004988 |
| NTS | 253.0466 | 95.8938 | -1.39989 | 0.002016 | 0.005011 |
| GALR2 | 1.869754 | 0.766301 | -1.28687 | 0.002085 | 0.005167 |
| YBX2 | 3.687561 | 1.12181 | -1.71684 | 0.002115 | 0.005231 |
| CRACD | 0.965741 | 0.450263 | -1.10087 | 0.002162 | 0.005327 |
| IGLV3-13 | 1.307172 | 0.478938 | -1.44854 | 0.002216 | 0.005435 |
| AL732314.4 | 0.245193 | 0.121712 | -1.01045 | 0.002284 | 0.005584 |
| B3GNT6 | 1.336209 | 0.508088 | -1.395 | 0.002321 | 0.005664 |
| DIRAS2 | 0.810409 | 0.125034 | -2.69632 | 0.002346 | 0.005717 |
| AL390778.2 | 1.550004 | 0.470571 | -1.71979 | 0.002386 | 0.0058 |
| AC120498.4 | 1.144031 | 0.558931 | -1.03338 | 0.002438 | 0.00591 |
| SYNGR3 | 5.453739 | 2.448306 | -1.15546 | 0.00256 | 0.006158 |
| SYT2 | 0.321163 | 0.095738 | -1.74614 | 0.002566 | 0.006172 |
| SDK1-AS1 | 0.428907 | 0.212192 | -1.01529 | 0.002629 | 0.006305 |
| BHMT | 0.387001 | 0.146553 | -1.40092 | 0.002641 | 0.006329 |
| ODAM | 33.18797 | 1.360279 | -4.60869 | 0.002737 | 0.006525 |
| AL354766.2 | 3.976775 | 1.916837 | -1.05287 | 0.002795 | 0.006652 |
| LINC02437 | 0.699253 | 0.315495 | -1.1482 | 0.002847 | 0.00676 |
| PTCH2 | 1.383437 | 0.458531 | -1.59316 | 0.00288 | 0.00683 |
| AP000696.1 | 0.431525 | 0.098093 | -2.13722 | 0.002915 | 0.006902 |
| LRRC4 | 22.61737 | 10.68495 | -1.08185 | 0.002948 | 0.006974 |
| AP000688.2 | 0.525478 | 0.190887 | -1.46091 | 0.002949 | 0.006974 |
| AC021683.1 | 0.43324 | 0.199278 | -1.12038 | 0.003051 | 0.007193 |
| LINC02006 | 0.703377 | 0.31311 | -1.16763 | 0.003077 | 0.007247 |
| CPLX2 | 2.011197 | 0.068818 | -4.86912 | 0.00313 | 0.007352 |
| RTL1 | 0.142232 | 0.796236 | 2.484952 | 0.003229 | 0.007557 |
| LINC02244 | 1.425983 | 0.707424 | -1.01131 | 0.003263 | 0.007624 |
| SYCP2L | 0.400551 | 0.179316 | -1.15948 | 0.003285 | 0.007669 |
| SOST | 5.089094 | 1.3923 | -1.86994 | 0.003295 | 0.00769 |
| SOWAHA | 0.667325 | 0.232382 | -1.52189 | 0.00337 | 0.007842 |
| HPN-AS1 | 0.45557 | 0.215236 | -1.08175 | 0.00346 | 0.008025 |
| SLC26A5 | 0.303469 | 0.120524 | -1.33223 | 0.003498 | 0.008101 |
| JAKMIP2 | 1.450639 | 0.516862 | -1.48884 | 0.003598 | 0.008297 |
| KRT3 | 7.868056 | 1.381202 | -2.51008 | 0.003623 | 0.008347 |
| FSTL4 | 5.952368 | 2.848418 | -1.0633 | 0.003631 | 0.008364 |
| G0S2 | 42.77215 | 114.2451 | 1.417388 | 0.003699 | 0.0085 |
| CD177P1 | 0.956916 | 0.208997 | -2.19491 | 0.003729 | 0.008561 |
| CRCT1 | 228.0882 | 90.16998 | -1.33887 | 0.003741 | 0.008586 |
| OR7E108P | 0.386748 | 0.192007 | -1.01023 | 0.003747 | 0.008599 |
| HCG22 | 0.961926 | 0.275255 | -1.80516 | 0.003769 | 0.00864 |
| CECR2 | 2.195936 | 0.785002 | -1.48407 | 0.003828 | 0.008756 |
| AL390718.1 | 0.323812 | 0.145843 | -1.15073 | 0.003911 | 0.008926 |
| RLN2 | 0.41984 | 0.191943 | -1.12916 | 0.003983 | 0.009067 |
| KLK6 | 150.8586 | 72.85336 | -1.05013 | 0.004164 | 0.009417 |
| TEX45 | 0.259051 | 0.128474 | -1.01176 | 0.004188 | 0.009465 |
| FMO9P | 0.567648 | 0.234451 | -1.27571 | 0.004198 | 0.009484 |
| GPD1 | 1.339213 | 0.657613 | -1.02608 | 0.004202 | 0.009492 |
| VWCE | 0.91931 | 0.40854 | -1.17007 | 0.004269 | 0.009625 |
| IL6 | 15.10174 | 31.46199 | 1.058895 | 0.004406 | 0.009897 |
| GPRC5D | 10.64325 | 3.760764 | -1.50084 | 0.004486 | 0.010055 |
| AL354754.1 | 0.966538 | 0.210239 | -2.2008 | 0.004488 | 0.010058 |
| SMC1B | 3.381631 | 1.248945 | -1.43701 | 0.004501 | 0.010083 |
| PAH | 0.268782 | 0.0237 | -3.5035 | 0.004513 | 0.010105 |
| TDRG1 | 0.681971 | 0.083087 | -3.03702 | 0.004554 | 0.010182 |
| AC023510.2 | 0.324451 | 0.134625 | -1.26906 | 0.004557 | 0.010185 |
| AL021026.1 | 0.241484 | 0.088756 | -1.44401 | 0.004653 | 0.010373 |
| AZGP1 | 25.66258 | 11.00333 | -1.22173 | 0.004702 | 0.010461 |
| NRXN3 | 0.608586 | 0.19961 | -1.60828 | 0.004745 | 0.010541 |
| TLL2 | 0.662016 | 1.380116 | 1.059851 | 0.004917 | 0.010867 |
| C9orf152 | 1.022492 | 0.445786 | -1.19767 | 0.005028 | 0.011086 |
| ACADL | 0.561573 | 0.160694 | -1.80516 | 0.005078 | 0.01118 |
| PLCH1 | 1.021958 | 0.436029 | -1.22884 | 0.00509 | 0.011203 |
| ARHGEF33 | 0.407646 | 0.202166 | -1.01178 | 0.005135 | 0.011291 |
| GPC3 | 48.96804 | 13.33985 | -1.8761 | 0.005164 | 0.011342 |
| PRRG3 | 1.032306 | 0.247296 | -2.06156 | 0.005255 | 0.01152 |
| AC009005.1 | 3.35026 | 1.604303 | -1.06233 | 0.005292 | 0.011589 |
| BEST2 | 2.027794 | 0.816024 | -1.31323 | 0.005312 | 0.011626 |
| STK33 | 0.374361 | 0.176264 | -1.08669 | 0.005315 | 0.011631 |
| ELAPOR1 | 6.759646 | 2.163241 | -1.64375 | 0.005315 | 0.011631 |
| ADRA2B | 1.807175 | 0.626243 | -1.52894 | 0.005339 | 0.011674 |
| FRMD1 | 0.544939 | 0.047307 | -3.52596 | 0.00537 | 0.011737 |
| METTL24 | 0.779667 | 0.246713 | -1.66002 | 0.005416 | 0.011824 |
| PTPRT | 0.420864 | 0.096656 | -2.12242 | 0.005431 | 0.011853 |
| AKR1B10P1 | 4.101929 | 1.638503 | -1.32392 | 0.005584 | 0.01214 |
| TF | 3.833655 | 1.612435 | -1.24948 | 0.005729 | 0.012408 |
| TDRD12 | 1.120832 | 0.494491 | -1.18055 | 0.005792 | 0.012526 |
| AL359715.2 | 0.30917 | 0.154202 | -1.00358 | 0.00581 | 0.012558 |
| NAP1L6P | 0.261021 | 0.050362 | -2.37376 | 0.005893 | 0.012717 |
| FOXI2 | 0.350778 | 0.138512 | -1.34055 | 0.005901 | 0.012731 |
| KRT8P40 | 0.615996 | 0.107528 | -2.51821 | 0.005952 | 0.012828 |
| MUC3A | 0.464215 | 0.930931 | 1.00388 | 0.005986 | 0.012885 |
| AL121601.1 | 1.176421 | 0.563748 | -1.06128 | 0.006043 | 0.012989 |
| AP001574.1 | 0.851303 | 0.250296 | -1.76604 | 0.00606 | 0.013022 |
| ENPP6 | 1.166243 | 0.444705 | -1.39095 | 0.00613 | 0.013157 |
| FATE1 | 0.798368 | 0.319003 | -1.32348 | 0.006184 | 0.01326 |
| TAF7L | 2.506108 | 0.263848 | -3.24767 | 0.006222 | 0.013329 |
| OR7E161P | 0.334507 | 0.146069 | -1.19538 | 0.006283 | 0.013447 |
| AKR1C6P | 0.252602 | 0.099029 | -1.35094 | 0.006413 | 0.013682 |
| PSG4 | 1.047824 | 3.26082 | 1.637838 | 0.006537 | 0.013897 |
| MUC6 | 1.081881 | 0.033924 | -4.99508 | 0.006542 | 0.013907 |
| AC011297.1 | 1.347256 | 0.383935 | -1.81109 | 0.00655 | 0.013918 |
| AZGP1P1 | 0.34829 | 0.11579 | -1.58878 | 0.006571 | 0.013951 |
| SLC2A4 | 1.82806 | 0.799746 | -1.1927 | 0.006631 | 0.014061 |
| TMSB15A | 8.01686 | 2.171601 | -1.88428 | 0.006768 | 0.01431 |
| DSG4 | 1.04914 | 0.259129 | -2.01747 | 0.006799 | 0.01437 |
| AL138916.1 | 0.55757 | 0.262094 | -1.08907 | 0.006834 | 0.014429 |
| EWSAT1 | 0.560587 | 0.184986 | -1.59952 | 0.006927 | 0.014599 |
| SERPINA12 | 0.657189 | 0.299004 | -1.13615 | 0.006961 | 0.014661 |
| GPHA2 | 0.448912 | 0.136845 | -1.7139 | 0.006962 | 0.014661 |
| SLC5A8 | 0.328528 | 0.130607 | -1.33079 | 0.007441 | 0.015541 |
| RPS6KA6 | 0.652846 | 0.321136 | -1.02356 | 0.007443 | 0.015544 |
| RAMP2-AS1 | 0.665547 | 0.280215 | -1.24801 | 0.007448 | 0.015548 |
| HMGCS2 | 0.690747 | 0.286809 | -1.26807 | 0.007513 | 0.015663 |
| DMRT3 | 2.214188 | 0.716626 | -1.62748 | 0.007649 | 0.015913 |
| LINC00052 | 0.272432 | 0.588395 | 1.110891 | 0.007689 | 0.01598 |
| CPEB1 | 0.585539 | 0.223052 | -1.39238 | 0.007874 | 0.016322 |
| DRD1 | 0.333515 | 0.094766 | -1.81532 | 0.007989 | 0.01653 |
| IL13RA2 | 3.37947 | 14.86322 | 2.136877 | 0.008103 | 0.016716 |
| AC106820.4 | 0.562061 | 0.209159 | -1.42613 | 0.00818 | 0.016849 |
| AC026336.3 | 0.942992 | 0.152852 | -2.62511 | 0.00819 | 0.016865 |
| LDHC | 1.006368 | 0.444397 | -1.17924 | 0.008242 | 0.016945 |
| KASH5 | 0.332296 | 0.066028 | -2.33131 | 0.008948 | 0.018199 |
| C4BPAP2 | 0.436029 | 0.205778 | -1.08334 | 0.009147 | 0.018547 |
| CEND1 | 1.947178 | 0.686273 | -1.50453 | 0.009263 | 0.018743 |
| CCKBR | 0.365056 | 0.082745 | -2.14138 | 0.009322 | 0.018846 |
| DNAJB3 | 0.267926 | 0.114295 | -1.22907 | 0.009484 | 0.019123 |
| AL049757.1 | 0.379791 | 0.075692 | -2.327 | 0.009728 | 0.019565 |
| GABRA5 | 0.468835 | 0.030684 | -3.9335 | 0.009815 | 0.019712 |
| NDP | 0.728814 | 0.341537 | -1.09351 | 0.009846 | 0.019766 |
| STOX1 | 3.019155 | 1.235883 | -1.2886 | 0.00986 | 0.019786 |
| PLA2G1B | 0.292945 | 0.093885 | -1.64166 | 0.009931 | 0.019902 |
| VTCN1 | 8.164978 | 3.725606 | -1.13197 | 0.009939 | 0.019916 |
| AP000697.1 | 0.645354 | 0.196021 | -1.71908 | 0.009951 | 0.019937 |
| CEACAM16 | 0.458535 | 0.180059 | -1.34856 | 0.009999 | 0.020018 |
| TUBB2B | 11.27087 | 4.849295 | -1.21675 | 0.010005 | 0.020025 |
| CELF4 | 0.282379 | 0.109029 | -1.37291 | 0.010104 | 0.020193 |
| LINC02875 | 1.165623 | 0.521839 | -1.15943 | 0.01013 | 0.020229 |
| SYCP2 | 5.746275 | 1.815816 | -1.66201 | 0.010193 | 0.02034 |
| KRT83 | 0.37705 | 0.07299 | -2.36898 | 0.010493 | 0.020875 |
| SPINK9 | 0.512103 | 0.149202 | -1.77917 | 0.010769 | 0.021361 |
| KCNB2 | 0.608845 | 0.101221 | -2.58857 | 0.010967 | 0.021683 |
| RIPPLY3 | 2.335447 | 1.046376 | -1.1583 | 0.010976 | 0.021697 |
| AL358394.1 | 1.091942 | 0.468244 | -1.22156 | 0.010979 | 0.021697 |
| ZNF157 | 0.284651 | 0.120061 | -1.24543 | 0.011086 | 0.021885 |
| TCIM | 47.08026 | 18.32993 | -1.36092 | 0.01116 | 0.022001 |
| QRFPR | 3.399894 | 1.511684 | -1.16933 | 0.011241 | 0.022143 |
| MIR9-3HG | 5.201169 | 2.073531 | -1.32675 | 0.011275 | 0.022203 |
| CYP2A6 | 0.351054 | 0.108388 | -1.69549 | 0.011519 | 0.022624 |
| FBLL1 | 0.944372 | 0.469658 | -1.00775 | 0.011621 | 0.022797 |
| ZIC1 | 1.947466 | 0.79279 | -1.29659 | 0.011729 | 0.022985 |
| AC126177.5 | 0.596385 | 0.227854 | -1.38813 | 0.012351 | 0.024055 |
| AC106795.3 | 0.386926 | 0.153858 | -1.33046 | 0.012485 | 0.024289 |
| VCAM1 | 27.37851 | 8.26313 | -1.72828 | 0.012661 | 0.024588 |
| OR7E47P | 0.466821 | 0.229901 | -1.02186 | 0.01316 | 0.025436 |
| DUOX2 | 22.52405 | 10.70751 | -1.07284 | 0.013288 | 0.025641 |
| NEUROD2 | 0.378227 | 0.125046 | -1.59679 | 0.01332 | 0.025689 |
| PENK | 2.457155 | 0.541403 | -2.18221 | 0.013664 | 0.026279 |
| MUC5AC | 1.359301 | 0.674062 | -1.01191 | 0.013709 | 0.026352 |
| CLGN | 3.617385 | 0.818357 | -2.14414 | 0.013869 | 0.026606 |
| IL17REL | 2.572419 | 0.111639 | -4.52621 | 0.014807 | 0.028166 |
| WNK3 | 0.502077 | 0.232734 | -1.10922 | 0.01483 | 0.028203 |
| COL4A4 | 1.963415 | 0.647545 | -1.60031 | 0.01486 | 0.028251 |
| IRX1 | 3.248134 | 7.414051 | 1.190651 | 0.014984 | 0.028463 |
| NKAIN2 | 1.813954 | 0.82376 | -1.13884 | 0.015767 | 0.029731 |
| ASPRV1 | 78.55651 | 30.94796 | -1.34389 | 0.01586 | 0.029889 |
| BNC2-AS1 | 0.940739 | 0.398026 | -1.24093 | 0.016297 | 0.03058 |
| GSTM1 | 50.50378 | 19.87829 | -1.3452 | 0.016951 | 0.03168 |
| ABCA13 | 6.309794 | 2.811649 | -1.16618 | 0.017053 | 0.031852 |
| DIO3 | 1.532015 | 3.211679 | 1.067897 | 0.017251 | 0.032178 |
| FAM163B | 0.372875 | 0.170737 | -1.12691 | 0.017399 | 0.032423 |
| UBBP3 | 0.276791 | 0.655763 | 1.244377 | 0.017853 | 0.033144 |
| FAM182A | 0.255754 | 0.094984 | -1.429 | 0.017878 | 0.033188 |
| AC012501.2 | 0.27336 | 0.086445 | -1.66094 | 0.01905 | 0.03506 |
| C16orf89 | 0.75608 | 0.338517 | -1.15931 | 0.019063 | 0.035079 |
| AL049874.3 | 0.664879 | 0.284126 | -1.22656 | 0.019377 | 0.035582 |
| AC010273.3 | 0.44437 | 0.198372 | -1.16356 | 0.019454 | 0.035708 |
| TCAM1P | 2.258575 | 0.774409 | -1.54424 | 0.019854 | 0.036328 |
| AC104964.3 | 0.43577 | 0.152214 | -1.51747 | 0.020697 | 0.037689 |
| MLXIPL | 1.283898 | 0.616324 | -1.05877 | 0.020715 | 0.037719 |
| ZIC4 | 0.240014 | 0.100804 | -1.25157 | 0.021132 | 0.038354 |
| CHCHD2P4 | 0.579878 | 0.230124 | -1.33334 | 0.021259 | 0.038562 |
| SIGLEC11 | 0.595617 | 0.25807 | -1.20663 | 0.021285 | 0.038594 |
| SLC8A2 | 0.992539 | 0.404078 | -1.29649 | 0.021393 | 0.038765 |
| SERPINA9 | 1.086346 | 0.44011 | -1.30355 | 0.021538 | 0.038989 |
| ATP2B2 | 0.240513 | 0.102186 | -1.23492 | 0.021758 | 0.039345 |
| AL138760.1 | 0.721385 | 0.360109 | -1.00233 | 0.022075 | 0.03985 |
| PGPEP1L | 0.574708 | 0.272728 | -1.07537 | 0.02217 | 0.039988 |
| GDF10 | 0.958647 | 0.477622 | -1.00513 | 0.022366 | 0.040274 |
| GJB1 | 0.647345 | 0.23528 | -1.46016 | 0.022649 | 0.040691 |
| CYP11A1 | 1.532197 | 0.490036 | -1.64464 | 0.022917 | 0.04109 |
| KCNH6 | 0.556396 | 0.023861 | -4.5434 | 0.023135 | 0.041404 |
| SST | 1.354143 | 0.593125 | -1.19097 | 0.023144 | 0.041416 |
| FLG2 | 3.211592 | 1.266404 | -1.34255 | 0.023397 | 0.041806 |
| AL022316.1 | 1.12325 | 0.531095 | -1.08064 | 0.02348 | 0.041935 |
| PPP1R1B | 5.073175 | 1.632322 | -1.63596 | 0.023987 | 0.042708 |
| COL19A1 | 0.58208 | 0.072603 | -3.00311 | 0.02423 | 0.043079 |
| AC099791.2 | 0.291806 | 0.139467 | -1.06508 | 0.024477 | 0.043461 |
| BRD9P2 | 0.921736 | 0.425132 | -1.11644 | 0.024702 | 0.043809 |
| TBL1Y | 0.513617 | 0.142819 | -1.8465 | 0.025543 | 0.045125 |
| CHRNB2 | 0.465531 | 0.136279 | -1.77231 | 0.026037 | 0.045873 |
| ARNT2 | 7.21343 | 3.072269 | -1.23138 | 0.026234 | 0.046167 |
| AC004540.2 | 1.056878 | 0.229253 | -2.2048 | 0.026404 | 0.046428 |
| ROPN1B | 0.282844 | 0.101623 | -1.47678 | 0.027168 | 0.047549 |
| HOXB13 | 2.90248 | 1.177195 | -1.30193 | 0.027211 | 0.047613 |
| MYEF2 | 0.598391 | 0.277128 | -1.11053 | 0.027976 | 0.048764 |
| ADGRG7 | 1.116297 | 0.052226 | -4.4178 | 0.027982 | 0.048771 |
| AC138649.1 | 0.28279 | 0.098771 | -1.51757 | 0.02802 | 0.048833 |
