# Supplementary material for: Relationships of Ferroptosis and Pyroptosis-Related Genes with Clinical Prognosis and Tumor Immune Microenvironment in Head and Neck Squamous Cell Carcinoma
Source: Oxid Med Cell Longev. 2022 Oct 5;2022:3713929. doi: 10.1155/2022/3713929 (PMC9557253; doi:10.1155/2022/3713929)
Supplement: Supplementary 6 — Supplementary Table 5. One hundred and sixty-five prognostic DEGs were obtained by univariate Cox regression analysis in TCGA-HNSC dataset. [file 3713929.f6.DOCX]

Supplementary table 5. one hundred and sixty-five prognostic DEGs was obtained by univariate Cox regression analysis in TCGA-HNSC dataset.

| id | HR | HR.95L | HR.95H | pvalue |
| --- | --- | --- | --- | --- |
| FOLR3 | 1.006108 | 1.003438 | 1.008785 | 7.09E-06 |
| SFTA1P | 1.023477 | 1.012315 | 1.034763 | 3.36E-05 |
| LRATD1 | 0.985789 | 0.978929 | 0.992697 | 5.89E-05 |
| MYO7B | 1.040744 | 1.018808 | 1.063151 | 0.000238 |
| NPW | 1.005863 | 1.00266 | 1.009076 | 0.000328 |
| MAEL | 1.027043 | 1.011697 | 1.042622 | 0.000513 |
| AC002066.1 | 1.148761 | 1.061832 | 1.242805 | 0.000552 |
| AC004540.2 | 1.015803 | 1.006742 | 1.024946 | 0.000604 |
| AC006159.1 | 1.506247 | 1.189716 | 1.906995 | 0.000666 |
| CLDN6 | 1.015745 | 1.006509 | 1.025066 | 0.000802 |
| LINC01704 | 1.266895 | 1.102635 | 1.455624 | 0.000841 |
| MCHR1 | 1.07794 | 1.030961 | 1.127059 | 0.000963 |
| CCBE1 | 1.069111 | 1.026795 | 1.113171 | 0.001182 |
| AC104461.1 | 1.171438 | 1.062604 | 1.291418 | 0.00147 |
| PXN | 1.004131 | 1.001571 | 1.006697 | 0.001548 |
| ITGB1-DT | 1.091164 | 1.033565 | 1.151974 | 0.001615 |
| LINC01356 | 1.179605 | 1.062532 | 1.309577 | 0.001953 |
| INSM1 | 1.006903 | 1.002517 | 1.011309 | 0.002013 |
| SUN3 | 1.04235 | 1.015261 | 1.070162 | 0.00202 |
| MYOSLID | 1.00978 | 1.003446 | 1.016154 | 0.002435 |
| OLFM1 | 0.991384 | 0.985827 | 0.996972 | 0.002548 |
| ITGA6 | 1.000476 | 1.000162 | 1.000791 | 0.002976 |
| KCNH6 | 1.016237 | 1.005493 | 1.027096 | 0.002977 |
| PDCL2 | 1.107459 | 1.035 | 1.18499 | 0.003112 |
| ACADL | 1.032806 | 1.010387 | 1.055723 | 0.003942 |
| CARD10 | 1.007344 | 1.002335 | 1.012377 | 0.004014 |
| NKAIN2 | 0.91588 | 0.862503 | 0.972561 | 0.004129 |
| LRRN4 | 1.072639 | 1.022438 | 1.125305 | 0.004139 |
| IL13RA2 | 1.003781 | 1.001192 | 1.006377 | 0.004188 |
| ZDHHC11B | 1.011554 | 1.003568 | 1.019603 | 0.004501 |
| CAV1 | 1.000512 | 1.000157 | 1.000866 | 0.004654 |
| PPM1L | 0.953135 | 0.9218 | 0.985536 | 0.00489 |
| SAMD12 | 0.969294 | 0.948208 | 0.990849 | 0.005449 |
| NPM1P25 | 0.913952 | 0.857279 | 0.974373 | 0.005872 |
| UBASH3A | 0.911398 | 0.853132 | 0.973645 | 0.005917 |
| DDC | 1.023717 | 1.006634 | 1.04109 | 0.006331 |
| AL596223.1 | 1.066732 | 1.018386 | 1.117374 | 0.006336 |
| PRELP | 0.988342 | 0.980044 | 0.996709 | 0.006406 |
| PDZK1 | 1.0715 | 1.019575 | 1.126069 | 0.006433 |
| LINC01357 | 1.072497 | 1.019459 | 1.128293 | 0.006836 |
| BRD9P2 | 1.032664 | 1.008826 | 1.057064 | 0.006988 |
| OR2I1P | 0.995228 | 0.991741 | 0.998727 | 0.00756 |
| DUX4L27 | 1.236427 | 1.056145 | 1.447484 | 0.008308 |
| TRAV20 | 0.819866 | 0.707371 | 0.950252 | 0.008349 |
| SH2D5 | 1.009788 | 1.002435 | 1.017194 | 0.008997 |
| ADCY6-DT | 1.195001 | 1.045469 | 1.365921 | 0.009004 |
| AC128687.2 | 1.620375 | 1.126067 | 2.33167 | 0.009339 |
| TRBV7-4 | 0.644945 | 0.463297 | 0.897811 | 0.009359 |
| LINC02535 | 1.191671 | 1.043459 | 1.360936 | 0.009661 |
| AREG | 1.000883 | 1.000213 | 1.001552 | 0.009743 |
| RLN2 | 1.055869 | 1.013178 | 1.10036 | 0.009833 |
| AL109914.1 | 0.797295 | 0.671017 | 0.947339 | 0.010027 |
| NUP210 | 0.994317 | 0.989992 | 0.998661 | 0.010399 |
| AC084816.1 | 1.099655 | 1.0223 | 1.182862 | 0.010693 |
| NRG1 | 1.005575 | 1.001283 | 1.009884 | 0.010842 |
| CAV2 | 1.003818 | 1.000879 | 1.006766 | 0.010854 |
| TRAV18 | 0.559756 | 0.357597 | 0.876202 | 0.011148 |
| FEZ1 | 1.006757 | 1.001509 | 1.012033 | 0.011554 |
| ADGRG7 | 1.013452 | 1.002962 | 1.024052 | 0.011835 |
| IFNG-AS1 | 0.639831 | 0.450259 | 0.909217 | 0.012745 |
| KRT24 | 1.000917 | 1.000191 | 1.001644 | 0.013286 |
| UBD | 0.996809 | 0.994282 | 0.999343 | 0.013603 |
| ZNF831 | 0.784814 | 0.647284 | 0.951564 | 0.0137 |
| LY9 | 0.840864 | 0.732041 | 0.965865 | 0.014241 |
| CAMK2N1 | 1.004447 | 1.000887 | 1.008019 | 0.014301 |
| LINC02384 | 0.77384 | 0.629823 | 0.950787 | 0.014676 |
| CD52 | 0.996826 | 0.994278 | 0.999379 | 0.014871 |
| AL161431.1 | 1.00318 | 1.000607 | 1.005759 | 0.015388 |
| AC084819.1 | 1.054502 | 1.009823 | 1.101158 | 0.016283 |
| AC009005.1 | 1.02675 | 1.004794 | 1.049186 | 0.016684 |
| CCKAR | 0.874646 | 0.783701 | 0.976144 | 0.016802 |
| LINC01116 | 1.017076 | 1.003054 | 1.031294 | 0.016825 |
| SPRR3 | 1.000052 | 1.000009 | 1.000096 | 0.017537 |
| GABRP | 0.996663 | 0.993908 | 0.999425 | 0.017934 |
| LINC00330 | 1.175843 | 1.028141 | 1.344763 | 0.018021 |
| TMEM150C | 0.980643 | 0.964802 | 0.996745 | 0.018653 |
| AL121601.1 | 1.034063 | 1.005581 | 1.063352 | 0.018747 |
| GPR39 | 1.032506 | 1.005316 | 1.060432 | 0.018805 |
| TACR1 | 0.780074 | 0.633281 | 0.960893 | 0.019543 |
| TRBV10-2 | 0.785601 | 0.641504 | 0.962065 | 0.019594 |
| GPR18 | 0.891962 | 0.809731 | 0.982544 | 0.020514 |
| GATM | 0.978656 | 0.960953 | 0.996684 | 0.020527 |
| LRRC37A14P | 0.536342 | 0.31601 | 0.910298 | 0.020989 |
| AC124276.2 | 1.242312 | 1.032984 | 1.494059 | 0.021186 |
| PRKAR2B | 0.961245 | 0.929455 | 0.994122 | 0.021247 |
| CYP4F29P | 1.027084 | 1.003986 | 1.050714 | 0.021295 |
| LAMC2 | 1.000156 | 1.000022 | 1.00029 | 0.02208 |
| GJB1 | 1.054502 | 1.007633 | 1.103552 | 0.02215 |
| SLC16A2 | 1.008304 | 1.001178 | 1.015481 | 0.022288 |
| LINC02006 | 0.833202 | 0.712332 | 0.974582 | 0.022494 |
| E2F6P4 | 1.119259 | 1.015999 | 1.233013 | 0.022528 |
| NT5E | 1.002772 | 1.00038 | 1.00517 | 0.023122 |
| PLA2G3 | 0.988842 | 0.979271 | 0.998507 | 0.023753 |
| VPREB3 | 0.976047 | 0.955709 | 0.996819 | 0.024039 |
| CYP27A1 | 0.995074 | 0.990783 | 0.999383 | 0.02511 |
| TRBJ2-2 | 0.914428 | 0.845498 | 0.988979 | 0.025279 |
| BNC2-AS1 | 1.043984 | 1.005334 | 1.08412 | 0.025327 |
| OR10Y1P | 1.082101 | 1.009766 | 1.159618 | 0.025398 |
| CSMD1 | 1.1236 | 1.014448 | 1.244497 | 0.025412 |
| MYH14 | 0.99744 | 0.995196 | 0.999688 | 0.025656 |
| ILDR1 | 0.983909 | 0.969959 | 0.998059 | 0.025974 |
| VEGFC | 1.003513 | 1.00041 | 1.006626 | 0.026468 |
| TRBC2 | 0.995552 | 0.991638 | 0.999482 | 0.026555 |
| FST | 1.001016 | 1.000115 | 1.001918 | 0.027023 |
| AL365361.1 | 0.916575 | 0.848256 | 0.990397 | 0.027516 |
| DOC2B | 0.993698 | 0.988104 | 0.999324 | 0.028179 |
| PDGFC | 1.009613 | 1.001024 | 1.018276 | 0.028184 |
| GZMM | 0.975577 | 0.954245 | 0.997386 | 0.028377 |
| MIXL1 | 0.855183 | 0.743358 | 0.983831 | 0.028673 |
| CYP4X1 | 0.992376 | 0.985593 | 0.999205 | 0.028722 |
| INPP4B | 1.027428 | 1.002753 | 1.05271 | 0.029135 |
| TRBV18 | 0.925604 | 0.863262 | 0.992449 | 0.029779 |
| LINC02487 | 1.019782 | 1.001887 | 1.037996 | 0.030104 |
| ENPP6 | 0.915256 | 0.844859 | 0.99152 | 0.030119 |
| TMPRSS11B | 1.005277 | 1.00048 | 1.010097 | 0.031054 |
| CD27 | 0.990759 | 0.982415 | 0.999174 | 0.031442 |
| FTH1P22 | 0.92877 | 0.86827 | 0.993485 | 0.031542 |
| STAP1 | 0.936398 | 0.881937 | 0.994222 | 0.031593 |
| AC117422.1 | 1.315334 | 1.023358 | 1.690614 | 0.032334 |
| PIP5K1B | 1.025263 | 1.001973 | 1.049095 | 0.033328 |
| LINP1 | 1.0073 | 1.000574 | 1.014071 | 0.03335 |
| TRBV7-6 | 0.91345 | 0.840188 | 0.9931 | 0.033814 |
| UGT1A7 | 0.991003 | 0.982734 | 0.999341 | 0.034506 |
| AKNAD1 | 1.149961 | 1.009481 | 1.309989 | 0.03556 |
| SCML4 | 0.760388 | 0.588914 | 0.98179 | 0.035648 |
| FSTL3 | 1.001774 | 1.000117 | 1.003433 | 0.035816 |
| AC004808.2 | 0.771497 | 0.605364 | 0.983222 | 0.036019 |
| AC002401.4 | 1.013188 | 1.000815 | 1.025715 | 0.036629 |
| SYNGR1 | 0.977156 | 0.956185 | 0.998587 | 0.036822 |
| ALDOB | 1.159599 | 1.008641 | 1.33315 | 0.037445 |
| REN | 1.129039 | 1.006991 | 1.265879 | 0.037587 |
| PCSK2 | 1.008733 | 1.000433 | 1.017103 | 0.039156 |
| CHL1 | 0.98373 | 0.968508 | 0.999191 | 0.03924 |
| CSF3 | 1.001895 | 1.000093 | 1.0037 | 0.039319 |
| HAMP | 0.755449 | 0.578445 | 0.986616 | 0.039505 |
| AC124276.1 | 1.096724 | 1.004179 | 1.197798 | 0.040103 |
| TRBV23-1 | 0.762816 | 0.588951 | 0.988007 | 0.040229 |
| GSDME | 1.009842 | 1.000434 | 1.019339 | 0.040293 |
| LINC02448 | 1.085366 | 1.003573 | 1.173826 | 0.040444 |
| AC104024.2 | 1.270273 | 1.01036 | 1.597046 | 0.040539 |
| LAMB3 | 1.000225 | 1.000009 | 1.00044 | 0.041131 |
| ELAPOR1 | 0.985773 | 0.972286 | 0.999448 | 0.041496 |
| SPRR2D | 1.000108 | 1.000004 | 1.000213 | 0.041864 |
| NAV3 | 1.052607 | 1.001879 | 1.105904 | 0.041906 |
| KRT77 | 0.950369 | 0.90485 | 0.998178 | 0.042074 |
| AC078909.2 | 1.212583 | 1.006713 | 1.460552 | 0.042311 |
| DSG4 | 0.84642 | 0.720358 | 0.994543 | 0.042717 |
| AL390778.2 | 0.95946 | 0.921811 | 0.998646 | 0.042734 |
| FALEC | 1.045785 | 1.001424 | 1.092111 | 0.042939 |
| RNU1-72P | 1.153418 | 1.00448 | 1.324439 | 0.043039 |
| CD19 | 0.973644 | 0.948769 | 0.999172 | 0.043102 |
| MANCR | 1.033443 | 1.000796 | 1.067155 | 0.044585 |
| NMU | 1.001616 | 1.000038 | 1.003197 | 0.044767 |
| IKZF3 | 0.979442 | 0.959769 | 0.999517 | 0.044791 |
| CLEC4O | 1.038805 | 1.000878 | 1.078169 | 0.044834 |
| MGAT3 | 0.9893 | 0.97894 | 0.99977 | 0.045203 |
| TMEM200C | 0.797305 | 0.6377 | 0.996857 | 0.046857 |
| ST8SIA6 | 0.833525 | 0.696469 | 0.997553 | 0.046956 |
| AC010343.3 | 1.027425 | 1.000312 | 1.055274 | 0.04739 |
| SUSD4 | 0.99452 | 0.989128 | 0.999941 | 0.047557 |
| PRSS23 | 1.003082 | 1.000028 | 1.006145 | 0.047927 |
| FCRL1 | 0.821592 | 0.675779 | 0.998867 | 0.048686 |
| APCDD1L | 1.014674 | 1.00007 | 1.029491 | 0.048905 |
| LINC00861 | 0.833478 | 0.695162 | 0.999315 | 0.049141 |
| AL035661.1 | 0.993338 | 0.986728 | 0.999992 | 0.049723 |
