# Supplementary material for: Relationships of Ferroptosis and Pyroptosis-Related Genes with Clinical Prognosis and Tumor Immune Microenvironment in Head and Neck Squamous Cell Carcinoma
Source: Oxid Med Cell Longev. 2022 Oct 5;2022:3713929. doi: 10.1155/2022/3713929 (PMC9557253; doi:10.1155/2022/3713929)
Supplement: Supplementary 7 — Supplementary Table 6. GO enrichment analysis based on the 165 prognostic DEGs using the “clusterProfiler” R package. [file 3713929.f7.DOCX]

Supplementary table 6. GO enrichment analysis based on the 165 prognostic DEGs using the “clusterProfiler” R package.

| Term | Count | PValue | Genes | Benjamini | FDR |
| --- | --- | --- | --- | --- | --- |
| GO:0005198~structural molecule activity | 12 | 2.91E-07 | SPRR3, LCE1B, CLDN10, CLDN3, KRT4, KRT3, KRT13, KRT24, KRT78, NEFH, KRT40, KRT83 | 6.97E-05 | 6.97E-05 |
| GO:0005882~intermediate filament | 7 | 7.70E-05 | KRT4, KRT3, KRT2, KRT36, KRT13, KRT24, KRT40 | 0.00480791 | 0.004682486 |
| GO:0005615~extracellular space | 22 | 8.36E-05 | PCSK2, IGHM, SERPINB12, ODAM, KRT2, ABCA3, KRT78, OTOP1, ELFN2, KRT83, FGF5, C1QTNF4, CHIT1, IL36A, EPYC, SCGB2A1, IFNK, FAM3B, SERPINI2, TAC3, IL13RA2, INA | 0.00480791 | 0.004682486 |
| GO:0018149~peptide cross-linking | 5 | 2.48E-04 | SPRR3, LCE1B, TGM7, TGM6, TGM3 | 0.114117391 | 0.114117391 |
| GO:0045111~intermediate filament cytoskeleton | 5 | 3.12E-04 | KRT4, KRT2, KRT13, KRT24, INA | 0.011832429 | 0.011523757 |
| GO:0045095~keratin filament | 6 | 4.12E-04 | KRT4, KRT3, KRT2, KRT13, KRT78, KRT83 | 0.011832429 | 0.011523757 |
| GO:0003810~protein-glutamine gamma-glutamyltransferase activity | 3 | 0.001266015 | TGM7, TGM6, TGM3 | 0.15192176 | 0.15192176 |
| GO:0031424~keratinization | 4 | 0.003173005 | SPRR3, LCE1B, KRT2, TGM3 | 0.494431856 | 0.494431856 |
| GO:0006958~complement activation, classical pathway | 5 | 0.003217561 | IGHM, C8G, IGKV1-17, IGKV1D-39, IGKV1D-12 | 0.494431856 | 0.494431856 |
| GO:0070062~extracellular exosome | 30 | 0.003710635 | CRB2, SPRR3, IGHM, DDC, SERPINB12, KRT24, ITLN1, TMPRSS2, FUT6, C8G, IGKV1-17, FAM3B, TAC3, ARSF, TGM3, CD177, TMPRSS11B, KRT3, KRT2, LRRN4, KRT13, KRT78, UPB1, IGKV1D-12, CRNN, CLDN3, PAH, KRT36, SERPINI2, MUC21 | 0.085344616 | 0.083118235 |
| GO:0005576~extracellular region | 20 | 0.005118921 | COL26A1, SPINK7, ODAM, IGKV1D-39, COL19A1, IGKV1D-12, FGF5, CHIT1, PRRG3, IL36A, C8G, IGKV1-17, IGKV2-28, PENK, IFNK, FAM3B, FDCSP, TAC3, IL13RA2, MUC6 | 0.098112645 | 0.095553184 |
| GO:0004497~monooxygenase activity | 4 | 0.005194622 | PAH, CYP4F3, CYP2E1, FMO3 | 0.415569725 | 0.415569725 |
| GO:0030216~keratinocyte differentiation | 4 | 0.011399183 | SPRR3, LCE1B, DSG4, TGM3 | 1 | 1 |
| GO:0008544~epidermis development | 4 | 0.015395798 | SPRR3, DCT, KRT2, KRT83 | 1 | 1 |
| GO:0004252~serine-type endopeptidase activity | 6 | 0.019262882 | PCSK2, TMPRSS11B, IGKV1-17, TMPRSS2, IGKV1D-39, IGKV1D-12 | 0.980514257 | 0.980514257 |
| GO:0004867~serine-type endopeptidase inhibitor activity | 4 | 0.020969915 | SERPINB12, SPINK7, TFPI2, SERPINI2 | 0.980514257 | 0.980514257 |
| GO:0003823~antigen binding | 4 | 0.024512856 | IGHM, IGKV1-17, IGKV1D-39, IGKV1D-12 | 0.980514257 | 0.980514257 |
| GO:0032280~symmetric synapse | 2 | 0.024790161 | PENK, NTSR1 | 0.407266936 | 0.396642581 |
| GO:0043204~perikaryon | 4 | 0.028890228 | PCSK2, KCNB2, PENK, NTSR1 | 0.415297029 | 0.404463193 |
| GO:0042423~catecholamine biosynthetic process | 2 | 0.036248783 | DDC, PAH | 1 | 1 |
| GO:0001696~gastric acid secretion | 2 | 0.036248783 | SLC9A4, CCKBR | 1 | 1 |
| GO:0010951~negative regulation of endopeptidase activity | 4 | 0.038372845 | CRB2, SERPINB12, TFPI2, SERPINI2 | 1 | 1 |
| GO:0071805~potassium ion transmembrane transport | 4 | 0.038372845 | SLC9A2, SLC9A4, KCNH6, KCNB2 | 1 | 1 |
| GO:0019221~cytokine-mediated signaling pathway | 4 | 0.046722335 | IL17REL, IL36A, IFNK, IL13RA2 | 1 | 1 |
| GO:0005883~neurofilament | 2 | 0.048971029 | NEFH, INA | 0.625740921 | 0.609417245 |
