# Supplementary material for: Relationships of Ferroptosis and Pyroptosis-Related Genes with Clinical Prognosis and Tumor Immune Microenvironment in Head and Neck Squamous Cell Carcinoma
Source: Oxid Med Cell Longev. 2022 Oct 5;2022:3713929. doi: 10.1155/2022/3713929 (PMC9557253; doi:10.1155/2022/3713929)
Supplement: Supplementary 8 — Supplementary Table 7. Strong positive correlation and mutual regulation between the 22 types of immune cells (except CD56dim.natural.killer.cellna). [file 3713929.f8.docx]

Supplementary table 7. Strong positive correlation and mutual regulation between the 22 types of immune cells (Except CD56dim.natural.killer.cellna).

| from | to | cor | pvalue | weight |
| --- | --- | --- | --- | --- |
| Activated.B.cellna | Activated.CD4.T.cellna | 0.721817 | 5.92E-82 | 4.330904 |
| Activated.B.cellna | Activated.CD8.T.cellna | 0.745569 | 3.60E-90 | 4.473412 |
| Activated.B.cellna | Activated.dendritic.cellna | 0.602779 | 5.58E-51 | 3.616675 |
| Activated.B.cellna | CD56bright.natural.killer.cellna | 0.222186 | 4.94E-07 | 1.333114 |
| Activated.B.cellna | Eosinophilna | 0.540462 | 2.04E-39 | 3.242773 |
| Activated.B.cellna | Gamma.delta.T.cellna | 0.363233 | 4.21E-17 | 2.179398 |
| Activated.B.cellna | Immature..B.cellna | 0.898779 | ####### | 5.392677 |
| Activated.B.cellna | Immature.dendritic.cellna | 0.344891 | 1.81E-15 | 2.069344 |
| Activated.B.cellna | MDSCna | 0.703204 | 4.32E-76 | 4.219226 |
| Activated.B.cellna | Macrophagena | 0.655369 | 6.26E-63 | 3.932215 |
| Activated.B.cellna | Mast.cellna | 0.567033 | 4.79E-44 | 3.402195 |
| Activated.B.cellna | Monocytena | 0.681402 | 8.84E-70 | 4.08841 |
| Activated.B.cellna | Natural.killer.T.cellna | 0.606657 | 8.74E-52 | 3.639943 |
| Activated.B.cellna | Natural.killer.cellna | 0.579931 | 1.89E-46 | 3.479585 |
| Activated.B.cellna | Neutrophilna | 0.182843 | 3.77E-05 | 1.097061 |
| Activated.B.cellna | Plasmacytoid.dendritic.cellna | 0.351906 | 4.43E-16 | 2.111437 |
| Activated.B.cellna | Regulatory.T.cellna | 0.513466 | 4.08E-35 | 3.080799 |
| Activated.B.cellna | T.follicular.helper.cellna | 0.603997 | 3.13E-51 | 3.623983 |
| Activated.B.cellna | Type.1.T.helper.cellna | 0.751161 | 3.07E-92 | 4.506967 |
| Activated.B.cellna | Type.17.T.helper.cellna | 0.486369 | 3.61E-31 | 2.918215 |
| Activated.B.cellna | Type.2.T.helper.cellna | 0.491047 | 7.96E-32 | 2.946284 |
| Activated.CD4.T.cellna | Activated.CD8.T.cellna | 0.741325 | 1.23E-88 | 4.447953 |
| Activated.CD4.T.cellna | Activated.dendritic.cellna | 0.620943 | 7.51E-55 | 3.725659 |
| Activated.CD4.T.cellna | CD56bright.natural.killer.cellna | 0.259283 | 3.72E-09 | 1.555698 |
| Activated.CD4.T.cellna | Eosinophilna | 0.471083 | 4.29E-29 | 2.826498 |
| Activated.CD4.T.cellna | Gamma.delta.T.cellna | 0.365495 | 2.60E-17 | 2.19297 |
| Activated.CD4.T.cellna | Immature..B.cellna | 0.777455 | ####### | 4.664729 |
| Activated.CD4.T.cellna | Immature.dendritic.cellna | 0.319195 | 2.36E-13 | 1.915171 |
| Activated.CD4.T.cellna | MDSCna | 0.689235 | 5.54E-72 | 4.135412 |
| Activated.CD4.T.cellna | Macrophagena | 0.556158 | 4.22E-42 | 3.336951 |
| Activated.CD4.T.cellna | Mast.cellna | 0.406799 | 1.98E-21 | 2.440793 |
| Activated.CD4.T.cellna | Monocytena | 0.524703 | 7.37E-37 | 3.14822 |
| Activated.CD4.T.cellna | Natural.killer.T.cellna | 0.690266 | 2.81E-72 | 4.141599 |
| Activated.CD4.T.cellna | Natural.killer.cellna | 0.554791 | 7.33E-42 | 3.328744 |
| Activated.CD4.T.cellna | Plasmacytoid.dendritic.cellna | 0.290018 | 3.48E-11 | 1.740111 |
| Activated.CD4.T.cellna | Regulatory.T.cellna | 0.526628 | 3.65E-37 | 3.159767 |
| Activated.CD4.T.cellna | T.follicular.helper.cellna | 0.513859 | 3.56E-35 | 3.083157 |
| Activated.CD4.T.cellna | Type.1.T.helper.cellna | 0.705777 | 7.12E-77 | 4.234665 |
| Activated.CD4.T.cellna | Type.17.T.helper.cellna | 0.43029 | 4.83E-24 | 2.581738 |
| Activated.CD4.T.cellna | Type.2.T.helper.cellna | 0.646629 | 8.85E-61 | 3.879774 |
| Activated.CD8.T.cellna | Activated.dendritic.cellna | 0.629892 | 7.47E-57 | 3.779349 |
| Activated.CD8.T.cellna | CD56bright.natural.killer.cellna | 0.304904 | 2.92E-12 | 1.829427 |
| Activated.CD8.T.cellna | Eosinophilna | 0.489397 | 1.36E-31 | 2.936384 |
| Activated.CD8.T.cellna | Gamma.delta.T.cellna | 0.388617 | 1.52E-19 | 2.331705 |
| Activated.CD8.T.cellna | Immature..B.cellna | 0.826061 | ####### | 4.956365 |
| Activated.CD8.T.cellna | Immature.dendritic.cellna | 0.323038 | 1.17E-13 | 1.93823 |
| Activated.CD8.T.cellna | MDSCna | 0.837187 | ####### | 5.023124 |
| Activated.CD8.T.cellna | Macrophagena | 0.641761 | 1.30E-59 | 3.850564 |
| Activated.CD8.T.cellna | Mast.cellna | 0.470857 | 4.59E-29 | 2.825141 |
| Activated.CD8.T.cellna | Monocytena | 0.684479 | 1.23E-70 | 4.106873 |
| Activated.CD8.T.cellna | Natural.killer.T.cellna | 0.763915 | 3.59E-97 | 4.583492 |
| Activated.CD8.T.cellna | Natural.killer.cellna | 0.62366 | 1.88E-55 | 3.74196 |
| Activated.CD8.T.cellna | Plasmacytoid.dendritic.cellna | 0.232173 | 1.43E-07 | 1.393035 |
| Activated.CD8.T.cellna | Regulatory.T.cellna | 0.5897 | 2.42E-48 | 3.538202 |
| Activated.CD8.T.cellna | T.follicular.helper.cellna | 0.672006 | 3.16E-67 | 4.032035 |
| Activated.CD8.T.cellna | Type.1.T.helper.cellna | 0.765989 | 5.29E-98 | 4.595933 |
| Activated.CD8.T.cellna | Type.17.T.helper.cellna | 0.413569 | 3.67E-22 | 2.481413 |
| Activated.CD8.T.cellna | Type.2.T.helper.cellna | 0.539258 | 3.24E-39 | 3.235547 |
| Activated.dendritic.cellna | CD56bright.natural.killer.cellna | 0.403228 | 4.75E-21 | 2.419365 |
| Activated.dendritic.cellna | Eosinophilna | 0.590538 | 1.66E-48 | 3.543228 |
| Activated.dendritic.cellna | Gamma.delta.T.cellna | 0.625651 | 6.77E-56 | 3.753905 |
| Activated.dendritic.cellna | Immature..B.cellna | 0.734085 | 4.36E-86 | 4.404509 |
| Activated.dendritic.cellna | Immature.dendritic.cellna | 0.586582 | 9.89E-48 | 3.51949 |
| Activated.dendritic.cellna | MDSCna | 0.75439 | 1.85E-93 | 4.526341 |
| Activated.dendritic.cellna | Macrophagena | 0.733011 | 1.02E-85 | 4.398067 |
| Activated.dendritic.cellna | Mast.cellna | 0.618395 | 2.72E-54 | 3.710369 |
| Activated.dendritic.cellna | Monocytena | 0.630659 | 5.00E-57 | 3.783953 |
| Activated.dendritic.cellna | Natural.killer.T.cellna | 0.679587 | 2.80E-69 | 4.077521 |
| Activated.dendritic.cellna | Natural.killer.cellna | 0.614111 | 2.30E-53 | 3.684664 |
| Activated.dendritic.cellna | Neutrophilna | 0.428246 | 8.31E-24 | 2.569475 |
| Activated.dendritic.cellna | Plasmacytoid.dendritic.cellna | 0.575246 | 1.45E-45 | 3.451473 |
| Activated.dendritic.cellna | Regulatory.T.cellna | 0.748229 | 3.79E-91 | 4.489375 |
| Activated.dendritic.cellna | T.follicular.helper.cellna | 0.734065 | 4.43E-86 | 4.404388 |
| Activated.dendritic.cellna | Type.1.T.helper.cellna | 0.745902 | 2.72E-90 | 4.475414 |
| Activated.dendritic.cellna | Type.17.T.helper.cellna | 0.554539 | 8.11E-42 | 3.327235 |
| Activated.dendritic.cellna | Type.2.T.helper.cellna | 0.57422 | 2.26E-45 | 3.44532 |
| CD56bright.natural.killer.cellna | Eosinophilna | 0.31356 | 6.48E-13 | 1.881362 |
| CD56bright.natural.killer.cellna | Gamma.delta.T.cellna | 0.417037 | 1.53E-22 | 2.502222 |
| CD56bright.natural.killer.cellna | Immature..B.cellna | 0.347641 | 1.05E-15 | 2.085844 |
| CD56bright.natural.killer.cellna | Immature.dendritic.cellna | 0.403199 | 4.78E-21 | 2.419193 |
| CD56bright.natural.killer.cellna | MDSCna | 0.369038 | 1.21E-17 | 2.214228 |
| CD56bright.natural.killer.cellna | Macrophagena | 0.366252 | 2.21E-17 | 2.197513 |
| CD56bright.natural.killer.cellna | Mast.cellna | 0.401161 | 7.84E-21 | 2.406963 |
| CD56bright.natural.killer.cellna | Monocytena | 0.279976 | 1.71E-10 | 1.679859 |
| CD56bright.natural.killer.cellna | Natural.killer.T.cellna | 0.392845 | 5.67E-20 | 2.357072 |
| CD56bright.natural.killer.cellna | Natural.killer.cellna | 0.393602 | 4.75E-20 | 2.361615 |
| CD56bright.natural.killer.cellna | Neutrophilna | 0.299727 | 7.03E-12 | 1.798363 |
| CD56bright.natural.killer.cellna | Plasmacytoid.dendritic.cellna | 0.342849 | 2.72E-15 | 2.057095 |
| CD56bright.natural.killer.cellna | Regulatory.T.cellna | 0.430515 | 4.55E-24 | 2.583092 |
| CD56bright.natural.killer.cellna | T.follicular.helper.cellna | 0.47691 | 7.14E-30 | 2.86146 |
| CD56bright.natural.killer.cellna | Type.1.T.helper.cellna | 0.413153 | 4.08E-22 | 2.47892 |
| CD56bright.natural.killer.cellna | Type.17.T.helper.cellna | 0.289457 | 3.81E-11 | 1.736741 |
| CD56bright.natural.killer.cellna | Type.2.T.helper.cellna | 0.448886 | 2.92E-26 | 2.693314 |
| Eosinophilna | Gamma.delta.T.cellna | 0.564364 | 1.46E-43 | 3.386186 |
| Eosinophilna | Immature..B.cellna | 0.620624 | 8.83E-55 | 3.723747 |
| Eosinophilna | Immature.dendritic.cellna | 0.493687 | 3.36E-32 | 2.962122 |
| Eosinophilna | MDSCna | 0.593367 | 4.54E-49 | 3.560205 |
| Eosinophilna | Macrophagena | 0.621484 | 5.71E-55 | 3.728901 |
| Eosinophilna | Mast.cellna | 0.668631 | 2.48E-66 | 4.011785 |
| Eosinophilna | Monocytena | 0.564379 | 1.45E-43 | 3.386274 |
| Eosinophilna | Natural.killer.T.cellna | 0.522902 | 1.42E-36 | 3.137411 |
| Eosinophilna | Natural.killer.cellna | 0.608428 | 3.71E-52 | 3.650568 |
| Eosinophilna | Neutrophilna | 0.304188 | 3.30E-12 | 1.825128 |
| Eosinophilna | Plasmacytoid.dendritic.cellna | 0.547535 | 1.31E-40 | 3.285209 |
| Eosinophilna | Regulatory.T.cellna | 0.606923 | 7.69E-52 | 3.641537 |
| Eosinophilna | T.follicular.helper.cellna | 0.647099 | 6.81E-61 | 3.882595 |
| Eosinophilna | Type.1.T.helper.cellna | 0.668748 | 2.31E-66 | 4.012489 |
| Eosinophilna | Type.17.T.helper.cellna | 0.48486 | 5.84E-31 | 2.90916 |
| Eosinophilna | Type.2.T.helper.cellna | 0.522201 | 1.83E-36 | 3.133204 |
| Gamma.delta.T.cellna | Immature..B.cellna | 0.54156 | 1.34E-39 | 3.24936 |
| Gamma.delta.T.cellna | Immature.dendritic.cellna | 0.588542 | 4.09E-48 | 3.531249 |
| Gamma.delta.T.cellna | MDSCna | 0.659657 | 5.19E-64 | 3.957944 |
| Gamma.delta.T.cellna | Macrophagena | 0.69455 | 1.62E-73 | 4.167301 |
| Gamma.delta.T.cellna | Mast.cellna | 0.672382 | 2.51E-67 | 4.034289 |
| Gamma.delta.T.cellna | Monocytena | 0.504165 | 1.01E-33 | 3.024988 |
| Gamma.delta.T.cellna | Natural.killer.T.cellna | 0.586155 | 1.20E-47 | 3.51693 |
| Gamma.delta.T.cellna | Natural.killer.cellna | 0.636412 | 2.36E-58 | 3.81847 |
| Gamma.delta.T.cellna | Neutrophilna | 0.383638 | 4.77E-19 | 2.301825 |
| Gamma.delta.T.cellna | Plasmacytoid.dendritic.cellna | 0.648082 | 3.93E-61 | 3.888489 |
| Gamma.delta.T.cellna | Regulatory.T.cellna | 0.790242 | ####### | 4.741454 |
| Gamma.delta.T.cellna | T.follicular.helper.cellna | 0.762369 | 1.48E-96 | 4.574213 |
| Gamma.delta.T.cellna | Type.1.T.helper.cellna | 0.662435 | 1.01E-64 | 3.974612 |
| Gamma.delta.T.cellna | Type.17.T.helper.cellna | 0.374718 | 3.51E-18 | 2.248309 |
| Gamma.delta.T.cellna | Type.2.T.helper.cellna | 0.540478 | 2.03E-39 | 3.24287 |
| Immature..B.cellna | Immature.dendritic.cellna | 0.470721 | 4.79E-29 | 2.824327 |
| Immature..B.cellna | MDSCna | 0.855207 | ####### | 5.131243 |
| Immature..B.cellna | Macrophagena | 0.759232 | 2.52E-95 | 4.555392 |
| Immature..B.cellna | Mast.cellna | 0.636201 | 2.64E-58 | 3.817203 |
| Immature..B.cellna | Monocytena | 0.766765 | 2.57E-98 | 4.600588 |
| Immature..B.cellna | Natural.killer.T.cellna | 0.754392 | 1.85E-93 | 4.526352 |
| Immature..B.cellna | Natural.killer.cellna | 0.708902 | 7.76E-78 | 4.25341 |
| Immature..B.cellna | Neutrophilna | 0.273485 | 4.62E-10 | 1.640908 |
| Immature..B.cellna | Plasmacytoid.dendritic.cellna | 0.455187 | 4.81E-27 | 2.731123 |
| Immature..B.cellna | Regulatory.T.cellna | 0.705424 | 9.12E-77 | 4.232547 |
| Immature..B.cellna | T.follicular.helper.cellna | 0.767511 | 1.28E-98 | 4.605064 |
| Immature..B.cellna | Type.1.T.helper.cellna | 0.85988 | ####### | 5.159278 |
| Immature..B.cellna | Type.17.T.helper.cellna | 0.548899 | 7.67E-41 | 3.293391 |
| Immature..B.cellna | Type.2.T.helper.cellna | 0.619187 | 1.82E-54 | 3.715122 |
| Immature.dendritic.cellna | MDSCna | 0.519635 | 4.59E-36 | 3.117811 |
| Immature.dendritic.cellna | Macrophagena | 0.641727 | 1.33E-59 | 3.850361 |
| Immature.dendritic.cellna | Mast.cellna | 0.632225 | 2.19E-57 | 3.79335 |
| Immature.dendritic.cellna | Monocytena | 0.506925 | 3.94E-34 | 3.04155 |
| Immature.dendritic.cellna | Natural.killer.T.cellna | 0.398735 | 1.40E-20 | 2.392413 |
| Immature.dendritic.cellna | Natural.killer.cellna | 0.519624 | 4.61E-36 | 3.117744 |
| Immature.dendritic.cellna | Neutrophilna | 0.392088 | 6.78E-20 | 2.35253 |
| Immature.dendritic.cellna | Plasmacytoid.dendritic.cellna | 0.576483 | 8.51E-46 | 3.458896 |
| Immature.dendritic.cellna | Regulatory.T.cellna | 0.562454 | 3.22E-43 | 3.374726 |
| Immature.dendritic.cellna | T.follicular.helper.cellna | 0.5961 | 1.28E-49 | 3.5766 |
| Immature.dendritic.cellna | Type.1.T.helper.cellna | 0.549369 | 6.37E-41 | 3.296213 |
| Immature.dendritic.cellna | Type.17.T.helper.cellna | 0.437623 | 6.69E-25 | 2.625736 |
| Immature.dendritic.cellna | Type.2.T.helper.cellna | 0.387782 | 1.84E-19 | 2.326693 |
| MDSCna | Macrophagena | 0.868064 | ####### | 5.208382 |
| MDSCna | Mast.cellna | 0.657088 | 2.32E-63 | 3.94253 |
| MDSCna | Monocytena | 0.780762 | ####### | 4.684571 |
| MDSCna | Natural.killer.T.cellna | 0.8072 | ####### | 4.843202 |
| MDSCna | Natural.killer.cellna | 0.788103 | ####### | 4.728616 |
| MDSCna | Neutrophilna | 0.20461 | 3.80E-06 | 1.227659 |
| MDSCna | Plasmacytoid.dendritic.cellna | 0.481339 | 1.78E-30 | 2.888036 |
| MDSCna | Regulatory.T.cellna | 0.879884 | ####### | 5.279305 |
| MDSCna | T.follicular.helper.cellna | 0.843347 | ####### | 5.060081 |
| MDSCna | Type.1.T.helper.cellna | 0.886486 | ####### | 5.318915 |
| MDSCna | Type.17.T.helper.cellna | 0.441101 | 2.58E-25 | 2.646604 |
| MDSCna | Type.2.T.helper.cellna | 0.565377 | 9.57E-44 | 3.392262 |
| Macrophagena | Mast.cellna | 0.774332 | ####### | 4.645992 |
| Macrophagena | Monocytena | 0.768046 | 7.76E-99 | 4.608277 |
| Macrophagena | Natural.killer.T.cellna | 0.686703 | 2.91E-71 | 4.120219 |
| Macrophagena | Natural.killer.cellna | 0.759033 | 3.01E-95 | 4.554199 |
| Macrophagena | Neutrophilna | 0.263758 | 1.96E-09 | 1.582546 |
| Macrophagena | Plasmacytoid.dendritic.cellna | 0.596661 | 9.90E-50 | 3.579966 |
| Macrophagena | Regulatory.T.cellna | 0.819701 | ####### | 4.918205 |
| Macrophagena | T.follicular.helper.cellna | 0.807453 | ####### | 4.84472 |
| Macrophagena | Type.1.T.helper.cellna | 0.835289 | ####### | 5.011732 |
| Macrophagena | Type.17.T.helper.cellna | 0.494568 | 2.51E-32 | 2.967406 |
| Macrophagena | Type.2.T.helper.cellna | 0.511711 | 7.54E-35 | 3.070265 |
| Mast.cellna | Monocytena | 0.655225 | 6.81E-63 | 3.931349 |
| Mast.cellna | Natural.killer.T.cellna | 0.56749 | 3.95E-44 | 3.404941 |
| Mast.cellna | Natural.killer.cellna | 0.669508 | 1.46E-66 | 4.017045 |
| Mast.cellna | Neutrophilna | 0.37157 | 7.01E-18 | 2.229423 |
| Mast.cellna | Plasmacytoid.dendritic.cellna | 0.660199 | 3.78E-64 | 3.961197 |
| Mast.cellna | Regulatory.T.cellna | 0.686582 | 3.14E-71 | 4.119489 |
| Mast.cellna | T.follicular.helper.cellna | 0.764022 | 3.25E-97 | 4.584135 |
| Mast.cellna | Type.1.T.helper.cellna | 0.71982 | 2.66E-81 | 4.318922 |
| Mast.cellna | Type.17.T.helper.cellna | 0.512293 | 6.15E-35 | 3.073755 |
| Mast.cellna | Type.2.T.helper.cellna | 0.506389 | 4.74E-34 | 3.038332 |
| Monocytena | Natural.killer.T.cellna | 0.634373 | 7.01E-58 | 3.806238 |
| Monocytena | Natural.killer.cellna | 0.6878 | 1.42E-71 | 4.126801 |
| Monocytena | Neutrophilna | 0.238571 | 6.29E-08 | 1.431423 |
| Monocytena | Plasmacytoid.dendritic.cellna | 0.450495 | 1.85E-26 | 2.702971 |
| Monocytena | Regulatory.T.cellna | 0.682066 | 5.79E-70 | 4.092394 |
| Monocytena | T.follicular.helper.cellna | 0.701034 | 1.95E-75 | 4.206207 |
| Monocytena | Type.1.T.helper.cellna | 0.760124 | 1.13E-95 | 4.560744 |
| Monocytena | Type.17.T.helper.cellna | 0.504137 | 1.02E-33 | 3.024822 |
| Monocytena | Type.2.T.helper.cellna | 0.468843 | 8.47E-29 | 2.813061 |
| Natural.killer.T.cellna | Natural.killer.cellna | 0.72519 | 4.55E-83 | 4.35114 |
| Natural.killer.T.cellna | Plasmacytoid.dendritic.cellna | 0.37885 | 1.40E-18 | 2.273099 |
| Natural.killer.T.cellna | Regulatory.T.cellna | 0.724719 | 6.53E-83 | 4.348313 |
| Natural.killer.T.cellna | T.follicular.helper.cellna | 0.717071 | 2.05E-80 | 4.302428 |
| Natural.killer.T.cellna | Type.1.T.helper.cellna | 0.80736 | ####### | 4.84416 |
| Natural.killer.T.cellna | Type.17.T.helper.cellna | 0.416347 | 1.82E-22 | 2.49808 |
| Natural.killer.T.cellna | Type.2.T.helper.cellna | 0.637819 | 1.11E-58 | 3.826917 |
| Natural.killer.cellna | Plasmacytoid.dendritic.cellna | 0.469716 | 6.50E-29 | 2.818296 |
| Natural.killer.cellna | Regulatory.T.cellna | 0.762366 | 1.48E-96 | 4.574194 |
| Natural.killer.cellna | T.follicular.helper.cellna | 0.78582 | ####### | 4.714918 |
| Natural.killer.cellna | Type.1.T.helper.cellna | 0.879625 | ####### | 5.277748 |
| Natural.killer.cellna | Type.17.T.helper.cellna | 0.500327 | 3.70E-33 | 3.001964 |
| Natural.killer.cellna | Type.2.T.helper.cellna | 0.605336 | 1.65E-51 | 3.632016 |
| Neutrophilna | Plasmacytoid.dendritic.cellna | 0.539349 | 3.13E-39 | 3.236096 |
| Neutrophilna | Regulatory.T.cellna | 0.277721 | 2.42E-10 | 1.666324 |
| Neutrophilna | T.follicular.helper.cellna | 0.377523 | 1.89E-18 | 2.265136 |
| Neutrophilna | Type.1.T.helper.cellna | 0.177339 | 6.47E-05 | 1.064036 |
| Neutrophilna | Type.17.T.helper.cellna | 0.46141 | 7.81E-28 | 2.768461 |
| Neutrophilna | Type.2.T.helper.cellna | 0.292636 | 2.27E-11 | 1.755813 |
| Plasmacytoid.dendritic.cellna | Regulatory.T.cellna | 0.616607 | 6.65E-54 | 3.699641 |
| Plasmacytoid.dendritic.cellna | T.follicular.helper.cellna | 0.596433 | 1.10E-49 | 3.578596 |
| Plasmacytoid.dendritic.cellna | Type.1.T.helper.cellna | 0.536671 | 8.67E-39 | 3.220028 |
| Plasmacytoid.dendritic.cellna | Type.17.T.helper.cellna | 0.472554 | 2.74E-29 | 2.835326 |
| Plasmacytoid.dendritic.cellna | Type.2.T.helper.cellna | 0.408561 | 1.28E-21 | 2.451368 |
| Regulatory.T.cellna | T.follicular.helper.cellna | 0.838827 | ####### | 5.032961 |
| Regulatory.T.cellna | Type.1.T.helper.cellna | 0.812343 | ####### | 4.874057 |
| Regulatory.T.cellna | Type.17.T.helper.cellna | 0.409918 | 9.16E-22 | 2.459507 |
| Regulatory.T.cellna | Type.2.T.helper.cellna | 0.583367 | 4.15E-47 | 3.500203 |
| T.follicular.helper.cellna | Type.1.T.helper.cellna | 0.854775 | ####### | 5.12865 |
| T.follicular.helper.cellna | Type.17.T.helper.cellna | 0.523131 | 1.30E-36 | 3.138784 |
| T.follicular.helper.cellna | Type.2.T.helper.cellna | 0.620518 | 9.32E-55 | 3.723111 |
| Type.1.T.helper.cellna | Type.17.T.helper.cellna | 0.56963 | 1.60E-44 | 3.417782 |
| Type.1.T.helper.cellna | Type.2.T.helper.cellna | 0.673268 | 1.45E-67 | 4.039608 |
| Type.17.T.helper.cellna | Type.2.T.helper.cellna | 0.487037 | 2.91E-31 | 2.922221 |
